# A Novel Multi-Tiered Hybrid Virtual Screening Pipeline for the Discovery of WDR5-MLL1 Interaction Disruptors in Precision Cancer Therapy

**DOI:** 10.1021/acsomega.5c02521

**Published:** 2025-09-29

**Authors:** Anwar Abuelrub, Ismail Erol, Serdar Durdağı

**Affiliations:** † Laboratory for Innovative Drugs (Lab4IND), Computational Drug Design Center (HİTMER), 52946 Bahçeşehir University, İstanbul 34734, Türkiye; ‡ Computational Biology and Molecular Simulations Laboratory, Department of Biophysics, School of Medicine, Bahçeşehir University, İstanbul 34734, Türkiye; § Graduate School of Natural and Applied Sciences, Artificial Intelligence Program, Bahçeşehir University, İstanbul 34353, Türkiye; ∥ Department of Analytical Chemistry, School of Pharmacy, Bahçeşehir University, İstanbul 34353, Türkiye; ⊥ Molecular Therapy Laboratory, Department of Pharmaceutical Chemistry, School of Pharmacy, Bahçeşehir University, İstanbul 34353, Türkiye

## Abstract

WD Repeat-containing protein 5 (WDR5) is a critical companion for the mixed lineage leukemia (MLL) complex, essential for epigenetic regulation and implicated in various cancers, particularly leukemia. Overexpression of WDR5 in malignant tissues is linked to poor clinical outcomes and enhanced cancer cell proliferation. Its interaction with the MLL1 protein occurs via the WDR5 protein, which is vital for the MLL complex’s methyltransferase activity. Recent studies highlight the WIN site as a promising therapeutic target, especially for MLL-rearranged leukemia. In this study, we investigated the structural dynamics of the WDR5-MLL1 complex and aimed to identify potential small-molecule inhibitors targeting the WIN site, to develop novel therapeutic strategies for leukemia and other WDR5 protein-dysregulated cancers. Utilizing the crystal structures of the WDR5 and MLL1, we screened around one million synthetically available compounds from ChemDiv, Enamine, and Specs small molecule libraries. The computational analysis was conducted through comprehensive all-atom molecular dynamics (MD) simulations to evaluate ligand–receptor interaction affinities and involved binding residues. The simulations revealed key participating amino acid residues while quantifying binding affinities using the Molecular Mechanics-Generalized Born Surface Area (MM-GBSA) approach. Steered molecular dynamics (sMD) simulations were further conducted to assess the stability of ligand–receptor interactions of the selected top-compounds. Additionally, novel potential compounds were generated using BRICS fragmentation and Monte Carlo tree search algorithms. Our analysis revealed diverse interaction patterns and potential inhibitory mechanism among the screened compounds. Several compounds, such as Z88418521 and Z116334910, displayed stronger predicted binding affinities than the reference molecule IA9, exhibiting competitive and allosteric modulation of the WDR5-MLL1 complex interaction. A thorough analysis of WDR5 protein and WDR5-MLL1 interactions and their conformational changes offered valuable perspectives on targeting the WDR5-MLL1 complex interaction. Thus, this study profiles the molecular alterations that occur during WDR5-MLL1 complex inhibition, offering crucial mechanistic insights that establish a solid framework for developing targeted treatments for MLL-rearranged leukemia. The distinctive binding characteristics and conformational dynamics exhibited by the identified compounds provide a compelling foundation for future experimental approaches to leukemia intervention.

## Graphical abstract

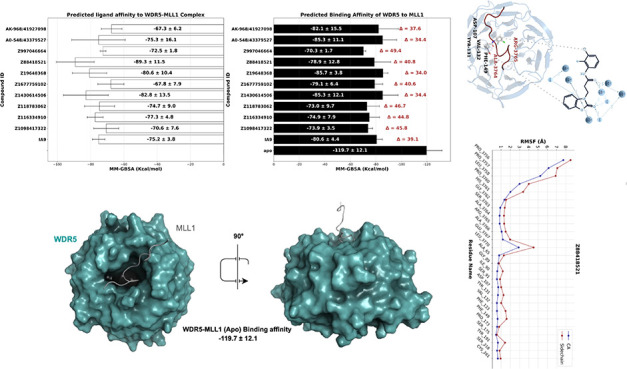

## 1 Introduction

In recent decades, structural and functional alterations in pathways associated with epigenomic activity have been closely linked to cancer mechanisms.^1^ These changes include not only methylation but also acetylation, phosphorylation, and ubiquitination, all contributing to chemical modifications within the coding genome.^2–6^ The “Histone Code” hypothesis offers a framework to understand the interplay between the genetic code, regulatory proteins, and histone post-translational modifications (PTMs).^7^ Methylation of histone N-terminal tails plays a pivotal role in transcriptional regulation and gene expression.^3,8,9^ Disruptions in proteins involved in epigenetic modifications can impact processes beyond the coding genome, contributing to tumorigenesis and cancer progression.^10^ A significant player in this context is WDR5 protein, that identified as a mediator in leukemia. WDR5 protein acts as an effector molecule that specifically recognizes methylation at histone H3 lysine 4 (H3K4).^11^ Recent studies underscore the critical oncogenic role of WDR5 protein in MLL-rearranged leukemia, highlighting its potential as a promising therapeutic target.^12–14^

### 1.1 Structure and Function of the WDR5 Protein

WDR5 protein adopts a seven-bladed propeller fold, with each blade comprising 40–60 amino acid residues. Each blade features a four-stranded antiparallel β-sheets, presenting extensive exposed surfaces that facilitate its involvement in large multiprotein complexes.^15^ WDR5 protein interacts with key complexes, including c-Myc,^17^ N-Myc,^13^ CUL4-DDB1,^18^ and MLL,^15^ primarily through two binding sites. The first is the WDR5-binding motif (WBM) site, which mediates interactions with N-Myc and RbBP5-WDR5.^15^ The second is the WIN site, which enables direct binding between WDR5 protein and MLL1 protein.^19^ The highly conserved structure of the WDR5 protein, supported by a robust hydrogen bonding network between WD domains, is critical for its role in chromatin-related cellular processes, including vesicle trafficking, RNA processing, cytokinesis, DNA replication, protein stability, and transcription regulation.^20–22^ As a structural scaffold, WDR5 protein facilitates the assembly of epigenetic complexes, including the MLL/SET histone methyltransferase (HMT) complex, which catalyzes di- and trimethylation of H3K4. WDR5 protein also contributes to other complexes, such as nonspecific lethal (NSL) and Ada2-containing (ATAC) histone acetyltransferases.^23^ Beyond its role in chromatin modification, WDR5 protein influences epigenetic compensation mechanisms. Methylated histones exhibit increased mass, hydrophobicity, and preserved charge, which regulate genes involved in processes such as cell proliferation, apoptosis, and cell cycle progression.^16^ WDR5 protein further stabilizes H3K4 methylation, maintaining structural integrity even in the absence of the MLL complex. Without WDR5 protein, MLL1 protein‘s partners RbBP5, ASH2L, and DPY30 fail to stably associate with MLL, disrupting Complex of Proteins Associated with SET1 (COMPASS) complex formation and function.^24^ WDR5 protein is indispensable for the MLL complex, as it is required both for histone H3 methylation and for binding to histone H3 tails. Contrary to earlier assumptions, the methylated LYS4 side chain minimally interacts with WDR5 protein.^15^ Further, the significant findings of Siladi et al. (2022) revealed that WDR5’s role in H3K4 methylation is more nuanced than previously understood. Their research demonstrates that WDR5 WIN site inhibition affects only a specific subset of WDR5 functions, and importantly, that the H3K4me changes resulting from WDR5 depletion do not adequately explain the observed transcriptional responses. This critical observation regarding WDR5’s contribution to H3K4 methylation is context-dependent and secondary to its other cellular functions.^25^ Structural analyses show that WDR5 protein interacts with MLL1 protein via the same pocket that binds H3.^26^ Full MLL1 protein activation requires caspase cleavage into *N*- and C-terminal fragments, which then reassemble into a stable protein complex.^27^ Previous studies indicated that the MLL1/SET domain alone exhibits limited catalytic activity in the absence of MLL partner proteins.^27,28^ However, integration of MLL1 protein with COMPASS complex subunits significantly enhances its methyltransferase activity (MTA), enabling optimal H3K4 methylation. This interaction triggers conformational changes in the SET domain, aligning the catalytic site for peak efficiency.^28^ Additionally, WDR5 protein serves as a reader of H3K4 methylation, directly recognizing the methylation state via the WIN site. Specifically, the H3 tail anchors at the WIN site through ARG2, where cation–π stacking interactions between the guanidinium group of arginine and WDR5 protein residues PHE133 and PHE263 facilitate methylation state recognition.^29,30^

### 1.2 WDR5 Protein‘s Role in Cell Proliferation, Apoptosis, Cell Cycle, and Immunogenetics

Previous studies have shown that the WDR5 protein localizes to specific loci enriched with ribosomal protein genes.^31^ Depletion of WDR5 protein from these loci induces translational stress and enhances the translation of several genes, including p53, leading to the activation of p53-dependent apoptosis.^30^ WDR5 protein is also critical for H3K4 methylation and regulates the transcription of target genes involved in pluripotency, tumor progression, and malignancy. Acting as a key oncogenic mediator, WDR5 protein is closely linked to the development and progression of multiple cancers, such as bladder, prostate, colon cancer, and leukemia.^31,32^ Silencing WDR5 protein has been shown to reduce tumor cell proliferation and inhibit cancer progression, particularly in breast cancer cells.^32^ Furthermore, WDR5 knockdown suppresses cyclin E1, cyclin E2, and UHMK1, leading to G0/G1 cell cycle arrest and downregulation of cyclin B1, which disrupts the G2-to-M phase transition.^33^ These findings emphasize the significance of epigenetic regulation in controlling immune responses.^34^ The methylation status of H3K4 is a key regulator of leukemia stem cell oncogenic capacity.^35^ Previous studies demonstrated that the inhibitor MM-401 effectively blocks the interaction between MLL1 and WDR5 proteins, preventing complex formation and inhibiting MLL1 protein activity.^36^ This inhibition results in cell cycle arrest, apoptosis, and myeloid differentiation, thereby halting cancer cell growth.^36^ In T-cell acute lymphoblastic leukemia (T-ALL), a hematological malignancy, the accumulation of genetic defects during T-cell development leads to abnormal proliferation and differentiation of progenitor cells, resulting in leukocytosis and infiltration into lymph nodes and organs.^37^ WDR5 protein is frequently overexpressed in various cancers and correlates with poor prognosis, increased proliferation, and other pathological traits associated with malignancy.^38^ Additionally, WDR5 protein acts as a coactivator of key transcription factors like c-Myc,^17^ enhancing gene expression and promoting tumorigenesis. Studies have shown that elevated WDR5 protein expression significantly increases the risk of leukemia, with particularly high levels detected in T-ALL patients compared to other conditions.^38–40^ WDR5 protein also modulates cyclin D expression and regulates DNA damage response via H3K4me3 modification. Therefore, targeting WDR5 protein emerges as a promising cancer therapy strategy, as its inhibition effectively suppresses tumor development, particularly in leukemia.^39,41^

### 1.3 WDR5 Protein as a Therapeutic Target in Cancer

WDR5 protein has emerged as a compelling therapeutic target due to its overexpression across various malignancies and its pivotal role in promoting oncogenic processes, including epithelial-to-mesenchymal transition (EMT),^41^ cell migration,^42^ and its interaction with oncogenic agents such as MLL-fusion oncoproteins.^36^ Recent investigations utilizing the human MLL cell line MV-4-11, which expresses the MLL-AF4 fusion oncogene, identified a small-molecule inhibitor capable of targeting the WDR5 WIN site.^43^ This study demonstrated that WIN site inhibitors effectively disrupted WDR5 protein’s association with chromatin, impairing its transcriptional regulatory activity while leaving H3K4me3 levels unchanged. This approach builds on pioneering work by Karatas et al. (2013), who developed a strategic alternative to direct SET domain inhibition. By targeting the WDR5 protein interaction interface to prevent MLL1 protein methyltransferase complex assembly, they created a more selective intervention that avoided potential off-target effects on other methyltransferases, offering potentially greater selectivity.^44^ Moreover, treatment with WDR5 WIN site inhibitors significantly reduced the protein synthesis capacity of MV-4-11 cells, induced nucleolar stress, and promoted apoptosis through increased p53 production. Collectively, these findings highlight that targeting the WDR5 protein can effectively suppress cancer cell viability by arresting the cell cycle, inducing apoptosis, and impairing ribosomal protein synthesis pathways.^39,45^

The inhibition of the WDR5-MLL1 complex interaction has emerged as a promising therapeutic approach for various cancers, particularly leukemias. Wang’s research group^46^ developed MM-589, a macrocyclic peptidomimetic that binds to WDR5 and blocks the WDR5-MLL complex interaction, inhibiting MLL H3K4 methyltransferase activity. Fesik’s research group^47–49^ made significant advancements through multiple studies, progressing from discovering dihydroisoquinolinone bicyclic core compounds with picomolar binding affinity to developing orally bioavailable WDR5 inhibitors with improved druglike properties, and ultimately creating potent WDR5 protein inhibitors demonstrating efficacy and safety in preclinical animal models. Meanwhile, Guo’s research group^50^ used click chemistry for bioisosterism to develop DDO-2093, a phenyltriazole scaffold inhibitor with high binding affinity and improved drug-like properties that showed significant tumor suppression in MV4–11 xenograft models with a favorable safety profile. While Wang’s work pioneered peptidomimetic approaches, Fesik’s studies represent the most comprehensive progression from *in vitro* to *in vivo* models with oral bioavailability, collectively advancing WDR5-MLL1 complex inhibition as a viable cancer therapeutic strategy.^46–50^

In the current study, we focused on the structural dynamics of the WDR5-MLL1 complex, aiming to identify novel small-molecule inhibitors that specifically target the WDR5 WIN site. By leveraging advanced computational biology and molecular simulations approaches, our goal is to develop potential innovative therapeutic strategies to treat leukemia and other cancer types driven mainly by WDR5 protein dysregulation.

## 2 Methods

The comprehensive approach utilized in this study is illustrated in Figure 1, summarizing the key computational steps and analyses performed.

**Figure 1:**
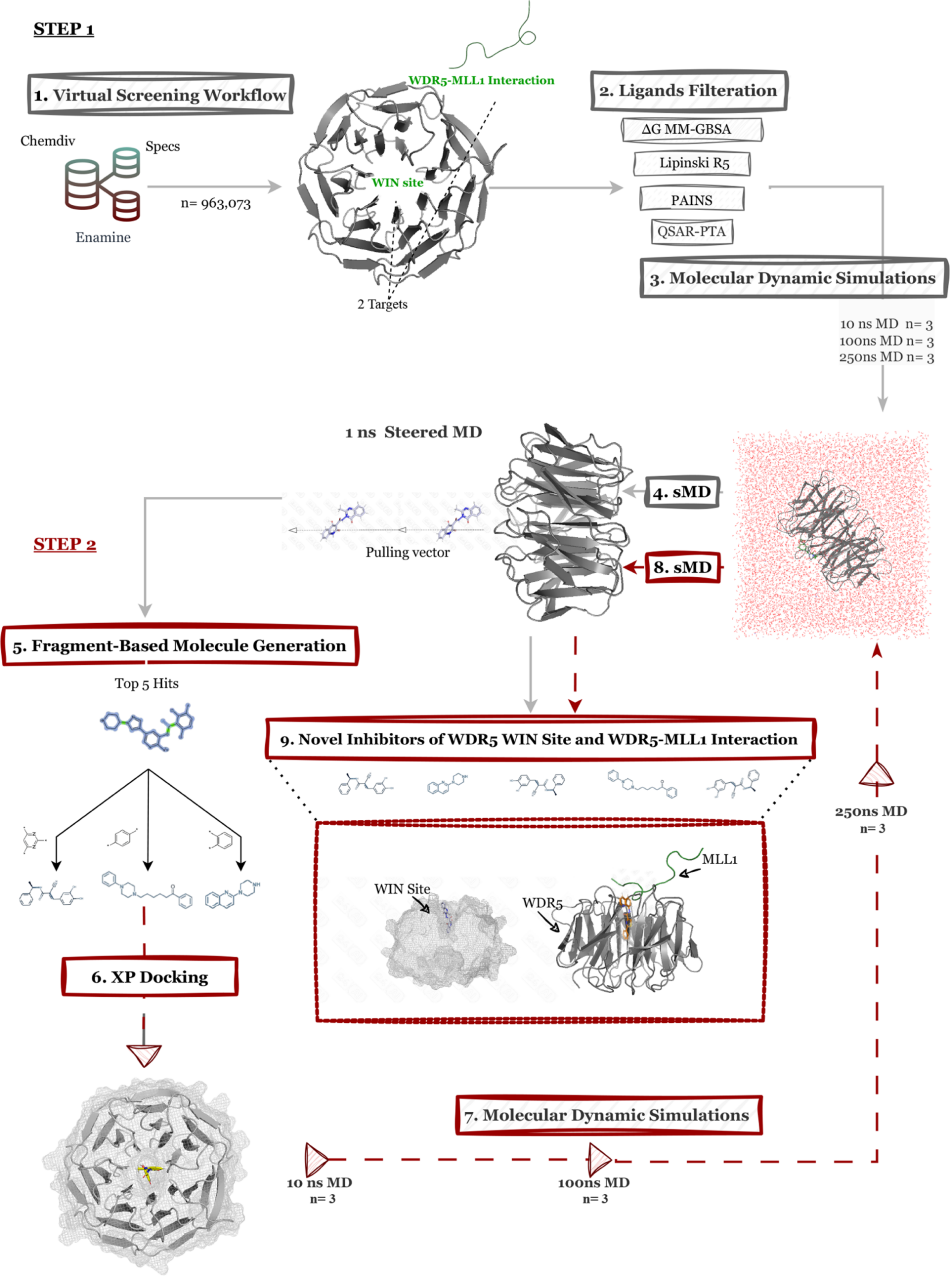
Workflow of the study, outlining the methodological framework carried out through the analysis process.

### 2.1 Selection and Preparation of the Target Protein

The 3D molecular structures of the WDR5 protein complexed with the small molecule IA9 were retrieved from the Protein Data Bank (PDB) with the corresponding PDB ID: 4IA9,^51^ while the MLL1 protein structure was obtained under PDB code: 4ESG.^52^ Each protein structure was prepared using the Protein Preparation Wizard (PrepWiz) tool within the Maestro molecular modeling suite (Schrödinger LLC). PrepWiz assigned appropriate bond orders, added hydrogen atoms and any missing amino acid residues, and generated the necessary disulfide bridges.^53^ The PROPKA was employed to determine the protonation states of amino acid residues at physiological pH conditions.^54^ Following preparation, the WDR5-ligand and WDR5-MLL1-ligand complexes underwent energy minimization using OPLS3e force field parameters.^55^ These optimized target structures were subsequently utilized for molecular docking and MD simulations.^56^

### 2.2 Ligand Preparation and Virtual Screening Workflow (VSW)

To identify potential inhibitors targeting the WDR5-MLL1 complex, we performed an extensive screening of multiple compound databases. Specifically, we evaluated the ChemDiv, Enamine, and Specs libraries. This included approximately 300,000 compounds from the ChemDiv “300 K Representative Screening Compounds Library”,^57^ around 460,000 small molecules from the Enamine “Hit Locator Library (HLL-460)”,^58^ and about 200,000 compounds from the “Specs_SC_10_mg_Apr2023” collection.^59^ All libraries were processed using the LigPrep module in the Maestro molecular modeling package.^60^ Epik was utilized to generate protonation states for fragments and ligands at pH 7.4.^54^ In the molecular docking studies the crystal structures of WDR5 and MLL1 complex (PDB IDs: 4IA9 and 4ESG) were used. The active site of WDR5 protein was identified based on Chen et al.^50^ and docking grid maps were generated accordingly. The Glide docking module was used with the following protocols:^61^ (i) High-Throughput Virtual Screening (HTVS) for initial filtering; (ii) Glide/SP (Standard Precision) docking was performed for more refined screening, selecting complexes with top docking scores for further refinement; (iii) Glide/XP (Extra Precision) docking was employed to redock the final selected ligands. For each ligand, ten docking poses were generated. (iv) Finally, Prime MM-GBSA (Molecular Mechanics-Generalized Born Surface Area) calculations were performed on the top-scoring XP poses to estimate binding free energies and identify the most stable protein–ligand complexes. Throughout the filtration process, a rigorous 10% threshold was applied to prioritize the most promising candidates. Specifically, the top 10% of compounds from the initial database, based on their docking score ranking, were selected for progression to the next stage of screening.

### 2.3 Evaluation of Drug-Likeness Based on Lipinski’s Rule of Five (Ro5)

Assessing the drug-likeness of a compound is a crucial step in the drug discovery process.^62^ This evaluation is guided by Lipinski’s Ro5, a set of criteria defining the physicochemical properties desirable for an orally active drug. According to Ro5, an ideal drug candidate should meet the following requirements: Molecular weight below 500 Da; logP value not exceeding 5; no more than 5 hydrogen bond donors; and no more than 10 hydrogen bond acceptors. Given that the compound libraries were pre-enriched with drug-like molecules, only a small proportion of compounds, fewer than 5%, were excluded during this filtering step.

### 2.4 PAINS Filter

To ensure the reliability of our results, all compounds from the ChemDiv, Enamine, and Specs libraries underwent additional filtering using the pan-assay interference compounds (PAINS) filter.^63^ This step was implemented to eliminate compounds with specific chemical motifs prone to interact with multiple biological targets, which could lead to artifacts or false-positive results in biological assays. PAINS compounds are known to cause nonspecific interactions or undesirable effects that may compromise the accuracy of experimental outcomes.

### 2.5 Binary QSAR Models

The Clarivate Analytic’s MetaCore/MetaDrug platform^64^ was employed to evaluate the potential of the top-ranked compounds as anticancer therapeutic agents. Each compound underwent binary quantitative structure–activity relationship (QSAR) analysis, utilizing the platform’s Cancer-QSAR model for prediction. The model’s output values were normalized between 0 and 1, with 0 indicating inactivity and 1 representing an active compound with anticancer potential. Compounds with a predicted activity score above 0.5 were selected for further investigation through MD simulations to validate their therapeutic potential. (Model description: Training set *N* = 886, Test set *N* = 167, Sensitivity= 0.89, Specificity = 0.83, Accuracy = 0.86, MCC = 0.72)

### 2.6 MD Simulations and MM-GBSA Calculations

Following docking experiments, which provided an initial static orientation for ligand interactions within the active site of the target protein, MD simulations were conducted to further explore the structural and dynamic changes of the complexes. MD simulations were performed on the apo form of the target structures and selected potent molecules in complex with WDR5 protein and the WDR5-MLL1 complex to evaluate their behavior over different time scales. These simulations aimed to measure the average atomic displacement relative to a reference point, assessing the stability of the target complexes. The Desmond program was used for the MD simulations. The molecular systems were initially solvated in a TIP3P water model using orthorhombic box with 0.15 M NaCl salt concentration.^65^ An initial energy minimization step was performed, followed by system equilibration using Desmond’s default protocols. Atomistic interactions were modeled using the OPLS3 force field.^55^ MD simulations were performed at 310 K over varying durations and three replicates:^66^ (i) 10 ns short simulations; (ii) 100 ns long simulations; (iii) 250 ns extended simulations. Throughout the simulations, 1000 trajectory frames were collected for both the short and long MD runs. The MM-GBSA method^67^ was employed to calculate average binding free energies for the apo form, WDR5 protein–ligand and the WDR5-MLL1-ligand complexes of the selected hits. Triplicate MM-GBSA averages were computed for 1000 frames across the 10, 100, and 250 ns MD simulations.

### 2.7 sMD Simulations

sMD simulations are computational techniques designed to study the unbinding process of a ligand from a protein or protein–protein complex. In these simulations, a time-dependent external force is applied to gradually pull the ligand away from the protein or complex. Specifically, the force will be directed toward a reference point on the ligand. During this process, the force exerted by the protein on the ligand is measured, providing valuable insights into the energetics and mechanics of the unbinding pathway. The simulations were performed using the GROMACS package (v.2022) with the CHARMM36m force field, ensuring accurate modeling of interatomic interactions and system energetics. The steepest descent (SD) algorithm is used to eliminate unfavorable atomic overlaps or high-energy conformations in the starting structure. For the sMD simulations, a precise molecular orientation approach was applied to accurately measure unbinding forces. All molecular structures were initially aligned to a consistent reference point within the binding site, establishing a uniform baseline for pulling vector measurements. For the WDR5 protein system, simulations proceeded along the *Y*-axis with a force constant of the spring was set to 200 kJ·mol^−1^·nm^−2^, while the WDR5-MLL1 complex was simulated along the *X*-axis using a higher force constant of 300 kJ·^−1^·nm^−2^. Both systems underwent two distinct simulation phases: a 1000 ps phase with ligand pulling at constant velocity of 0.1 Å/ps and a 3000 ps phase with slower ligand pulling at 0.01 Å/ps. This methodological framework ensured optimal alignment between the pulling vector and the unbinding energy landscape, enabling accurate characterization of interactions between the ligand and the WDR5 protein, as well as within the WDR5-MLL1 complex.

### 2.8 Fragment-Based Molecule Design: Utilizing BRICS and Monte Carlo Tree Search (MCTS)

To design novel molecules targeting both the WDR5 protein and the WDR5-MLL1 complex, we select the most promising compounds: five candidates targeting the WDR5 protein (Z3687064797, Z3687067367, Z1551692094, Z1754517473, and Z3687055598) and five targeting the WDR5-MLL1 complex (Z116334910, Z118783062, Z88418521, Z997046664, and Z1098417322). These compounds, along with the reference compound IA9, serve as starting points for finding new analog molecules. The Breaks of Retrosynthetically Interesting Chemical Substructures (BRICS) algorithm^68^ was employed to systematically deconstruct these molecules into smaller, synthetically viable fragments. Next, we implemented a fragment-based molecular generation framework using 3D-Monte Carlo Tree Search (MCTS) to explore the chemical space and generate geometrically optimized 3D molecules. This approach models the molecule’s structure and its interaction with the protein binding site as a Markov Decision Process (MDP), represented as *M* = (S, A, f, R). Here, S represents state space, which encompasses the molecule’s topological and conformational parameters. A is an action space (A) which consists of feasible structural modifications and f is the state transition function that defines the deterministic progression between molecular states. The molecule design process involves the strategic selection of substitution points, the integration of chemically compatible fragments, and conformational optimization to identify energetically favorable binding modes. A heuristic reward function (R) guides the stochastic exploration toward molecules that exhibit desired pharmacophoric properties. The expected reward Q (s, a) is approximated through Monte Carlo simulations, considering state-action visitation frequency and cumulative rewards. This method allows the efficient exploration of chemical space, facilitating the design of novel inhibitors with optimized interactions against WDR5-MLL1 complex targets.

## 3 Results

### 3.1 Free Energy Analysis of Ligand Interactions in the WDR5 Protein and WDR5-MLL1 Complexes

The top-screened molecules and their binding energies within the crystal structures of WDR5 protein (PDB ID: 4IA9) are presented in Table 1, while those for the WDR5-MLL1 complex (PDB ID: 4ESG) are shown in Table 2. Initially, the virtual screening methodology targeted the WDR5 using the co-crystal ligand from the 4IA9 PDB structure. To incorporate the MLL1 protein and generate the WDR5-MLL1 complex, the 4ESG structure, which includes WDR5-MLL1 motif peptide binary complex, was superimposed with 4IA9. The Glide/HTVS protocol was then employed to identify top-scoring ligands by applying a binding energy threshold of −7 kcal/mol for both the WDR5 protein and the WDR5-MLL1 complex. A total of 963,084 molecules from three libraries were screened: ChemDiv (300,528), Enamine (460,160), and Specs (202,385). Compounds were filtered through a systematic process, selecting the top 10% based on their docking scores at each step. For both WDR5 and WDR5-MLL1 complex, the initial virtual screening hits included 30,054 (ChemDiv), 46,016 (Enamine), and 20,238 (Specs) compounds, which were further refined through standard precision (SP) and extra precision (XP) scoring methods. The XP screening narrowed the hits to 300 (ChemDiv), 460 (Enamine), and 203 (Specs). Following the primary MM-GBSA screening with a cutoff of <−50 kcal/mol, the numbers were reduced significantly. For WDR5 protein, 96 compounds from ChemDiv, 145 from Enamine, and 67 from Specs were retained, while the WDR5-MLL1 complex yielded 34 hits from ChemDiv, 52 from Enamine, and 37 from Specs. Next, Lipinski’s Ro5 was applied, which further reduced WDR5 protein candidates to 59 (ChemDiv), 144 (Enamine), and 52 (Specs). The WDR5-MLL1 complex retained its previous numbers: 34 (ChemDiv), 52 (Enamine), and 37 (Specs). The distribution of molecular docking scores for both WDR5 protein and WDR5-MLL1 complex illustrated in Figure S1. Further refinement using the PAINS filter resulted in a reduction of compounds for WDR5 protein to 55 (ChemDiv), 144 (Enamine), and 48 (Specs), while the WDR5-MLL1 complex was refined to 27 (ChemDiv), 40 (Enamine), and 25 (Specs). Subsequently, compounds were filtered through binary QSAR analysis predicted therapeutic activity with a threshold of >0.5, leading to further reductions. For WDR5, this yielded 37 (ChemDiv), 94 (Enamine), and 12 (Specs), while the WDR5-MLL1 complex retained 2 (ChemDiv), 29 (Enamine), and 9 (Specs). To evaluate ligand stability, MD simulations were performed at multiple time scales with an MM-GBSA free energy cutoff of −70 kcal/mol. After three replicates of 10 ns MD/MM-GBSA, the WDR5 retained 4 (ChemDiv), and 11 (Enamine), while the WDR5-MLL1 complex yielded 2 (ChemDiv), 29 (Enamine), and 9 (Specs) compounds. At the 100 ns MD/MM-GBSA stage, WDR5 hits were reduced to 3 (ChemDiv), 9 (Enamine), and 1 (Specs), while the WDR5-MLL1 complex retained 0 (ChemDiv), 8 (Enamine), and 2 (Specs). After the final 250 ns long MD/MM-GBSA analysis, the WDR5 hits remained stable at 3 (ChemDiv), 9 (Enamine), and 1 (Specs), while the WDR5-MLL1 complex finalized with 0 (ChemDiv), 8 (Enamine), and 2 (Specs). In summary, this comprehensive multistep virtual screening and MD approach identified a small set of promising compounds targeting the WDR5 protein and WDR5-MLL1 complexes.

**Table 1:**
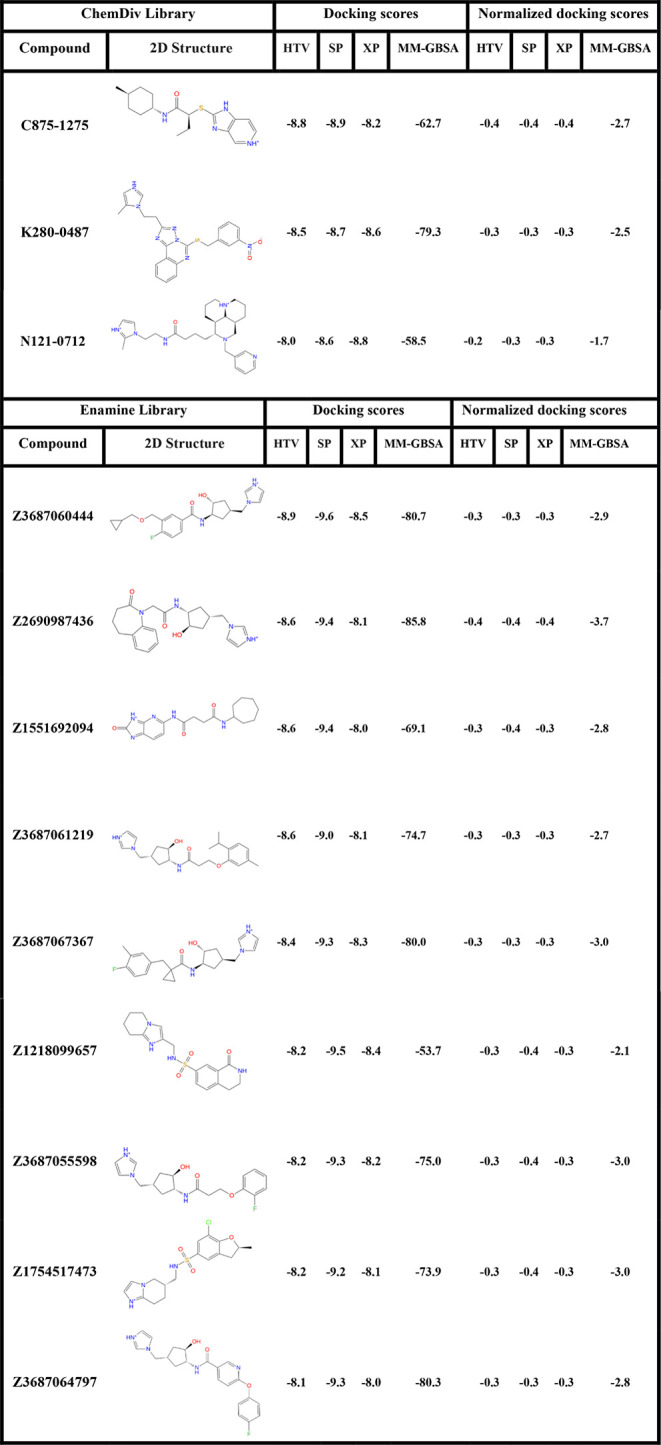
VSW Scores of Top Molecules for WDR5 and Their Docking Score Effectiveness in kcal/mol^a^

a Normalized scores are calculated as docking score per non-hydrogen atom number for each screened compound.

**Table 2:**
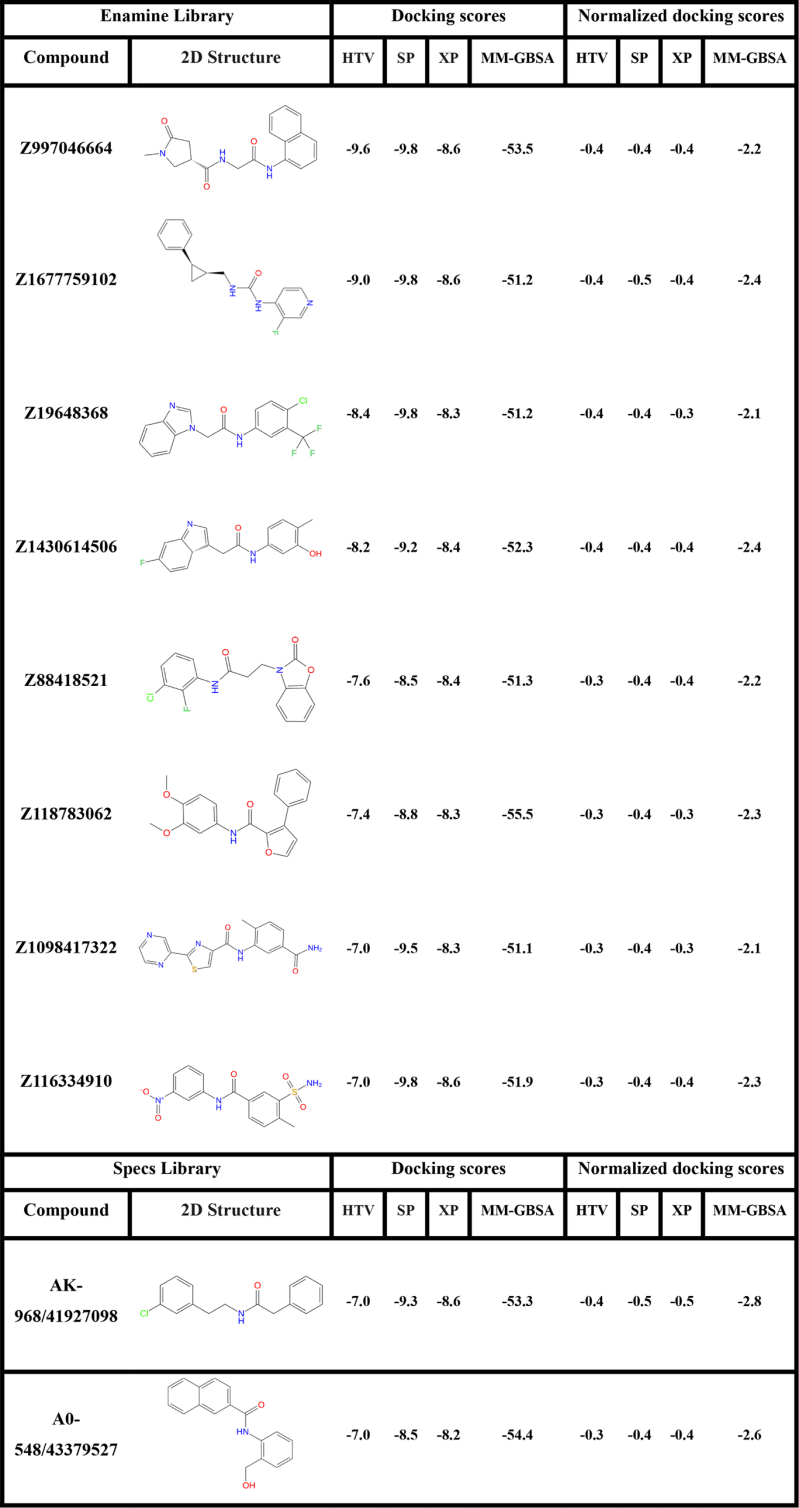
VSW Scores of Top Molecules for WDR5-MLL1 Complex and Their Docking Score Effectiveness in kcal/mol^a^

a Normalized scores are calculated as docking score per non-hydrogen atom number for each screened compound.

The virtual screening workflow comprehensively evaluated the binding potential of leading compounds derived from multiple chemical libraries against the WDR5 protein and the WDR5-MLL1 complex. The analysis involved examining VSW and MM-GBSA scores across HTVS, SP, and XP docking methods. Docking scores are analyzed by considering both the raw docking scores and the normalized (effective) docking scores (i.e., docking scores per non-hydrogen atom).

The results highlight Z2690987436 as the top-performing candidate for WDR5 protein, with MM-GBSA values of −85.8 kcal/mol (raw) and −3.7 kcal/mol (effective). Other key candidates targeting WDR5 protein included Z3687067367 (raw: −80.0 kcal/mol, effective: −3.0 kcal/mol), Z3687055598 (raw: −75.0 kcal/mol, effective: −3.0 kcal/mol), and Z3687060444 (raw: −80.7 kcal/mol, effective: −2.9 kcal/mol). In contrast, compounds such as N121–0712 displayed relatively weaker effective binding energies −58.5 kcal/mol (raw) and −1.7 kcal/mol (effective), highlighting their limited potential compared to compounds from the Enamine library.

For the WDR5-MLL1 complex, as presented in Table 2, the docking scores across HTVS, SP, and XP remained fairly close, with values ranging from −7.0 to −9.8 kcal/mol. In the MM-GBSA analysis, some compounds displayed moderate predicted effective binding affinity, such as Z118783062 (raw: −55.5 kcal/mol, effective: −2.3 kcal/mol), Z116334910 (raw: −51.9 kcal/mol, effective: −2.3 kcal/mol) and Z88418521 (raw: −51.3, effective: −2.2 kcal/mol). However, compounds from the Specs library, particularly AK-968/41927098 (raw: −53.3 kcal/mol, effective: −2.8 kcal/mol) and A0-548/43379527 (raw: −54.4 kcal/mol, effective: −2.6 kcal/mol) emerged as the one of the most promising inhibitors, given their strong MM-GBSA values and favorable binding profiles.

The interactions of these selected compounds were primarily hydrophobic, forming π-π stacking interactions with catalytic residues such PHE133, while some pocket specificity residues also exhibited hydrophilic interactions that were reported mainly with ASP107.

### 3.2 MD Simulations

#### 3.2.1 Predicted Binding Affinities of Potent Compounds at the WDR5

MD simulations were conducted to examine the structural and dynamic changes in the WDR5 and the WDR5-MLL1 complex when interacting with selected potent compounds. Simulations were performed over varying durations of 10, 100, and 250 ns. Comprehensive results for the WDR5 protein are summarized in Table S1, while the outcomes for the WDR5-MLL1 complex are represented in Table S2. The findings of WDR5 from the 250 ns MD simulations, illustrated in Figure 2, revealed that all tested compounds exhibited stronger binding affinities to WDR5 protein compared to the reference molecule IA9. Among the tested compounds, Z3687067367 demonstrated the lowest average binding energy of −78.6 kcal/mol and a low average effective MM-GBSA value of −2.9 kcal/mol. This was approximately twice as strong as IA9, which showed a moderate average binding energy of −44.5 kcal/mol, a standard deviation of 2.5 kcal/mol, and the highest effective MM-GBSA value of −1.6 kcal/mol. Other notable compounds included Z1551692094 (effective score of −2.5 kcal/mol), Z1754517473 (effective score of −2.9 kcal/mol), Z2690987436 (effective score of −3.1 kcal/mol), and Z3687064797 (effective score of −2.5 kcal/mol), all of which displayed consistently strong predicted binding affinities, with average MM-GBSA energies ranging from −72.1 to −73.6 kcal/mol and relatively low standard deviations (2.6 to 3.1 kcal/mol). The compound Z3687061219 emerged as the most consistent performer among the tested molecules, with an average MM-GBSA value of −71.2 kcal/mol, and effective MM-GBSA score of −2.5 kcal/mol, and the lowest standard deviation of 1.5 kcal/mol. Additional compounds, such as Z1218099657 (average MM-GBSA score: −63.2 kcal/mol, effective MM-GBSA score: −2.5 kcal/mol), Z3687055598 (average MM-GBSA score: −71.7 kcal/mol, effective MM-GBSA score: −2.9 kcal/mol), and Z3687060444 (average MM-GBSA score: −65.9 kcal/mol, effective MM-GBSA score: −2.4 kcal/mol), also demonstrated strong binding potentials. Lastly, compounds C875–1275, K280–0487, and N121–0712 showed improved binding affinities compared to IA9, with average MM-GBSA binding free energies ranging from −55.5 to −71.7 kcal/mol and effective MM-GBSA values between −2.0 and −2.4 kcal/mol.

**Figure 2:**
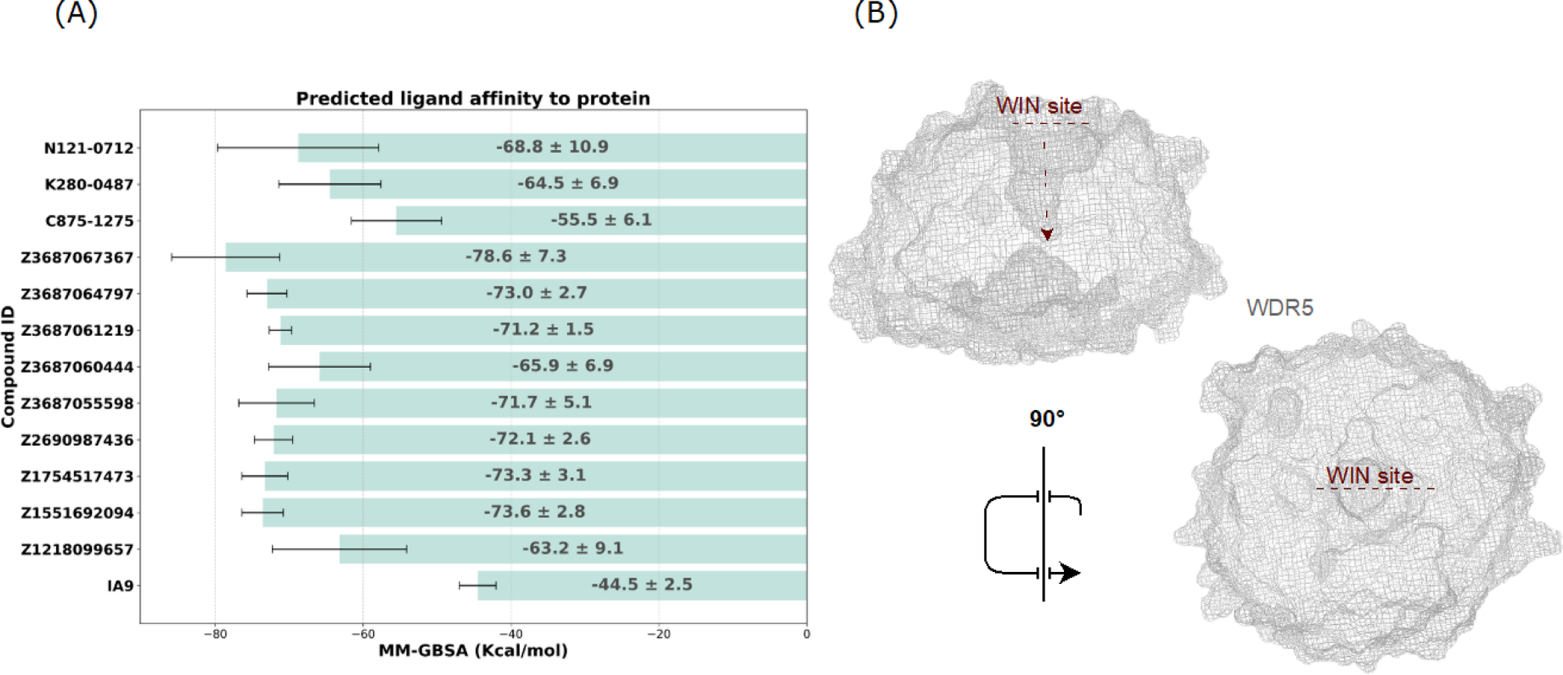
(**A**) Average MM-GBSA values of selected potent molecules targeting the WDR5 protein, derived from 250 ns MD simulations (*n* = 3). (**B**) Side and top views of mesh representation of the WDR5 protein structure, highlighting the WIN site from two different orientations.

#### 3.2.2 Predicted Binding Affinities of Potent Compounds at the WDR5-MLL1 Complex

Figure 3 provides a comprehensive analysis of ligand binding affinities and their impact on the WDR5-MLL1 complex interaction. The left panel (A) illustrates the predicted binding affinities of selected ligands to the WDR5-MLL1 complex, while the right panel (B) focuses on the influence of these ligands on WDR5-MLL1 interaction (PPI) stability. Together, these analyses offer insights into the dual roles of the ligands: their ability to bind effectively and disrupt the critical WDR5-MLL1 complex interaction. The left panel (A) reveals that the reference molecule, IA9, has an average MM-GBSA score of −75.2 kcal/mol, serving as the baseline for evaluating other ligands. Among the tested compounds, Z116334910 (−77.3 kcal/mol), Z19648368 (−80.6 kcal/mol), Z1430614506 (−82.8 kcal/mol), and Z88418521 (−89.3 kcal/mol) demonstrated stronger binding affinities, indicating their superior potential to inhibit WDR5-MLL1 complex through direct binding. In contrast, ligands such as Z1677759102 (−67.8 kcal/mol), Z1098417322 (−70.6 kcal/mol), Z997046664 (−72.5 kcal/mol), and Z118783062 (−74.7 kcal/mol) exhibited slightly weaker binding affinities compared to IA9. While these ligands may appear less potent based on binding affinity alone, their effects on PPI disruption suggest that they could act as effective modulators, potentially through mechanisms such as allosteric modulation or interaction specificity.

**Figure 3:**
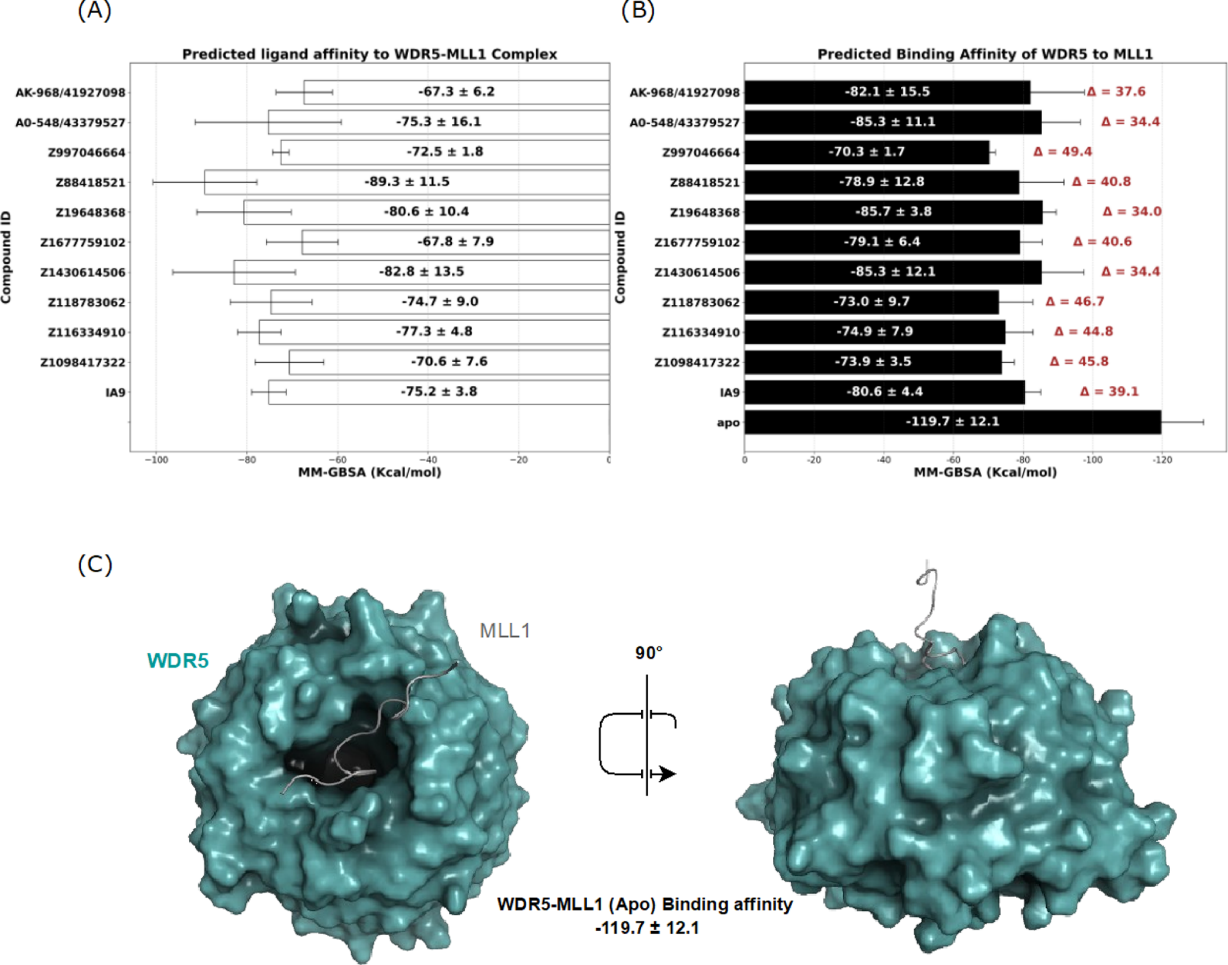
Average MM-GBSA values of selected potent molecules targeting the WDR5-MLL1 complex interaction illustrated in left white bars Figure (A) and average MM-GBSA values between WDR5 protein and MLL1 in right black bars Figure (B), derived from 250 ns MD simulations (*n* = 3). (C) The interface complex model of WDR5-MLL1 complex (in surface representation) in two different orientations.

#### 3.2.3 Predicted Binding Affinities of WDR5 with MLL1 in the Presence of Potent Hit Compounds

The right panel (B) shifts in Figure 3, focus to the disruption of the WDR5-MLL1 complex interaction, assessed by calculating the difference in binding energy between the unliganded (apo) and ligand-bound states. The WDR5-MLL1 complex, in its apo form, exhibited an average interaction energy (MM-GBSA) of −119.7 kcal/mol, representing a strong and stable PPI. Positive control compound IA9, when bound to the complex, reduced this interaction energy to −80.6 kcal/mol, demonstrating its capability to destabilize the WDR5-MLL1 complex interaction. Other ligands showed varied effects: Z1677759102 (−79.1 kcal/mol) and Z88418521 (−78.9 kcal/mol) exhibited moderate disruption, slightly exceeding IA9’s destabilizing effect. Meanwhile, Z116334910 (−74.9 kcal/mol), Z1098417322 (−73.9 kcal/mol), Z118783062 (−73.0 kcal/mol), and Z997046664 (−70.3 kcal/mol) showed further destabilization, and emerged as the most effective disruptors. These results highlight their potential to significantly weaken the WDR5-MLL1 complex interaction. The effective (normalized) MM-GBSA scores further support these observations, providing additional context for ligand performance. Z88418521, having the lowest effective MM-GBSA score (−3.9 kcal/mol), demonstrated the strongest binding affinity, highlighting its potential as a high-affinity binder capable of substantially altering the WDR5-MLL1 complex interaction. Other ligands, such as Z997046664 and Z118783062, showed moderate binding efficiencies but excelled in destabilizing PPIs, underscoring the diversity of mechanisms among the tested compounds.

Overall, Figure 3 underscores the multifaceted nature of ligand interactions with the WDR5-MLL1 complex. Compounds such as Z116334910 and Z88418521 appear to show promising binding affinities and may help disrupt WDR5-MLL1 complex interaction, suggesting they are candidates worth considering for further investigation.

### 3.3 3D-MCTS Compounds Binding: Impact of Free Energy at WDR5 Protein and WDR5-MLL1 Complexes

The 3D-MCTS approach was used to generate molecules targeting the WDR5 and the WDR5-MLL1 complex. A total of 21,692 new molecules were derived and screened for binding to the WDR5, while 9,127 molecules were derived and screened for the WDR5-MLL1 complex. The initial docking-based screening using an XP docking score threshold of −7 kcal/mol resulted in 725 molecules for the WDR5-MLL1 complex and 577 molecules for the WDR5 protein. Subsequent filtering based on Lipinski’s Ro5 criteria further reduced the number of compounds to 200 for the WDR5-MLL1 complex and 172 for the WDR5. Additional filters, including PAINS analysis and binary QSAR analysis (i.e., normalized therapeutic activity score >0.5), further refined the candidate molecules to 128 for the WDR5 protein and 130 for the WDR5-MLL1 complex. For the WDR5 protein, the binding affinity of the remaining compounds, as assessed by 10 ns (3 replicates) of MD simulations and MM-GBSA analysis, did not meet the criterion of a binding free energy more favorable than −70 kcal/mol as shown in Figure S2. Therefore, no further simulations were performed on these molecules for targeting the WDR5 protein. In contrast, for the WDR5-MLL1 complex, the 130 compounds from the initial screening were subjected to MD simulations (10 ns, 3 replicates), with an average MM-GBSA binding free energy cutoff of −70 kcal/mol shown in Figure S3A. This step reduced the number of candidates to 25 for 100 ns MD simulations (Figure S3B). Finally, 250 ns of MD simulations was performed on 7 of these selected molecules (Tables S3). The extended 250 ns MD simulations study revealed that five new generated molecules met the binding energy threshold of −70 kcal/mol. While original molecules like Z88418521 (−89.3 kcal/mol), Z19648368 (−80.6 kcal/mol), and Z1463041506 (−82.8 kcal/mol) demonstrated superior affinity to the WDR5-MLL1 complex, the calculated interaction energies from 250 ns simulations showed varied results (Tables S2). Notably, since several original molecules showed promising energy disruption between WDR5 protein and MLL1 complex compared to the newly generated molecules, including Z997046664 (−70.3 kcal/mol), Z118783062 (−73.0 kcal/mol), Z116334910 (−74.9 kcal/mol), and Z88418521 (−78.9 kcal/mol). Consequently, no further analysis was performed on these new generated analog compounds.

### 3.4 Post-MD Simulations Analysis

#### 3.4.1 sMD Simulations Reveal Stable Ligand Binding in WDR5-MLL1 Complex versus WDR5 Protein Alone

Figure 4 represents a comparative analysis of the unbinding force profiles derived from sMD simulations conducted at two distinct pulling velocities (0.1 Å/ps and 0.01 Å/ps) to interrogate the mechanical dissociation behavior of selected ligand-WDR5 complexes. The force, is plotted during the simulation time (ps), offering quantitative insight into the mechanical stability and retention dynamics of each ligand within the WDR5 binding pocket.

**Figure 4:**
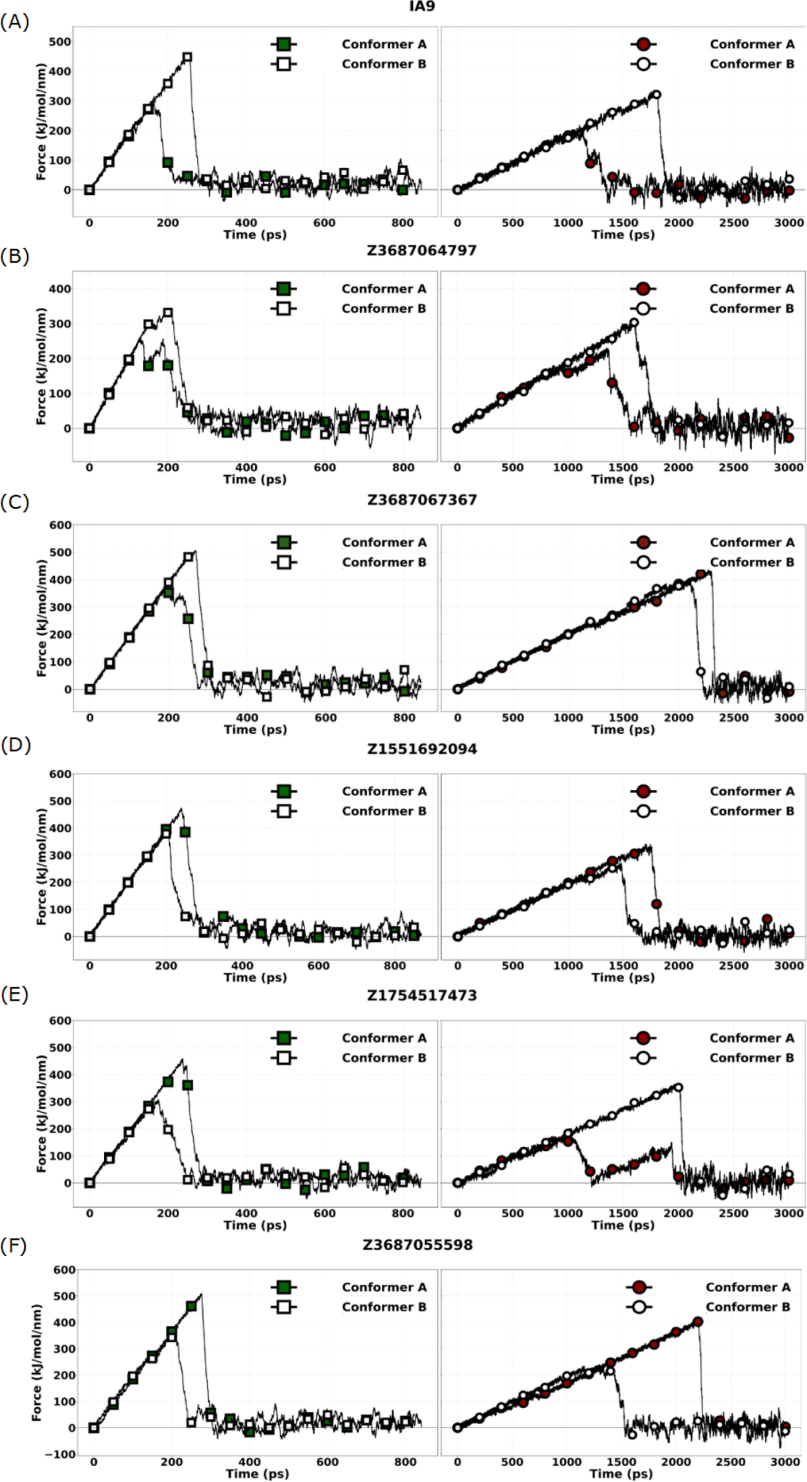
sMD force profiles for two protein conformers (A and B) in complex with different ligands (A–F). Conformer A represents the protein structure (WDR5) with the lowest RMSD to the average structure, while Conformer B corresponds to the second lowest RMSD structure. Each subpanel displays the force (kJ/mol/nm) versus time (ps) profiles under two different pulling speeds: 0.01 Å/ps (left) and 0.1 Å/ps (right). Ligands tested are (A) IA9 (reference compound); (B) Z3687064797; (C) Z3687067367; (D) Z1551692094; (E) Z1754517473; and (F) Z3687055598.

As anticipated, the slower pulling speed (0.01 Å/ps) resulted in substantially prolonged unbinding durations and reduced peak dissociation forces across all compounds tested, consistent with the enhanced capacity of the system to undergo conformational relaxation during the dissociation process. This inverse relationship between pulling velocity and observed unbinding force underscores the kinetic sensitivity of force-probe simulations and reinforces the relevance of low-velocity sMD conditions for approximating biophysically realistic unbinding events.

The reference ligand IA9 revealed notable conformational differences under mechanical stress. Conformer A exhibited moderate mechanical resilience, characterized by a dissociation force of 280 kJ/mol/nm at 0.1 Å/ps and a prolonged residence time of 1300 ps at 0.01 Å/ps, associated with a reduced dissociation force of 200 kJ/mol/nm. In contrast, Conformer B displayed consistently superior mechanical stability, withstanding significantly higher rupture forces (480 kJ/mol/nm at 0.1 Å/ps and 320 kJ/mol/nm at 0.01 Å/ps) and longer binding durations at both pulling speeds. These observations suggest that conformer B engages in a more stabilized and energetically favorable interaction mode with the WDR5 binding interface.

Among the evaluated hits, Z3687055598 Conformer A exhibited the most pronounced mechanical robustness, maintaining exceptionally high dissociation forces at both pulling speeds (500 kJ/mol/nm at 0.1 Å/ps and 400 kJ/mol/nm at 0.01 Å/ps), coupled with the longest binding durations recorded across the ligand set (2550 ps at the slower pulling velocity). This behavior indicates a highly persistent binding orientation with minimal force-induced destabilization, rendering this compound a compelling candidate for further experimental validation.

Compound Z3687067367 displayed marked conformational divergence. While Conformer B exhibited the highest rupture force at the faster pulling speed (500 kJ/mol/nm), Conformer A demonstrated enhanced resilience at the slower speed, maintaining a dissociation force of 400 kJ/mol/nm and a residence time exceeding 2500 ps. This differential response suggests that each conformer may exploit distinct anchoring interactions or escape pathways under mechanical perturbation.

Interestingly, Z1754517473 exhibited a reversal in conformational preference depending on the pulling velocity. Conformer A, while exhibiting robust dissociation characteristics at 0.1 Å/ps (480 kJ/mol/nm), displayed markedly diminished stability at 0.01 Å/ps (180 kJ/mol/nm), indicative of kinetic trapping that does not persist under relaxed conditions. Conversely, Conformer B demonstrated improved performance at the lower velocity, suggesting a more thermodynamically favorable binding conformation under near-equilibrium conditions.

Conformer A of Z1551692094 maintained consistent and strong binding stability across both pulling regimes, with dissociation forces of 480 kJ/mol/nm and 320 kJ/mol/nm, respectively. Conformer B of the same molecule, however, showed a reduction in both force and duration, reflecting a less favorable interaction geometry.

Compound Z3687064797 exhibited moderate stability, with Conformer B displaying improved binding retention at both pulling velocities relative to Conformer A, though neither conformer matched the top-performing ligands in absolute force or duration metrics.

Taken together, these data reveal significant ligand- and conformer-dependent variability in mechanical stability, highlighting the critical influence of both pulling velocity and conformational state on dissociation behavior. Importantly, several ligands, particularly Z3687055598 and Z3687067367, demonstrated force-resilient binding profiles across both velocity regimes, suggesting their structural compatibility with the WDR5 binding pocket and robustness under mechanical stress. These findings underscore the value of sMD simulations not only for ranking binding affinity proxies but also for elucidating mechanistically relevant dissociation trajectories that can inform ligand prioritization and downstream optimization.

Figure 5 represents the results of sMD simulations conducted on the WDR5–MLL1 complex to evaluate the mechanical dissociation behavior of selected ligands under two pulling regimes (0.1 Å/ps and 0.01 Å/ps). The dissociation force and unbinding duration were used as surrogate indicators of binding strength and residence time within the MLL1-binding cavity of WDR5.

**Figure 5:**
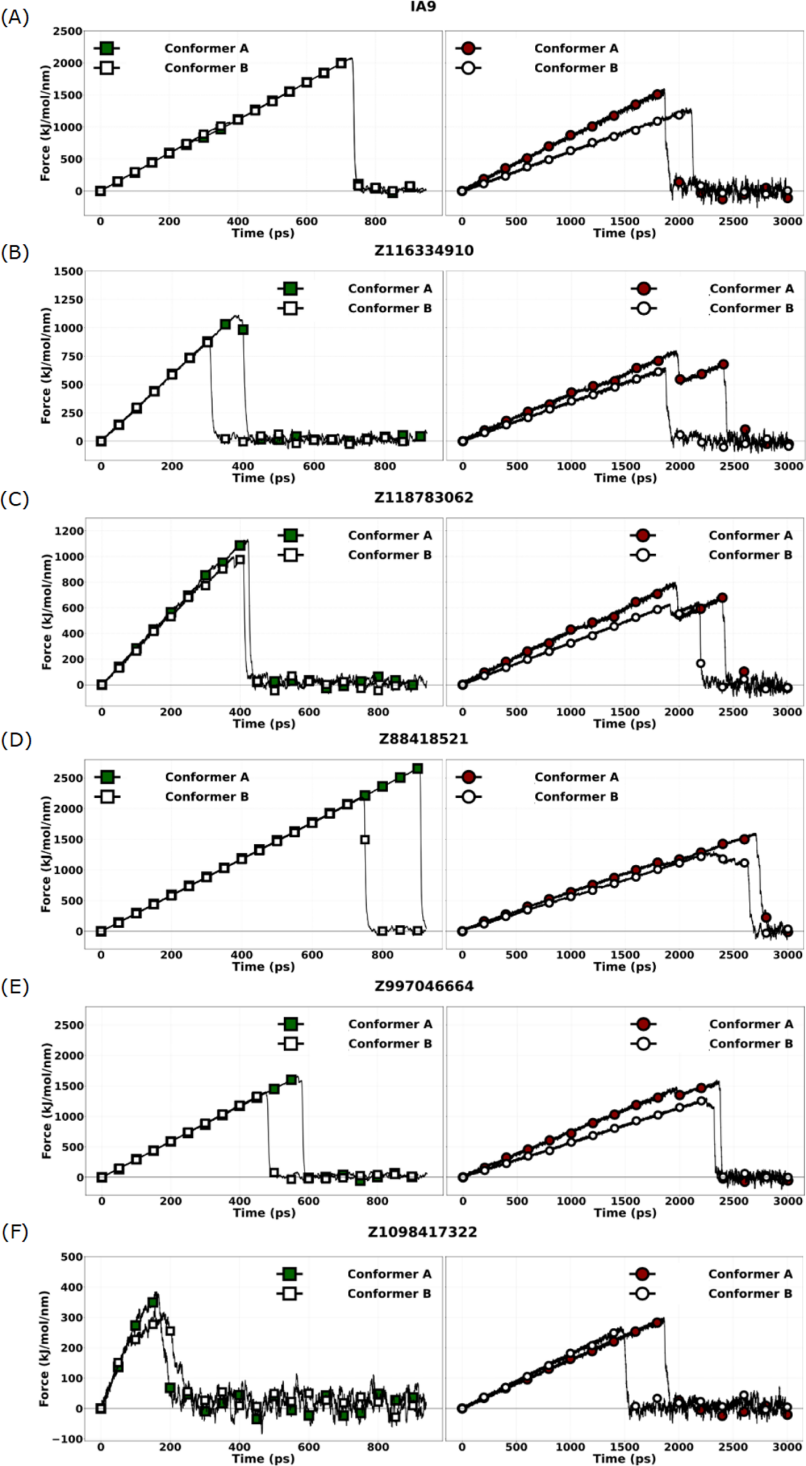
sMD force profiles for two protein conformers (A, B) in complex with different ligands (A–F). Conformer A represents the protein structure (WDR5-MLL1 complex) with the lowest RMSD to the average structure, while Conformer B corresponds to the second lowest RMSD structure. Each subpanel displays the force (kJ/mol/nm) versus time (ps) profiles under two different pulling speeds: 0.01 Å/ps (left) and 0.1 Å/ps (right). Ligands tested are (A) IA9 (reference compound); (B) Z116334910; (C) Z118783062; (D) Z88418521; (E) Z997046664; and (F) Z1098417322.

The reference compound IA9 demonstrated the expected velocity-dependent behavior. Conformer A exhibited moderate interaction stability, dissociating at 280 kJ/mol/nm within 220 ps at high pulling speed. At 0.01 Å/ps, the unbinding duration increased to 1300 ps with a reduced dissociation force of 200 kJ/mol/nm, confirming the typical inverse correlation between rupture force and pulling duration. Conformer B, however, consistently outperformed its counterpart, withstanding higher dissociation forces (480 kJ/mol/nm at 0.1 Å/ps and 320 kJ/mol/nm at 0.01 Å/ps) and exhibiting a significantly extended residence time of 2000 ps at slower velocity. These data suggest a more favorable and mechanically robust binding orientation for Conformer B under both force regimes.

Among the tested hits, Conformer A of Z3687055598 demonstrated exceptional force resilience across all conditions. At 0.1 Å/ps, it resisted dissociation until 300 ps with a rupture threshold of 500 kJ/mol/nm. Under slower pulling, it remained bound for 2550 ps and dissociated at 400 kJ/mol/nm, making it one of the most mechanically persistent ligands in the series. In contrast, Conformer B of the same compound exhibited markedly reduced retention and force resistance across both velocities, highlighting the substantial conformational influence on binding robustness.

Z3687067367 exhibited a distinct conformer-dependent inversion. While Conformer A showed modest performance at high velocity (220 ps, 330 kJ/mol/nm), it displayed superior mechanical stability under slower force application, maintaining its position for over 2500 ps and requiring 400 kJ/mol/nm for dissociation. Conversely, Conformer B exhibited peak performance under high velocity (300 ps, 500 kJ/mol/nm) and sustained significant force resistance at low velocity (2300 ps, 380 kJ/mol/nm), indicating that both conformers engage the WDR5-MLL1 interface via complementary and robust anchoring geometries.

Z1754517473 revealed pronounced velocity-dependent conformational dynamics. Conformer A demonstrated strong mechanical retention under high force (280 ps, 480 kJ/mol/nm), but substantially weakened at lower pulling speed (1200 ps, 180 kJ/mol/nm), suggesting kinetic stabilization that does not persist under near-equilibrium conditions. In contrast, Conformer B exhibited a reversed trend, improving markedly at 0.01 Å/ps (2100 ps, 380 kJ/mol/nm) despite reduced performance under rapid pulling. This divergence underscores the critical influence of conformational adaptability on binding persistence.

Conformer A of Z1551692094 maintained a high-performance profile across both velocities, dissociating at 480 kJ/mol/nm (300 ps) under rapid pulling and sustaining prolonged binding at 0.01 Å/ps (1800 ps, 320 kJ/mol/nm). Conformer B displayed slightly reduced performance across both metrics but remained within a favorable range, suggesting a consistent, albeit slightly weaker, interaction geometry.

Z3687064797 showed modest yet consistent binding across conformers. Conformer A demonstrated intermediate resilience (280 ps, 250 kJ/mol/nm at 0.1 Å/ps; 1550 ps, 220 kJ/mol/nm at 0.01 Å/ps), while Conformer B offered moderate improvements in both unbinding duration and rupture force.

A comparative assessment of peak dissociation forces across all compounds revealed velocity-dependent shifts in binding rankings. At the faster pulling speed (0.1 Å/ps), Conformer A of Z3687055598 and Conformer B of Z3687067367 exhibited the strongest interactions (both 500 kJ/mol/nm), followed closely by Conformer A of Z1551692094, Conformer A of Z1754517473, and Conformer B of IA9 (all approximately 480 kJ/mol/nm). However, under slower velocity conditions (0.01 Å/ps), the relative order was altered. While Conformer A of Z3687055598 retained its leading position (400 kJ/mol/nm), it was now matched by Conformer A of Z3687067367, whereas Conformer A of Z1754517473 dropped significantly in both dissociation force and retention time.

These findings underscore the importance of simulating mechanical stability across multiple force regimes, as some ligand conformers exhibit consistent high-affinity binding regardless of pulling speed, while others demonstrate force-dependent conformational preferences. This variability suggests that optimal ligand engagement with the WDR5-MLL1 interface is not solely determined by static affinity, but is instead governed by a dynamic balance between structural adaptability and mechanical resilience.

#### 3.4.2 MD Analysis of WDR5 Inhibitors: RMSD and Root Mean Square Fluctuation (RMSF) Comparisons with Reference Molecule IA9

In the current study, the trajectories of potent selected five ligands with the best binding energy besides the reference molecule IA9 (Figure S4) were obtained to be investigated. Frame-by-frame conformational fluctuation of WDR5-ligand complex were captured to compute the average distance of simulated complex. Therefore, RMSD values for WDR5-ligand complex backbone were extrapolated from each 250 ns simulation trajectories. The mean RMSD values for all 250 ns simulations initially averaged around 0.8 Å. Detailed backbone RMSD analysis revealed a common pattern of initial structural deviation within the first 50 ns across all molecules. However, by the later stages of the simulation, all molecules converged to a stable structural conformation, with RMSD values ranging between 1.2 and 1.6 Å. Notably, none of the molecules exceeded an RMSD of 2 Å, which strongly suggests maintained structural integrity and conformational stability during the entire MD simulation. RMSF analysis of carbon alpha atoms revealed similar molecular behavior across all studied structures. The terminal regions near residues 30 and 334 consistently displayed high flexibility (RMSF > 4 Å), while the overall molecular flexibility pattern remained remarkably stable. No significant local variations or distinguishing characteristics were observed between the molecules, indicating structural and dynamic equivalence.

#### 3.4.3 MD Analysis of WDR5-MLL1 Complex Inhibitors: RMSD and RMSF Comparisons with Reference Molecule IA9

For the WDR5-MLL1 complex, the results revealed distinct patterns in backbone stability and residue flexibility among the analyzed molecules. In Figure S5, RMSD analysis of backbone atoms showed IA9, the reference molecule, exhibiting the highest fluctuations besides Z1098417322 (3.5–4 Å), indicating greater overall structural instability compared to the other molecules. However, despite these notable fluctuations, IA9 remained within a stable range, not exceeding 4.5 Å. In contrast, Z116334910 demonstrated the most stable RMSD profile (2–2.5 Å), suggesting a rigid conformation throughout the simulation. The remaining molecules (Z88418521, Z118783062, and Z997046664) displayed intermediate RMSD values (2.5–3 Å) with varying degrees of fluctuation. Notably, Z118783062 showed an interesting transition from initial stability to higher RMSD values after 50 ns. RMSF analysis of carbon alpha atoms revealed remarkably consistent flexibility profiles across all molecules, including IA9. Key features included a prominent high peak around residue 25, generally low RMSF for most residues. This consistency in RMSF profiles, despite differences in RMSD, suggests that while global conformational stability varies among the molecules, local structural dynamics are largely conserved. These findings indicate that modifications in these molecules primarily affect overall structural stability rather than local flexibility patterns. This insight could be valuable for understanding how structural changes impact function and for guiding future design of molecules with desired stability profiles while maintaining essential local dynamic properties.

#### 3.4.4 Protein Flexibility Analysis Reveals Changes in Side Chain Dynamics in WDR5 Protein Ligand Binding

The WDR5-ligand interactions observed across six distinct ligands (IA9, Z23687067367, Z1551692094, Z1754517473, Z3687055598, and Z3687064797) reveal a consistent pattern of structural dynamics. In each case, the protein maintains a rigid backbone, with α carbon displacements generally below 1 Å, while exhibiting significant flexibility in the side chains. This flexibility is evidenced by side chain displacements ranging from 1.5 to 2.2 Å in key residues interacting with the ligand. Aromatic residues (e.g., TYR and PHE) and hydrophobic residues (e.g., LEU, ILE, and VAL) frequently show pronounced movements, highlighting their critical role in ligand accommodation through mechanisms such as π-stacking, hydrophobic interactions, and induced fit. Additionally, charged residues (e.g., ASP, GLU, and LYS) demonstrate considerable mobility, suggesting the involvement of electrostatic interactions.

The repeated engagement of residues like TYR131, PHE149, and LEU224/234 across multiple ligand interactions underscores their importance in the binding pocket. A comparative analysis of protein residue displacements with IA9 in Figure 6, specifically focusing on α carbon in green and side chain movements in red, shows that the *x*-axis represents the individual residues in contact with IA9, while the *y*-axis quantifies the RMSF in (Å). Noteworthy side chain displacements include TYR131 (∼2.2 Å), PHE149 and ILE263 (both ∼2.1 Å), as well as TYR191, and LYS259, each exhibiting movements exceeding 1.75 Å. These substantial side chain fluctuations indicate involvement in ligand binding or reflect inherent flexibility that facilitates conformational adjustments to accommodate ligand binding.

**Figure 6:**
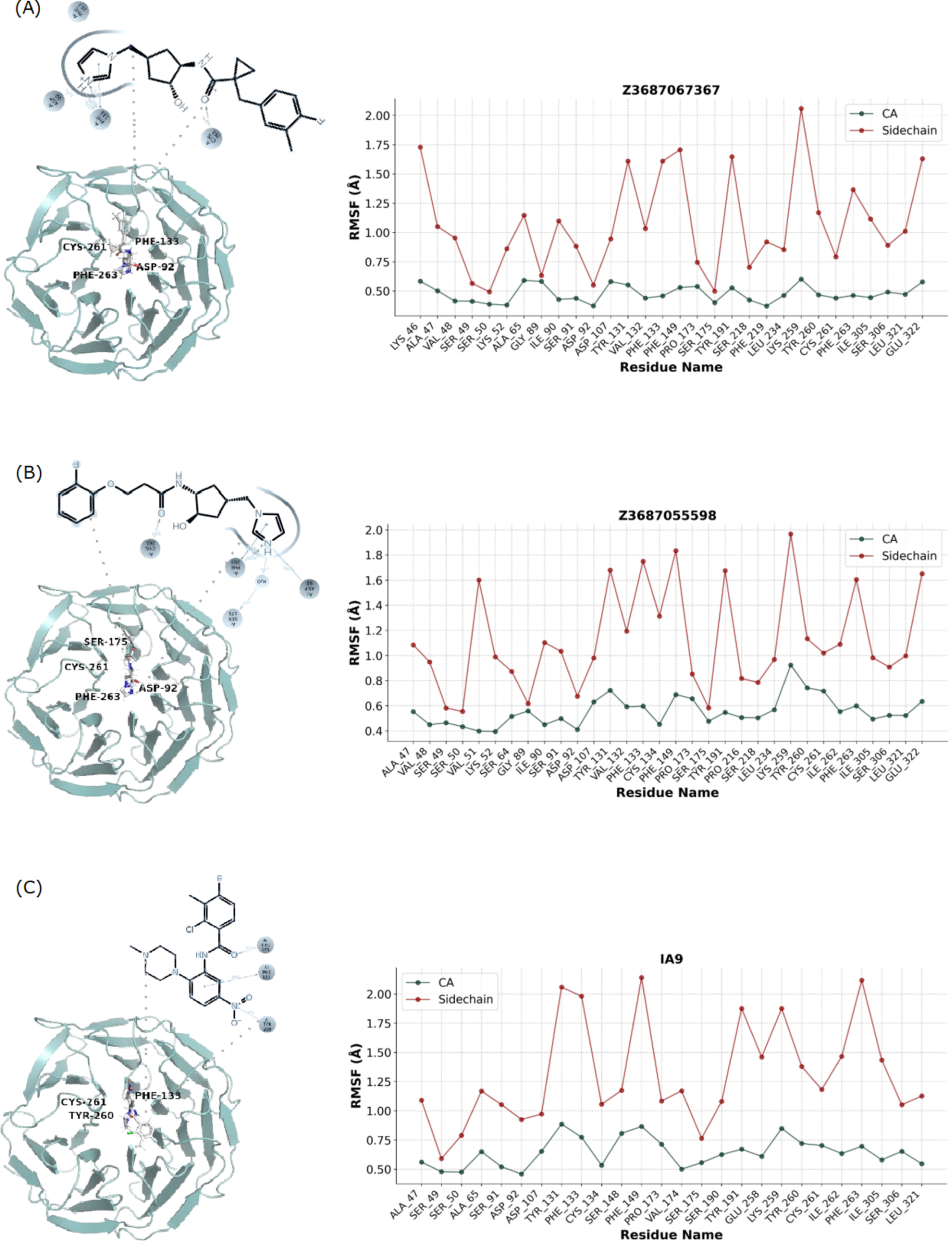
WDR5 protein's conformational adjustments in its CA (α carbon) and side chain positions while interacting with the molecules (A) Z23687067367, (B) Z3687055598, and (C) the reference molecule (PDB: IA9) during 250 ns of simulations (*n* = 3).

In the case of Z23687067367 as shown in Figure 6A, residues such as LYS46, TYR131, PHE133, PHE149, TYR191, LYS259, and GLU322 exhibit side chain RMSF surpassing 1.5 Å. Conversely, the CA displacements remain mostly below 0.6 Å, emphasizing the backbone’s rigidity. Residues such as TYR131, PHE149, and LYS259 show significant side chain mobility due to their active roles in ligand binding, allowing them to engage in hydrogen bonds, hydrophobic contacts, and ionic interactions, while adjusting their positions to enhance ligand stability. The analysis of protein residues in relation to Z3687055598 in Figure 6B highlights peaks in side chain RMSF for residues like VAL51 (∼1.6 Å), TYR131 (∼1.7 Å), VAL132 (∼1.2 Å), PHE 133 (∼1.8 Å), CYS134 (∼1.3 Å), PHE149 (∼1.8 Å), TYR191 (∼1.7 Å), LYS259 (highest ∼ 2.0 Å), PHE263 (∼1.6 Å) and GLU322 (∼1.6 Å).

For Z1551692094 in Figure S6, side chain displacements are prominent in residues like TYR260 (peak ∼ 2.1 Å), PHE149, PRO173, and GLU322, all exceeding 1.5 Å. Other residues, including TYR131, CYS134, and LEU224, also display notable side chain motion. The clear distinction between CA and side chain displacements reflects the protein’s ability to maintain overall structural integrity while enabling local flexibility in side chain movements. Similarly, in Figure S7 Z1754517473, notable displacements occur in residues such as LEU224 (∼2.1 Å), PHE149, TYR191, and GLU322 (all >1.5 Å), with additional residues like ASP107, VAL132, and ILE305 showing considerable motion. Lastly, the analysis of protein residues in relation to Z3687064797 Figure S8, illustrates similar trends, with significant side chain displacements in ASP107 (∼1.6 Å), VAL132 (∼1.5 Å), CYS134 (∼1.5 Å), LEU234 (highest ∼ 2.1 Å), and ILE305 (∼1.7 Å).

#### 3.4.5 WDR5-MLL1 Complex Flexibility Analysis Reveals Changes in Side Chain Dynamics in Complex Ligand Binding

The analysis of ligand-induced conformational changes in the WDR5 and MLL1 complexes upon binding to various ligands reveals distinct patterns of structural shifts as well, with specific residues displaying substantial displacements. These findings shed light on the dynamic behavior of protein–ligand interactions and highlight the roles of MLL1 and WDR5 protein complex in stabilizing these complexes. For instance, Figure 7, the IA9 ligand interacts with residues from both MLL1 protein and WDR5 protein, causing significant displacements, especially in LEU3770, ALA47, and TYR131, with shifts exceeding 2 Å. LEU3770, experiences a particularly notable displacement, nearly 6 Å, underscoring the adaptability of these residues in accommodating the ligand. HIS3761, and LEU3770 exhibit flexibility, potentially contributing to ligand stabilization while preserving the structural integrity of the complex. In contrast Figure 7A, the Z88418521 molecule predominantly affects the MLL1 protein, leading to pronounced fluctuations in the PRO3756-PRO3760 region, with movements surpassing 5 Å for both CA and side chain atoms. The extreme displacement of PRO3756 (∼9 Å) and a notable peak at LEU3770 (∼4.5 Å) indicate significant flexibility in these regions, suggesting substantial structural rearrangements in MLL1, with WDR5 protein playing a more minor role in the interaction. The Z116334910 molecule in Figure 7B induces significant conformational changes in MLL1, particularly. The graph shows large displacements (up to 8 Å) for both CA and side chain atoms in MLL1 protein residues GLU3755 and ASN3759, followed by a sharp decline in displacement values up to ALA3764. This suggests substantial restructuring in this region of MLL1 protein upon ligand binding. Beyond ALA3764, displacements stabilize and remain generally below 2 Å, indicating a return to a more stable protein conformation. Side chain atoms typically show a slight displacement to CA atoms, especially in the early residues, reflecting their greater flexibility. While not as pronounced as in some examples, MLL1 protein residues like PRO3760 and GLY3762 still exhibit notable displacements. Overall, the data indicates that Z116334910 primarily affects a specific region of MLL1 protein and WDR5 protein, while the rest of the complex structure maintains relative stability. This pattern of localized conformational change in MLL1 protein could be crucial for understanding the molecule’s mechanism of action or its impact on MLL1 protein function.

**Figure 7:**
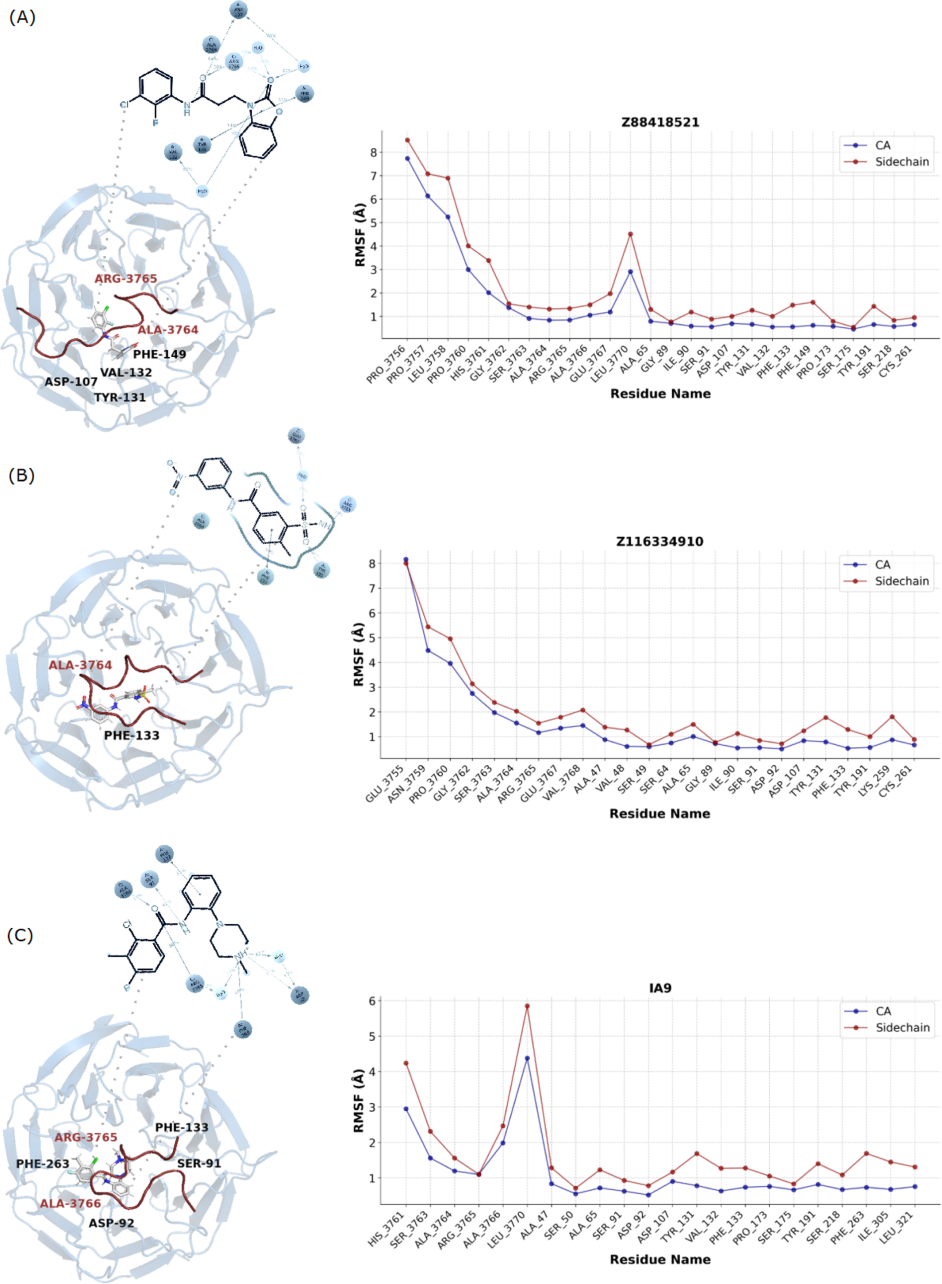
WDR5-MLL1 complex’s conformational adjustments in its CA and side chain positions while interacting with the molecules (A) Z88418521, (B) Z116334910, and (C) the reference molecule (PDB: IA9) during 250 ns of simulations (*n* = 3).

Similarly, Figure S9 shows the Z118783062 molecule primarily engages the MLL1 protein, inducing extreme displacements exceeding 9 Å in PRO3756 and LEU3758 for both CA and side chain atoms. Another prominent peak at LEU3770 (∼5 Å) supports the fact of considerable conformational changes in MLL1. Residues between GLY3762 and VAL3768 exhibit a gradient of decreasing movement, stabilizing farther from the ligand, while WDR5 protein undergoes only minor adjustments. On the other hand, the Z997046664 molecule in Figure S10 induces major conformational changes primarily in MLL1, with GLU3755 and PRO3757 showing displacements exceeding 6 Å for both CA and side chain atoms. Another significant peak at LEU3770 (5.7 Å for side chain, 4.3 Å for CA) highlights the extensive rearrangements in this region. The progressive stabilization of residues between HIS3761 and VAL3768 contrasts with the relative stability of WDR5 protein, which remains largely unaffected by the ligand.

Besides, the Z1098417322 molecule induces notable displacements in MLL1 protein as illustrated in Figure S11, particularly in LEU3758 (6.1 Å for side chain, 4.8 Å for CA) and GLY3762, suggesting significant restructuring in this region. Beyond ALA3765, displacements diminish, indicating a return to a more stable protein conformation. WDR5 protein remains relatively stable across all ligand interactions, showing only minor conformational changes. In summary, MLL1 protein undergoes significant conformational rearrangements upon ligand binding, particularly in regions around LEU3770, while WDR5 protein generally exhibits greater structural stability, contributing less to the ligand interaction across the various ligands.

#### 3.4.6 Interaction Patterns of WDR5 Protein and WDR5-MLL1 Complex with Diverse Ligands

The interactions between WDR5 protein and various ligands revealed complex molecular binding patterns, with PHE133, CYS261 and PHE263 emerging as critical residue in most interactions. Moreover, some ligands demonstrated unique binding characteristics that highlighted the protein’s interaction potential. For instance, the IA9 ligand primarily engaged with the protein through hydrophobic interactions with PHE133 and water-mediated hydrogen bonding with CYS261­(Figure S12). Furthermore, subsequent ligands displayed increasingly sophisticated interaction profiles. In particular, Z3687067367 exhibited a complex binding mode, involving hydrogen bonding and ionic interactions with ASP92, combined with hydrogen and hydrophobic interactions with PHE133. Additionally, it formed hydrogen bonds, hydrophobic interactions, and water-bridged connections with CYS261 and PHE263 (Figure S13). Correspondingly, Z1551692094 established hydrogen bonds with ILE90 and SER91, while presenting multifaceted interactions with PHE133 through hydrogen bonding, hydrophobic contacts, and water bridge formations. Notably, hydrophobic interactions with PHE263 were also observed (Figure S14). Similarly, Z1754517473 demonstrated interactions via hydrogen and ionic bonds with ASP92, hydrophobic interactions with PHE133, hydrogen and ionic bonds with CYS261, and hydrophobic interactions with PHE263 (Figure S15). Likewise, Z3687055598 showed diverse interactions, including hydrogen and ionic bonds with ASP92, hydrophobic interactions with PHE133, hydrogen water bridge bonds with CYS261, and hydrophobic interactions with PHE263 (Figure S16). Finally, Z3687064797 engaged through hydrogen and hydrophobic interactions with PHE133, hydrophobic interactions with TYR191, and hydrogen water bridge bonds with CYS261 (Figure S17).

The molecular interactions involving WDR5-MLL1 complex, and different ligands demonstrated complex binding configurations. Notably, the amino acid PHE133 again appeared to play a pivotal role in the majority of the WDR5-MLL1 complex interactions, Moreover, In Figure S18, the interactions between WDR5-MLL1 complex with the reference molecule IA9 highlighted the molecular complexity. WDR5 protein demonstrated hydrogen bonding with ALA91, ionic-water bridges with ASP92, and hydrophobic interactions with PHE133 and PHE263. Concurrently, MLL1 protein showed interaction through hydrogen-water bridges with ARG3765. Furthermore, Figure S19 revealed WDR5 protein’s interactions with Z88418521, characterized by hydrogen and water bridges with ASP107 and hydrophobic interactions involving PHE133 and PHE149. Similarly, MLL1 protein demonstrated interactions through hydrophobic contacts with ALA3764 and hydrogen-water bridges with ARG3765. In Figure S20, the interactions between Z116334910 and WDR5 protein were marked by hydrophobic interactions with PHE133 and hydrogen bonding with TYR191. Correspondingly, MLL1 protein exhibited interactions through hydrophobic interactions and water bridges with ALA3764, and hydrogen bonding and water bridges with ARG3765 and GLU3767. Figure S21 illustrated the interactions between Z118783062 and WDR5 protein through water bridge interactions with ASP107 and hydrophobic interactions with PHE133. Meanwhile, MLL1 protein demonstrated interactions through a combination of hydrogen bonding and hydrophobic interactions with ARG3765. Similarly, Figure S22 showed the interactions between Z997046664 and WDR5 protein via hydrogen bond and water bridges with ASP107 and hydrophobic interactions with PHE133. In contrast, MLL1 protein displayed interactions through hydrogen bonding with HIS3761 and ARG3765. Lastly, Figure S23 revealed the interactions between Z1098417322 and WDR5 protein through hydrophobic interactions with PHE133 and hydrogen bonding with TYR191. Correspondingly, MLL1 protein demonstrated interactions through hydrogen bonding with ALA3764 and ARG3765.

#### 3.4.7 Per-Residue MM-GBSA Analysis of WDR5-MLL1 Complex Interaction: Effects of Different Ligands on Residue Energies

The study of protein–protein complex interactions in the presence of various ligands reveals a complex landscape of WDR5-MLL1 interactions and their modulation. In the apo form Figure 8, WDR5-MLL1 complex exhibits an intricate network of interactions, stabilized by multiple water-mediated contacts. This baseline interaction serves as a crucial reference point for understanding the impact of different ligands on the complex. In Figure 9, the introduction of IA9 presents an intriguing case where the ligand, contrary to its intended inhibitory role, enhances the binding between WDR5 protein and MLL1 complex. IA9’s extensive network of hydrogen bonds and hydrophobic interactions with critical WDR5 protein residues, coupled with its occupation of a larger binding surface, suggests a mechanism of action that goes beyond simple inhibition. This unexpected behavior underscores the complexity of protein–ligand interactions and highlights the potential for allosteric effects in modulating protein–protein interfaces.

**Figure 8:**
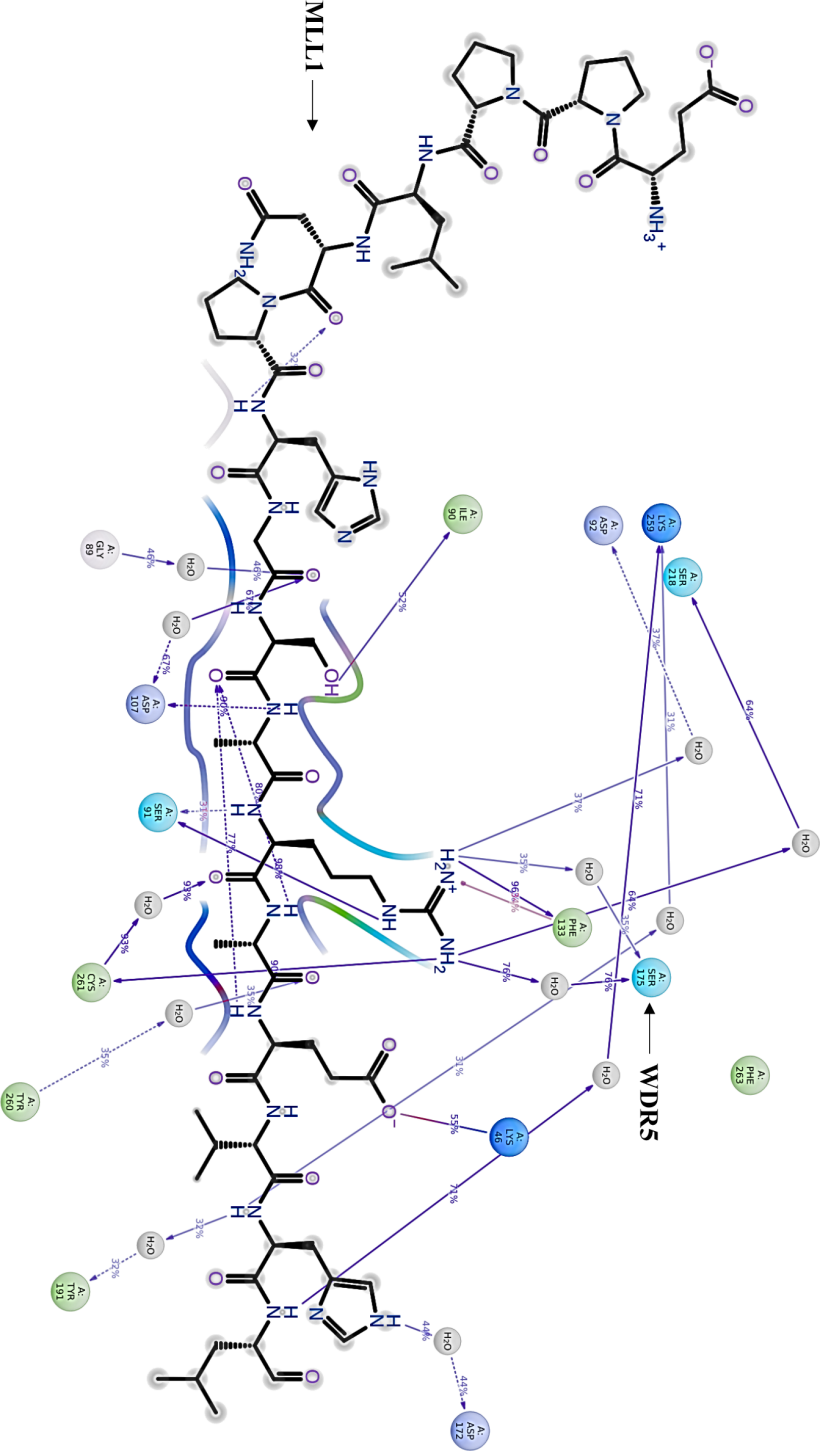
2D illustration shows the residues involved in the interaction between WDR5 and MLL1 (Apo).

**Figure 9:**
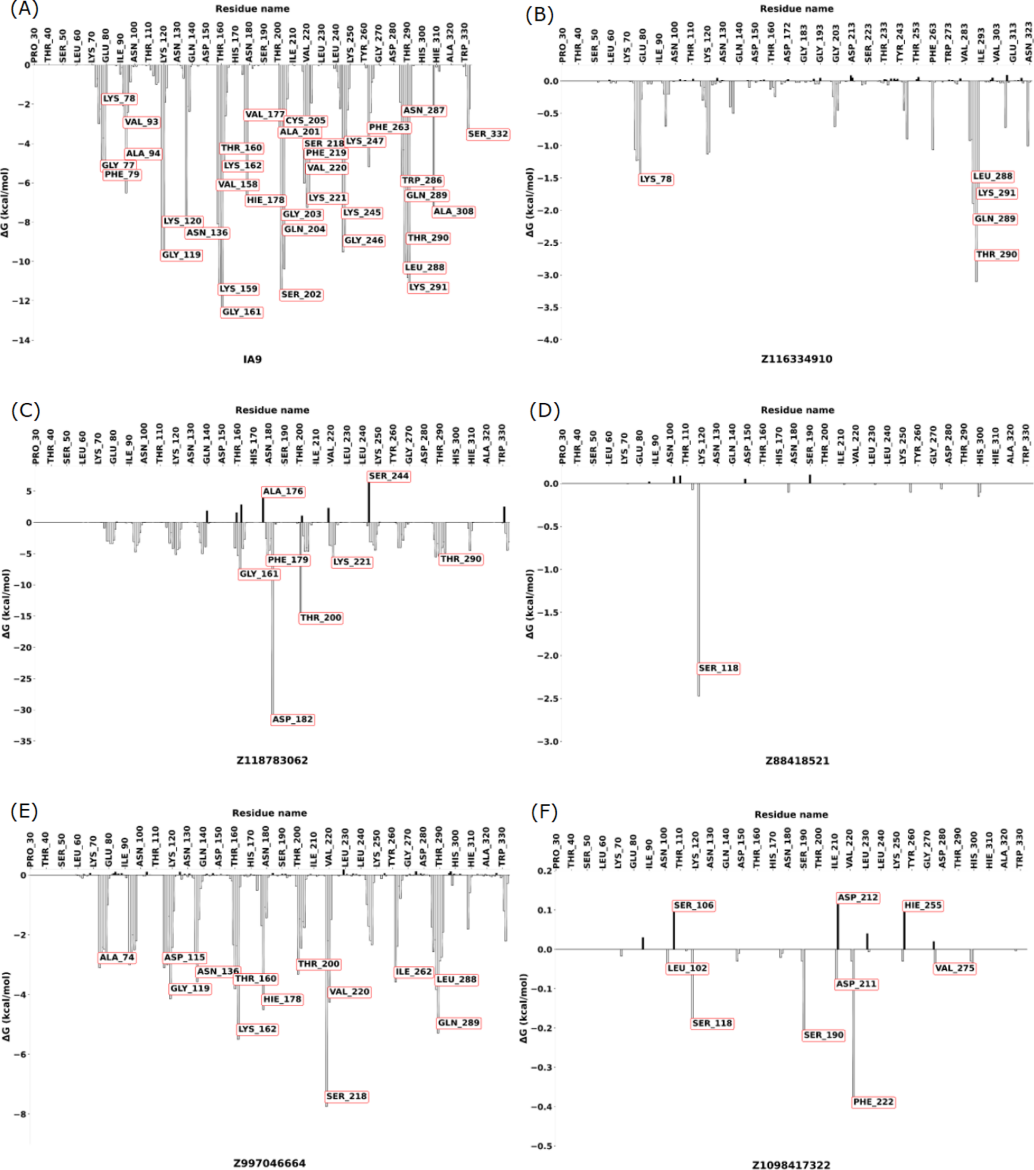
2D illustration shows the residues involved in the interaction between WDR5 and MLL1 in the presence of (A) the reference compound (IA9), (B) Z116334910, (C) Z118783062, (D) Z88418521, (E) Z997046664, and (F) Z1098417322.

In contrast, ligands like Z116334910, Z118783062, and Z997046664 demonstrate more conventional inhibitory effects, albeit through different mechanisms. Z116334910 appears to disrupt native WDR5-MLL1 complex interactions by forming new, strong interactions with specific WDR5 protein residues, particularly in the 288–291 range. This suggests a competitive inhibition mechanism where the ligand effectively displaces MLL1 protein from its binding site. Z118783062, on the other hand, shows an even more dramatic effect, with an exceptionally strong interaction with ASP182. The magnitude of this interaction implies a potential conformational change in WDR5 protein that could significantly alter its ability to bind MLL1. The ligands Z88418521 represents a different approach to inhibition, characterized by highly specific interactions with single residue (SER118). This focused interaction pattern suggests a more targeted inhibition strategy, potentially disrupting key contact points in the WDR5-MLL1 complex interface without causing widespread conformational changes. Such targeted approaches could be particularly valuable in developing inhibitors with high specificity and potentially fewer off-target effects.

Z1098417322 presents yet another inhibition strategy, interacting with multiple residues but with limited binding energies compared to the other ligands. This broader, more distributed interaction profile could lead to a general weakening of the WDR5-MLL1 complex rather than a complete disruption. Such an approach might be useful in situations where a more subtle modulation of the protein–protein interaction is desired. The diverse interaction profiles observed with these ligands highlight the complexity of targeting protein–protein interactions and the potential for developing a range of inhibitors with different mechanisms of action. From co-crystalized ligand IA9 to strong competitive inhibitors like Z118783062, and from targeted disruptors like Z88418521 to broader spectrum modulators like Z1098417322, each ligand offers unique insights into the WDR5-MLL1 complex interaction and potential strategies for its modulation.

These findings have significant implications for drug design and development, particularly in the context of targeting the WDR5-MLL1 complex interaction for therapeutic purposes. The variety of interaction patterns observed suggests that multiple approaches could be viable for developing effective inhibitors. Moreover, the unexpected enhancing effect of IA9 raises intriguing possibilities for developing compounds that could stabilize or enhance specific protein–protein interactions when desired.

## 4 Discussion

The intersection of epigenetic modifications and cancer biology has emerged as a critical area of research over recent decades, with particular focus on methylation processes that regulate gene expression. Our investigation centered on WDR5 protein, a key protein within the MLL1 complex that facilitates active chromatin formation through mono-, di-, and trimethylation of histone H3 at lysine 4. The MLL1 enzyme requires S-adenosyl methionine (SAM) as a cofactor and achieves optimal functionality when assembled with its partner proteins WDR5, RbBP5, ASH2L, and DPY-30 (collectively termed WRAD).^31,44,69–74^ WDR5 protein, characterized by its WD40 repeat structure, serves as a pivotal role in maintaining complex stability and fully activating MLL1 protein's methyltransferase function. Given WDR5 protein’s essential contribution to stabilizing and activating MLL complexes, which drive H3K4 trimethylation and are implicated in various malignancies, it represents a compelling therapeutic target.^71^ Consequently, numerous investigations have pursued small-molecule inhibitors designed to disrupt the WDR5-MLL complex interaction as a promising strategy to suppress MLL1 complex activity.^44,73–76^ Studies have identified *N*-(2-(4-methylpiperazin-1-yl)-5-substituted-phenyl) benzamides as potent antagonists of this interaction. Additionally, short arginine-containing peptides and WIN motif-containing peptides have shown effectiveness in binding WDR5 and reducing H3K4 dimethylation by the MLL core complex in vitro, further supporting the strategy of targeting WDR5 to antagonize the MLL and SET1 family of histone methyltransferases.^44^

In our study, we focused on WDR5 that binds to the WIN motif of MLL1 through this peptidyl arginine-binding cleft. We utilized the crystal structures of WDR5 (PDB: 4IA9)^51^ and the WDR5-MLL1 complex (PDB: 4ESG)^52^ to conduct a critical analysis aimed at identifying novel potential inhibitors.^44^ Our reference molecule was selected based on previous studies, which positioned the benzamide ring in the shallow side cavity of the binding site. The positioning of this ring can be altered depending on the type and position of its substituents, potentially significantly impacting activity.^75^ To better understand the increased potency of compound IA9, Bolshan et al. solved the molecule structure in complex with WDR5 protein (PDB code 4IA9). As anticipated by that study, the compound occupies WDR5 protein’s central cavity and replicates interactions observed with less potent analogs (PDB codes 3SMR and 3UR4), including direct and water-mediated hydrogen bonds with SER91 and CYS261, creation of a cavity occupied by the nitro group, and aromatic stacking with PHE133.

The IA9 structure revealed that the 3-methyl and 4-fluoro substituents contribute hydrophobic interactions with side chains lining the binding pocket, while the section of the ring facing ASP107 remains unsubstituted, suggesting potential avenues for further optimization.^44^ Hydrophobic interactions and water-mediated hydrogen bonds were widely observed in the interactions between both WDR5 and the WDR5-MLL1 complex with IA9. Our investigation compared various ligands with IA9 and revealed that while all compounds occupy the central cavity of WDR5, each demonstrates unique binding characteristics. Ligand Z23687067367 interacts with CYS261, PHE133, and ASP92, forming hydrogen bonds and possible π-stacking interactions. Ligand Z3687055598 demonstrated interactions with SER175, CYS261, and ASP92, suggesting a slightly different binding mode. Ligand IA9 interacts with CYS261, PHE133, and TYR260, indicating yet another binding configuration.

Further analysis of complex interactions revealed that ligand Z88418521 engages with residues ARG3765, ALA3764, PHE149, VAL132, ASP107, and TYR131, demonstrating a complex binding mode with multiple potential hydrogen bonds and hydrophobic interactions. While, Z116334910 displays a simpler interaction profile, primarily engaging with ALA3764 and PHE133. Consequently, these findings provide valuable insights for the development of more potent and selective WDR5-MLL1 complex interaction inhibitors, potentially leading to novel therapeutic approaches for cancers associated with dysregulation of this complex.

The sMD simulation results provide substantial evidence for the different predicted binding affinities and conformational changes observed in the WDR5 protein alone versus the WDR5-MLL1 complex, emphasizing the importance of both protein–ligand and protein–protein–ligand interactions in therapeutic applications. Analysis demonstrated that the WDR5 protein exhibited diverse binding strengths across different ligands. These variations in force profiles indicate that while certain ligands form more robust interactions, others are more readily displaced, suggesting potential avenues for selective targeting in drug design strategies. This differentiation in ligand interactions points toward the possibility of customizing therapies to either enhance or inhibit specific binding profiles as a promising approach for treating MLL-rearranged leukemias.^77^

In contrast, hit compounds targeting the WDR5-MLL1 complex exhibited enhanced stability, as evidenced by higher force peaks during unbinding simulations. Notably, in the complex form, there was greater consistency between conformers A and B unbinding peaks for ligands like Z88418521 and Z997046664, unlike the distinct behavior observed with the isolated WDR5 protein. This consistency likely results from significant conformational changes in the MLL1 protein upon ligand binding, suggesting that MLL1’s structural flexibility is fundamental for accommodating various ligands and enabling potential therapeutic interventions. Consequently, targeting the WDR5-MLL1 complex may represent a more effective strategy than focusing exclusively on the WDR5 protein for disrupting MLL1 protein activity.

Moreover, the data showed that specific residues within WDR5 protein and MLL1 protein exhibited substantial side chain movements, reflecting their adaptive capabilities during ligand binding. Residues such as ALA47, TYR131, PHE149, and LEU234 in WDR5 protein, as well as LEU3770 in MLL1, played pivotal roles in ligand accommodation through hydrophobic interactions and π-stacking mechanisms. The substantial displacements observed in these residues emphasize their functional importance and could inform the design of compounds aimed at stabilizing or destabilizing these interactions. Interestingly, while WDR5 protein maintained a relatively stable backbone structure across ligand interactions, MLL1 protein displayed significant conformational changes, particularly in regions surrounding residues like PRO3756 and LEU3770. This dynamic behavior suggests that MLL1 protein is more flexible and may serve as a primary target for drug development, given its susceptibility to conformational shifts upon ligand binding. The observation that MLL1 protein undergoes pronounced changes while WDR5 protein remains stable could guide future studies to explore the selective modulation of these interactions.

The molecules generated using the BRICS method failed to design better affinity compounds following an extensive 250 ns MD simulations. This outcome underscores the challenges inherent in computational drug design, particularly when targeting complex protein–protein interactions such as the WDR5-MLL1 complex. Despite the initial promise of the BRICS approach in deconstructing molecules into synthetically feasible substructures, the resulting compounds lacked the better predicted binding affinity required to withstand the MD simulation. This setback highlights the importance of thorough validation steps in the drug discovery pipeline.

## 5 Conclusions

In our study, several promising small molecules emerged as inhibitor candidates targeting the WDR5-MLL1 complex interaction, each displaying distinct mechanistic profiles. Z116334910 was a particularly compelling candidate, demonstrating competitive inhibition through robust interactions with WDR5 protein residues 288–291. This compound effectively outcompetes MLL1 protein for its binding site, suggesting significant therapeutic potential. Besides, Z88418521 exhibits more precise targeting mechanisms. These molecules offer the promise of selective inhibition while potentially minimizing broader conformational disruptions to the protein structure. Future research directions can build on our findings through detailed structural analyses to fully characterize the binding mechanisms of these compounds; besides, comprehensive functional studies will be essential to bridge the gap between structural insights and therapeutic effectiveness. Importantly, the findings presented here are entirely computational and serve as a foundational step toward the discovery of WDR5 inhibitors. Experimental validation, including *in vitro* and cellular assays, will be crucial to verify the biological relevance and therapeutic efficacy of these hits. These studies should evaluate how the observed molecular interactions translate into biological outcomes in relevant leukemia models. This work will support the development of more reliable computational methods while maintaining experimental validation standards to ensure effective therapeutic candidates.

## Supplementary Material



## Data Availability

MD simulations (250 ns) generated in this study are publicly available via the Zenodo repository at DOI https://doi.org.//10.5281/zenodo.16695883.

## Notes

This study employed multiple computational approaches to characterize the WDR5 protein system and its complex with MLL1 through MD simulations, binding affinity calculations, and structural analyses. The supporting data include MM/GBSA binding free energy calculations (Δ*G* in kcal/mol) for selected ligands across multiple drug libraries, triplicate 10, 100, and 250 ns MD simulations, molecular docking score distributions for screened compound libraries, RMSD and RMSF analyses of protein backbone stability over simulation trajectories, interaction maps and visualizations illustrating ligand-WDR5 and ligand-WDR5-MLL1 interactions, and comparative analyses of dynamic behavior between WDR5 alone versus WDR5-MLL1 complex states (PDF)
