# Supplementary material for: A Novel Multi-Tiered Hybrid Virtual Screening Pipeline for the Discovery of WDR5-MLL1 Interaction Disruptors in Precision Cancer Therapy
Source: ACS Omega. 2025 Sep 29;10(40):46501–23. doi: 10.1021/acsomega.5c02521 (PMC12529130; doi:10.1021/acsomega.5c02521)
Supplement: Supplementary file 1 [file ao5c02521_si_001.pdf]

SUPPORTING INFORMATION

A Novel Multi-Tiered Hybrid Virtual Screening Pipeline for the Discovery of WDR5-MLL1 Interaction Disruptors in Precision Cancer Therapy

Anwar Abuelrub[a,b,c], Ismail Erol[a,d], Serdar Durdağı[a,b,e*]

[a]Laboratory for Innovative Drugs (Lab4IND), Computational Drug Design Center (HİTMER), Bahçeşehir University, 34734, İstanbul, Türkiye; [b]Computational Biology and Molecular Simulations Laboratory, Department of Biophysics, School of Medicine, Bahçeşehir University, 34734, İstanbul, Türkiye; [c]Graduate School of Natural and Applied Sciences, Artificial Intelligence Program, Bahçeşehir University, 34353, İstanbul, Türkiye; [d]Department of Analytical Chemistry, School of Pharmacy, Bahçeşehir University, 34353, İstanbul, Türkiye; [e]Molecular Therapy Laboratory, Department of Pharmaceutical Chemistry, School of Pharmacy, Bahçeşehir University, 34353, İstanbul, Türkiye

Supplementary Tables

Table S1. MMGBSA ΔG (kcal/mol) means ± SD for selected ligands across libraries targeting WDR5 protein. Triplicate MD simulations with varied timeframes and initial velocities.

		WDR5 Protein															
		10ns (WDR5-Ligand)					100ns (WDR5-Ligand)					250ns (WDR5-Ligand)					
**Library**	**Compound ID**	**1st run**	**2nd run**	**3rd run**	**Average**	**STDV.**	**1st run**	**2nd run**	**3rd run**	**Average**	**STDV.**	**1st run**	**2nd run**	**3rd run**	**Average**	**STDV.**	**Effective score (Average)**
	**IA9**	-52.2	-51.2	-51.2	-51.5	0.6	-49.5	-47.6	-48.8	-48.6	0.8	-44.2	-41.6	-47.6	-44.5	2.5	-1.6
**Enamine**	**Z1218099657**	-83.4	-90.8	-91.7	-88.6	4.6	-84.5	-78.5	-83.2	-82.1	2.6	-62.2	-52.6	-74.9	-63.2	9.1	-2.5
	**Z1551692094**	-82.8	-74.5	-82.3	-79.9	4.7	-79.7	-74.8	-76.8	-77.1	2.0	-77.4	-72.9	-70.7	-73.6	2.8	-2.5
	**Z1754517473**	-78.2	-74.8	-76.1	-76.4	1.7	-75.5	-78.9	-77.2	-77.2	1.4	-70.8	-77.7	-71.4	-73.3	3.1	-2.9
	**Z2690987436**	-84.5	-72.1	-81.6	-79.4	6.5	-73.5	-74.2	-71.4	-73.0	1.2	-75.5	-72.0	-69.0	-72.1	2.6	-3.1
	**Z3687055598**	-86.8	-55.9	-69.8	-70.8	15.5	-76.5	-75.4	-73.0	-74.9	1.5	-65.0	-72.9	-77.3	-71.7	5.1	-2.9
	**Z3687060444**	-81.3	-68.8	-62.5	-70.9	9.6	-69.4	-71.3	-76.5	-72.4	3.0	-56.5	-73.2	-67.8	-65.9	6.9	-2.4
	**Z3687061219**	-84.4	-74.5	-86.6	-81.8	6.5	-75.9	-72.5	-71.9	-73.4	1.8	-71.2	-69.5	-73.1	-71.2	1.5	-2.5
	**Z3687064797**	-81.5	-81.5	-92.3	-85.1	6.2	-78.6	-71.2	-79.5	-76.4	3.7	-72.1	-70.3	-76.7	-73.0	2.6	-2.5
	**Z3687067367**	-81.5	-88.8	-80.0	-83.5	4.7	-84.0	-81.3	-83.0	-82.8	1.1	-83.4	-84.1	-68.3	-78.6	7.3	-2.9
**ChemDiv**	**C875-1275**	-73.8	-77.1	-62.5	-71.1	7.7	-68.4	-67.5	-71.2	-69.1	1.6	-47.8	-62.6	-56.1	-55.5	6.1	-2.4
	**K280-0487**	-85.7	-82.3	-86.3	-84.8	2.2	-71.7	-67.5	-69.3	-69.5	1.7	-69.2	-54.8	-69.5	-64.5	6.9	-2.0
	**N121-0712**	-69.5	-77.2	-73.3	-73.3	3.8	-71.9	-70.1	-68.4	-70.1	1.4	-54.1	-72.1	-80.1	-68.8	10.9	-2.0

Table S2. MMGBSA ΔG (kcal/mol) means ± SD for selected ligands across libraries targeting WDR5-MLL1 complex. Triplicate MD simulations with varied timeframes and initial velocities.

		**WDR5-MLL1 Complex**																				
		**10ns (WDR5-MLL1)**					**100ns (WDR5-MLL1)**					**250ns (WDR5-MLL1)**					**250ns (WDR5-MLL1 Complex-Ligand)**					
**Library**	**Compound ID**	**1st run**	**2nd run**	**3rd run**	**Average**	**STDV.**	**1st run**	**2nd run**	**3rd run**	**Average**	**STDV.**	**1st run**	**2nd run**	**3rd run**	**Average**	**STDV.**	**1st run**	**2nd run**	**3rd run**	**Average**	**STDV.**	**Effective score (Average)**
	**Apo**	-100.3	-122.7	-112.7	-111.9	11.2	-119.4	-107.9	-129.5	-118.9	10.8	-117.1	-135.6	-106.3	-119.7	12.1	-117.1	-135.6	-106.1	-119.6	12.2	--
	**IA9**	-94.0	-82.6	-74.5	-83.7	9.8	-83.1	-82.9	-83.9	-83.3	0.5	-86.5	-79.4	-75.9	-80.6	4.4	-70.2	-79.4	-75.9	-75.2	3.8	-2.7
**Specs**	**AK-968/41927098**	-58.6	-65.3	-80.9	-68.3	11.4	-60.3	-59.0	-62.7	-60.6	1.9	-103.8	-68.6	-74.0	-82.1	15.5	-69.0	-59.1	-74.0	-67.4	6.2	-3.5
	**A0-548/43379527**	-64.1	-57.3	-88.9	-70.1	16.7	-67.7	-67.6	-65.3	-66.9	1.4	-88.6	-97.0	-70.3	-85.3	11.1	-58.5	-97.0	-70.4	-75.3	16.1	-3.6
**Enamine**	**Z997046664**	-64.4	-54.2	-60.2	-59.6	5.1	-67.0	-73.8	-55.9	-65.6	9.1	-68.2	-70.4	-72.4	-70.3	1.7	-74.8	-70.4	-72.4	-72.5	1.8	-3.0
	**Z88418521**	-75.7	-83.2	-60.6	-73.1	11.5	-60.6	-79.0	-67.7	-69.1	9.3	-61.5	-92.1	-83.1	-78.9	12.8	-74.0	-92.1	-101.7	-89.3	11.5	-3.9
	**Z19648368**	-63.7	-73.7	-61.4	-66.3	6.5	-58.0	-73.1	-72.6	-67.9	8.6	-81.4	-84.9	-90.7	-85.7	3.8	-66.3	-84.9	-90.7	-80.6	10.4	-3.4
	**Z1677759102**	-80.1	-81.0	-61.6	-74.2	11.0	-85.7	-59.1	-54.3	-66.4	16.9	-85.1	-81.9	-70.3	-79.1	6.4	-77.0	-68.6	-57.7	-67.8	7.9	-3.2
	**Z1430614506**	-74.7	-66.8	-63.5	-68.3	5.8	-69.5	-57.7	-75.0	-67.4	8.8	-80.8	-73.3	-101.9	-85.4	12.1	-73.2	-73.3	-101.9	-82.8	13.5	-3.8
	**Z118783062**	-75.5	-91.2	-68.9	-78.5	11.5	-61.2	-65.3	-61.0	-62.5	2.4	-63.3	-86.3	-69.5	-73.0	9.7	-73.3	-86.3	-64.3	-74.7	9.0	-3.1
	**Z116334910**	-67.1	-50.0	-100.1	-72.4	25.5	-63.2	-48.2	-80.8	-64.1	16.3	-64.3	-83.1	-77.4	-74.9	7.9	-71.4	-83.1	-77.3	-77.3	4.8	-3.4
	**Z1098417322**	-68.8	-69.0	-68.5	-68.8	0.3	-65.1	-60.9	-54.6	-60.2	5.3	-70.4	-72.8	-78.7	-73.9	3.49	-60.4	-72.8	-78.6	-70.6	7.6	3.0

Table S3. MMGBSA ΔG (kcal/mol) means for top scored generated ligands with WDR5-MLL1 complex. Triplicate MD simulations 250ns.

Name	Smiles	2D Structure	WDR5-MLL1				WDR5-MLL1 Complex-Ligand				
			Run_1	Run_2	Run_3	Average	Run_1	Run_2	Run_3	Average	Effective score (Average)
**A1**	c1cccc(c12)cccc2C(=O)NCOCc3cc(Cl)c(F)cc3	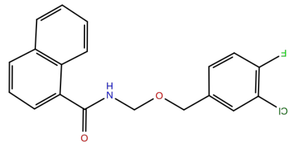	-72.6	-86.2	79.5	-79.4	-74.6	-79.7	-75.5	-76.6	-3.2
**A2**	o1cccc1-c2c(occ2)C(=O)NCOCc3cc(Cl)c(F)cc3	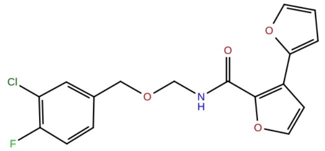	-81.5	-72.3	-88.7	-80.8	-74.8	-86.0	-68.3	-76.4	-3.2
**A3**	c1ccccc1-c2c(occ2)C(=O)NCOc(c3N)cc(Cl)c(c3)F	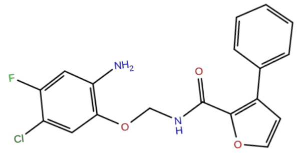	-73.6	-80.4	-84.2	-79.4	-78.5	-65.9	-87.2	-77.2	-3.1
**A4**	c1cc(F)c(Cl)cc1C2C(C2)NC(=O)C[N+]CNC(=O)c3c(F)cncc3	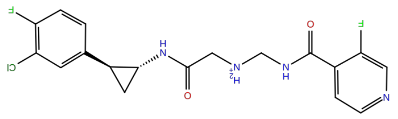	-59.7	-73.4	-73.1	-68.7	-69.9	-70.1	-65.0	-68.3	-2.5
**A5**	c1cncc(F)c1C(=O)NC(C(N)=O)Cc(c2)cc(F)c(Cl)c2C3CC3	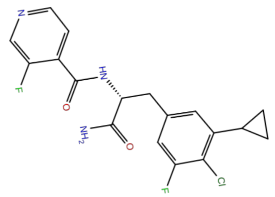	-59.2	-97.9	-74.9	-77.3	-58.7	-77.1	-77.9	-71.2	-2.7
**A6**	o1cccc1-c2c(occ2)-c3c(N)c(oc3)-c4cc(N)c(F)c(c4)Cl	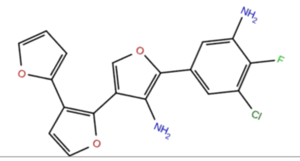	-81.2	-89.1	-89.4	-86.6	-81.2	-72.3	-74.6	-76.1	-3.0
**A7**	o1cccc1C2C(C2)c3ccc(cc3)-c4c(F)c(Cl)ccc4N	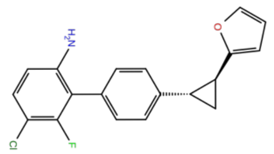	-66.5	-67.4	-90.0	-74.6	-66.5	-66.5	-72.0	-68.3	-3.0

Supplementary Figures

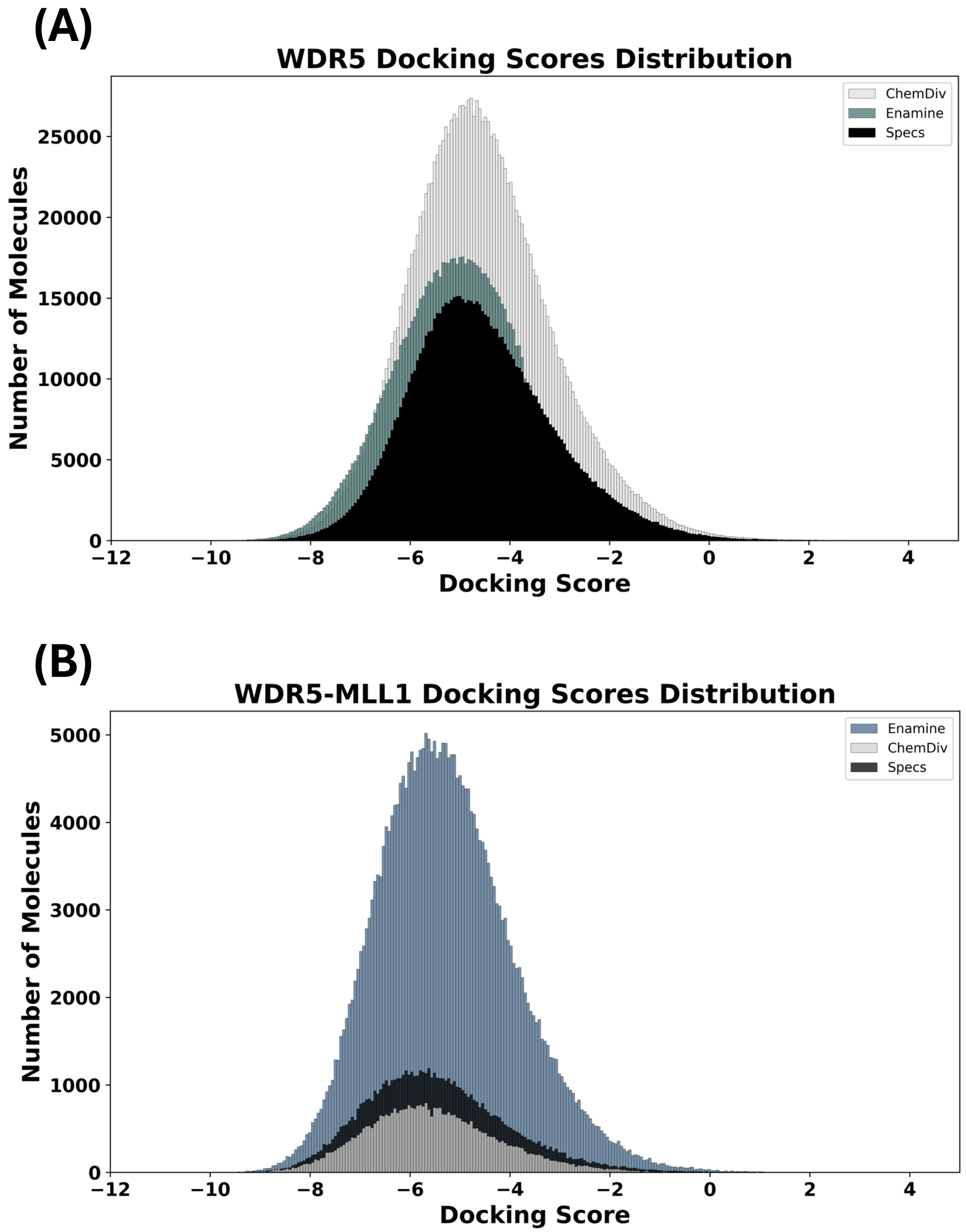

Figure S1. Distribution of molecular docking scores for compounds from the Enamine, ChemDiv, and Specs libraries targeting (A) WDR5 protein and (B) WDR5-MLL1 complex.

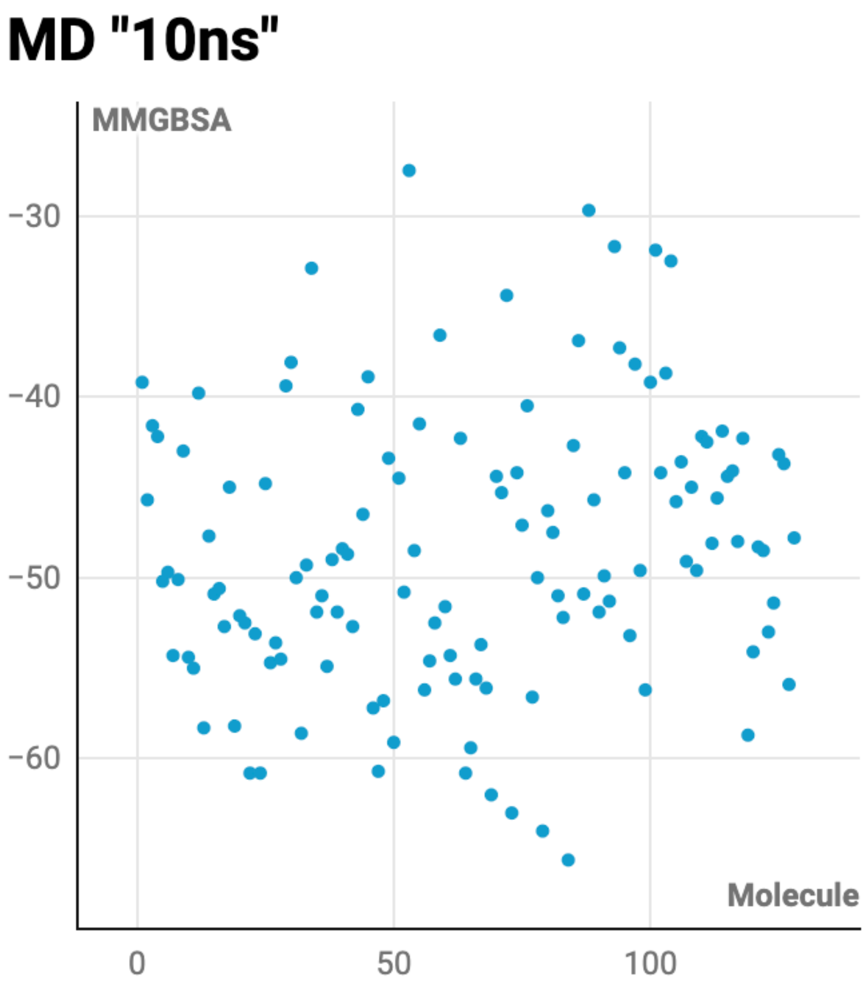

Figure S2. Average MMGBSA values (kcal/mol) of generated molecules targeting the WDR5 protein, derived from 10ns MD simulations (n=3).

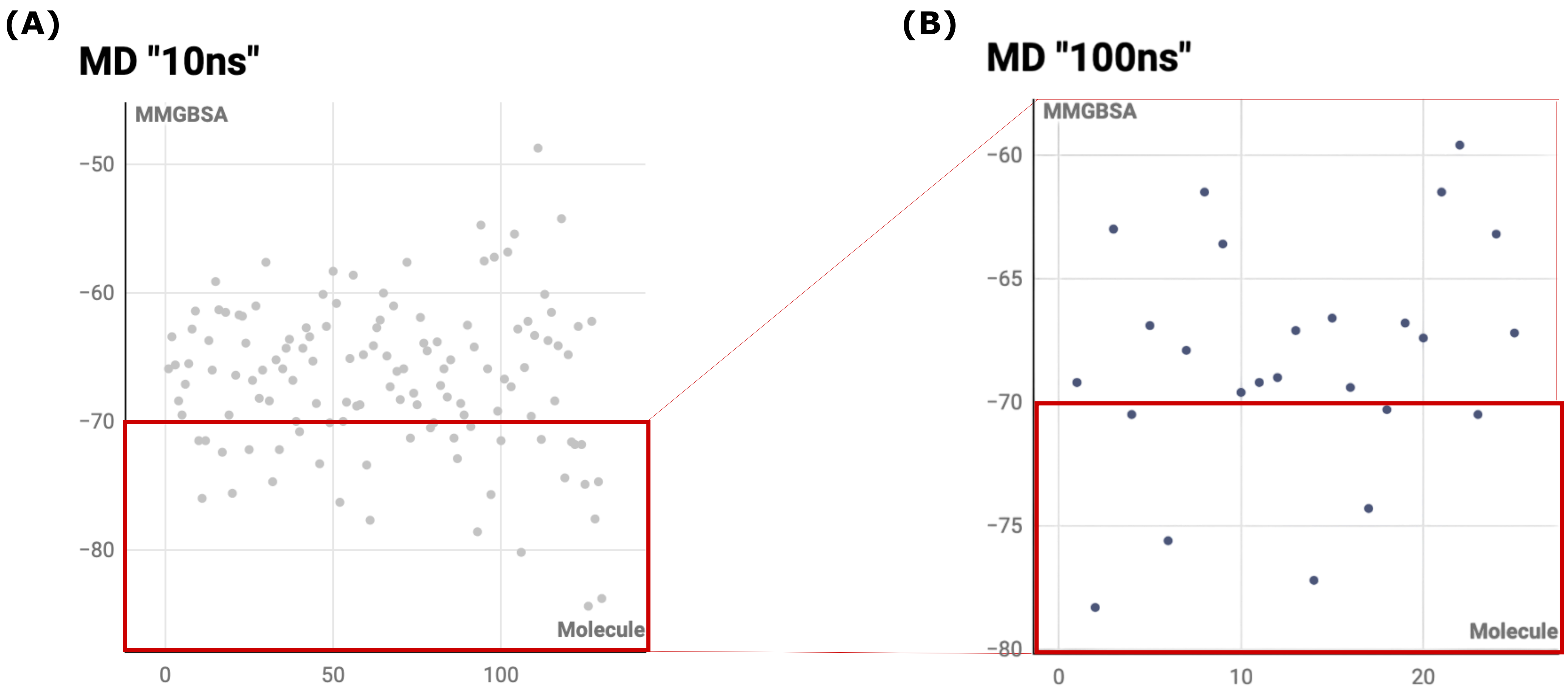

Figure S3. (A) Average MMGBSA values (kcal/mol) of generated molecules targeting the WDR5-MLL1 complex interaction, derived from 10ns MD simulations (n=3) with -70 threshold. **(B)** Average MMGBSA values of selected molecules targeting the WDR5-MLL1, derived from 100ns MD simulations (n=3) with -70 threshold.

(A)

(B)

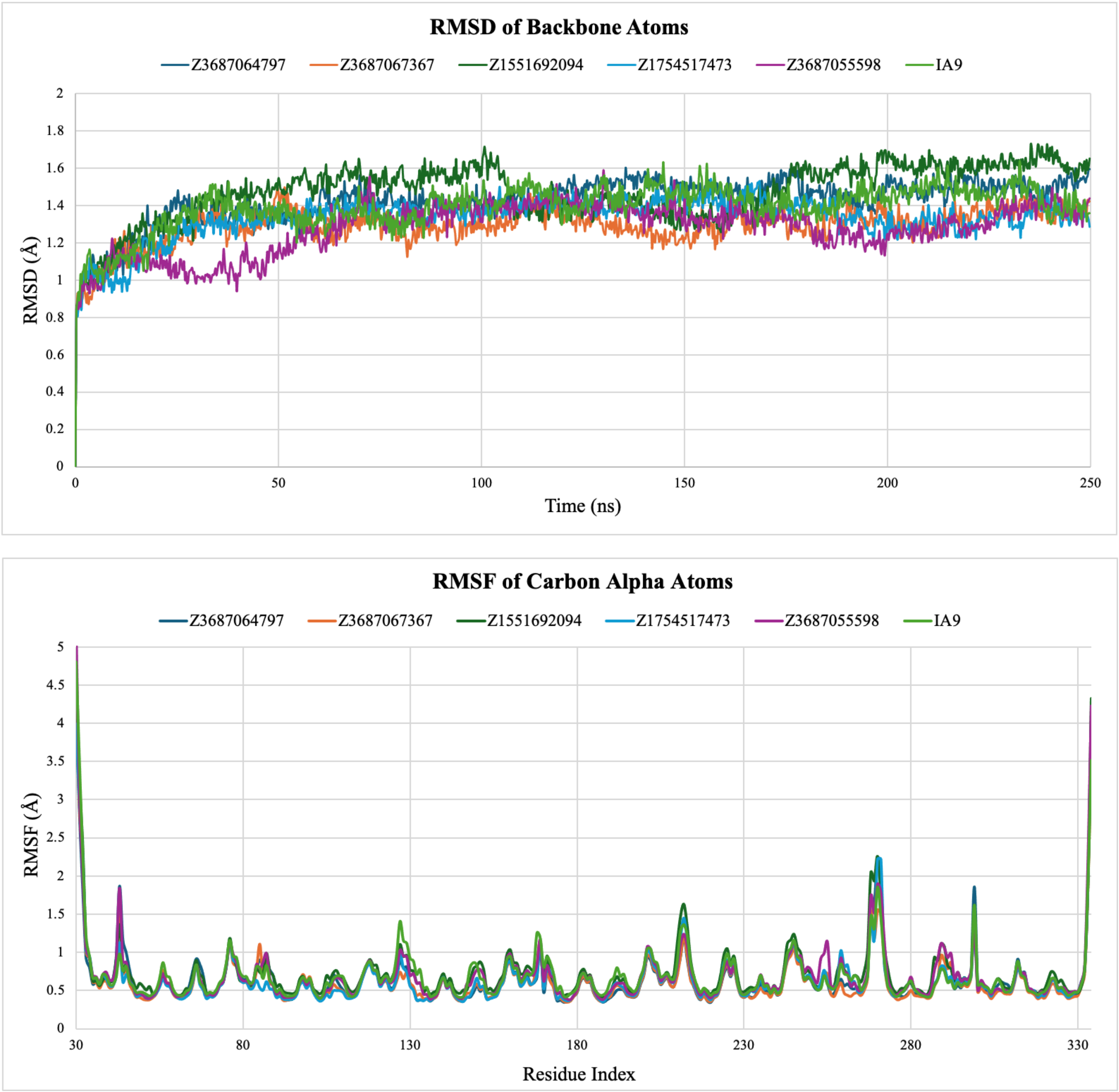

Figure S4. (**A**) Root Mean Square Deviation (RMSD) and (**B**) Root Mean Square Fluctuation (RMSF), concerning the initial confirmation of the WDR5 protein backbone versus the simulation time (250 ns) n=3 in the production simulations step with Z3687064797, Z3687067367, Z1551692094, Z1754517473, Z3687055598, and IA9.

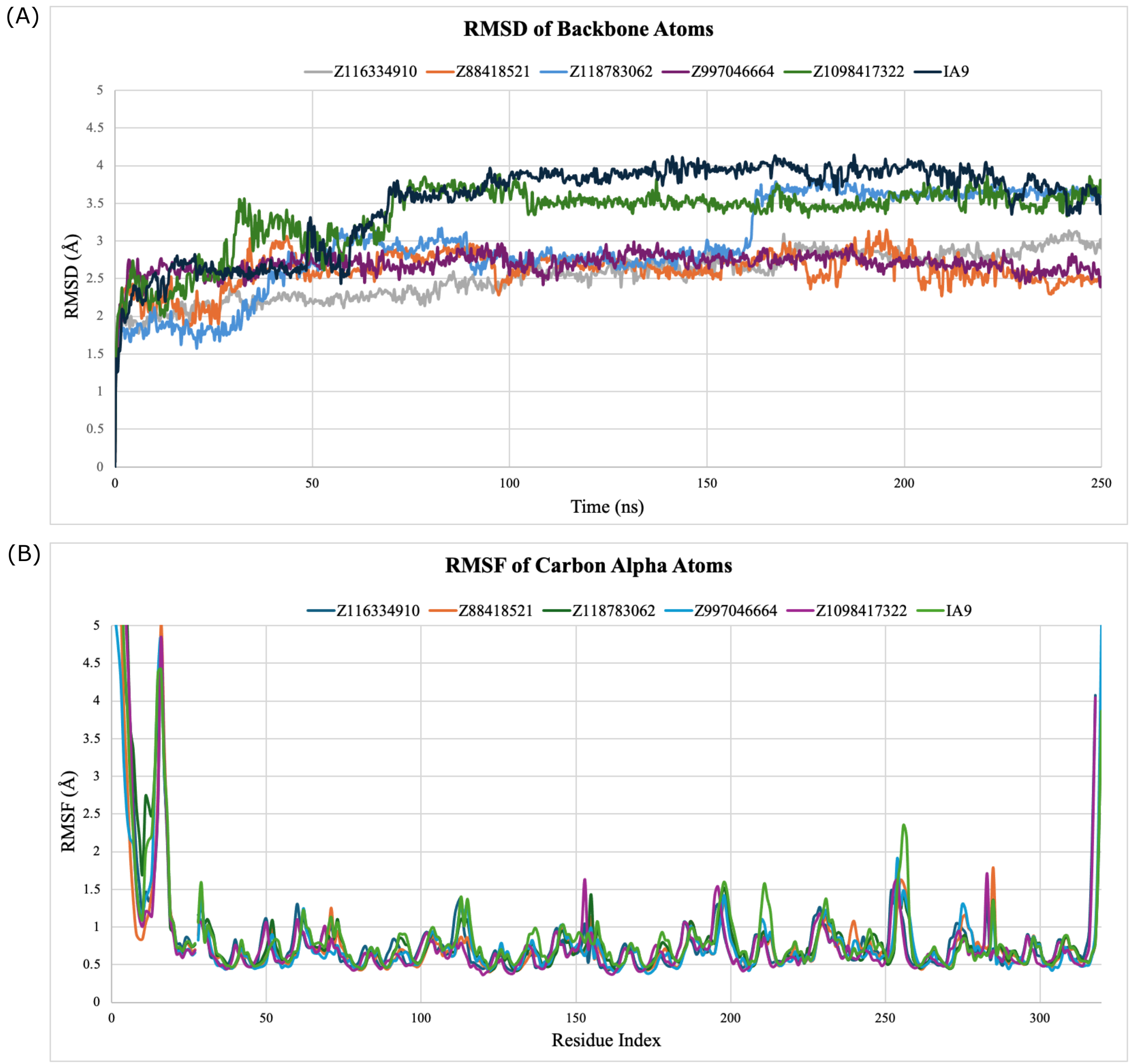

Figure S5. (**A**) Root Mean Square Deviation (RMSD) and (**B**) Root Mean Square Fluctuation (RMSF), concerning the initial confirmation of the WDR5-MLL1 complex backbone versus the simulation time (250 ns) n=3 in the production simulations step with Z116334910, Z88418521, Z118783062, Z997046664, Z1098417322, and IA9.

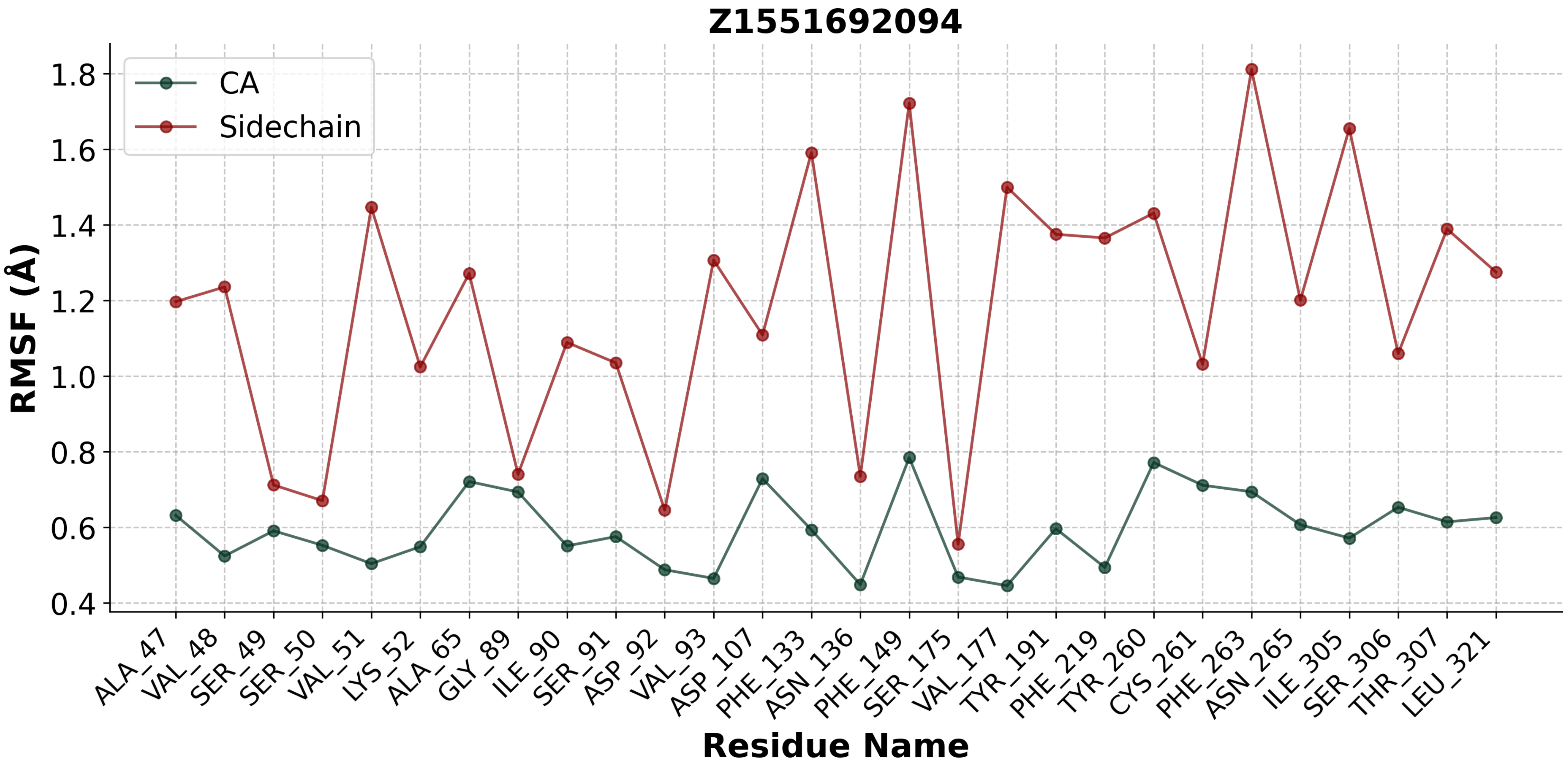

Figure S6. WDR5 protein's conformational adjustments in its CA and sidechain positions while interacting with Z1551692094 during 250 ns of simulations (n=3).

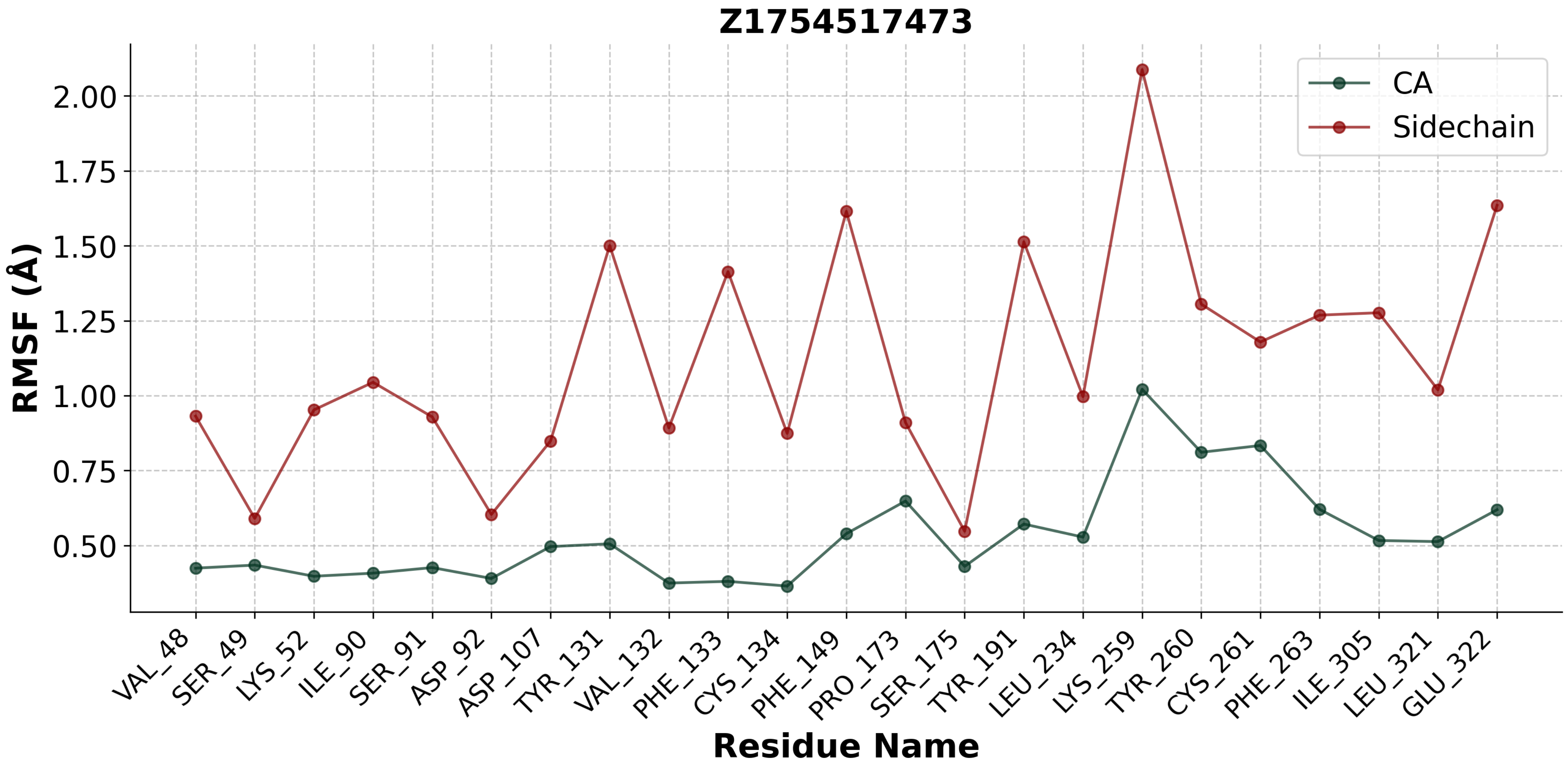

Figure S7. WDR5 protein's conformational adjustments in its CA and sidechain positions while interacting with Z1754517473 during 250 ns of simulations (n=3).

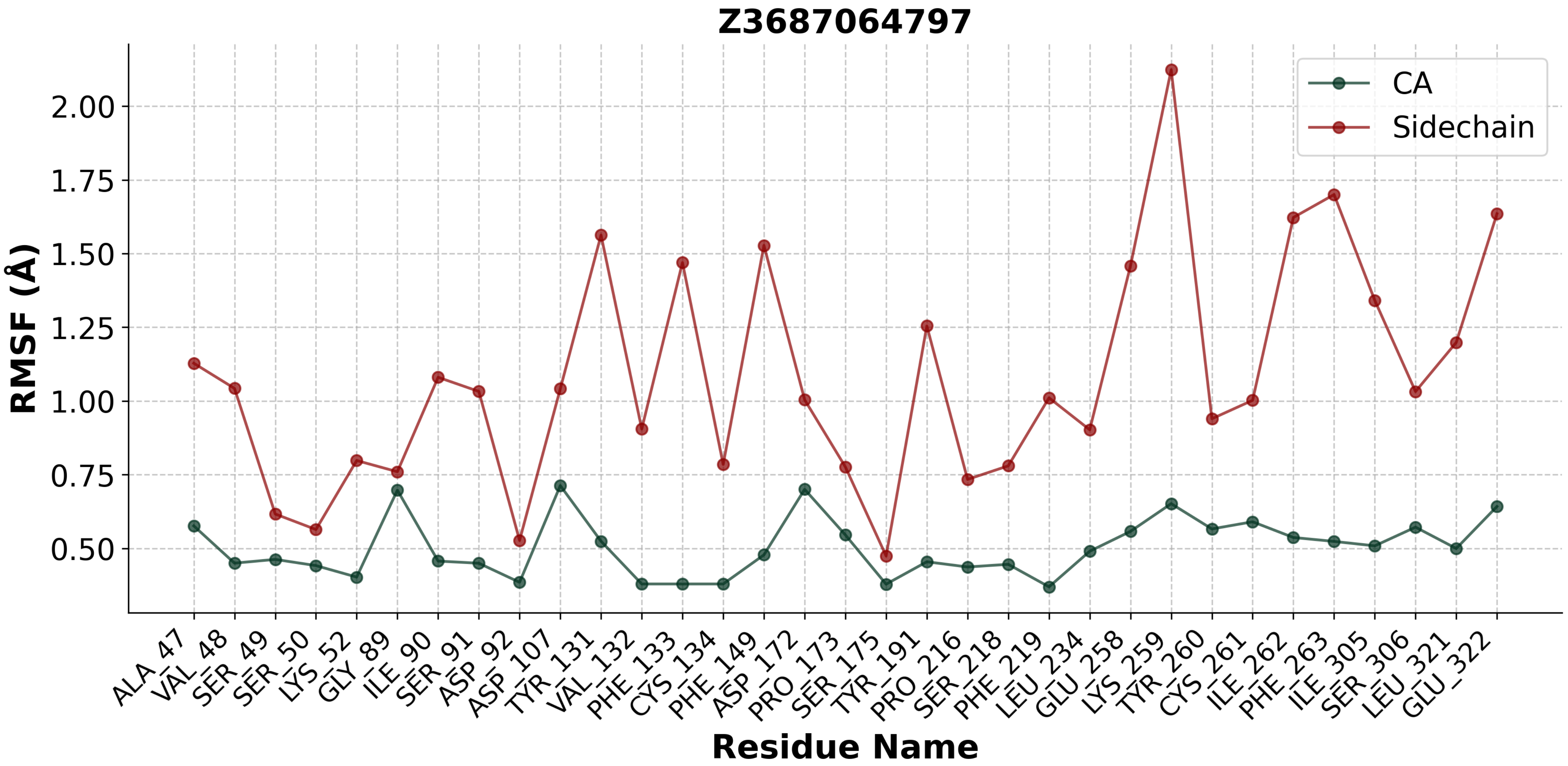

Figure S8. WDR5 protein's conformational adjustments in its CA and sidechain positions while interacting with Z3687064797 during 250 ns of simulations (n=3).

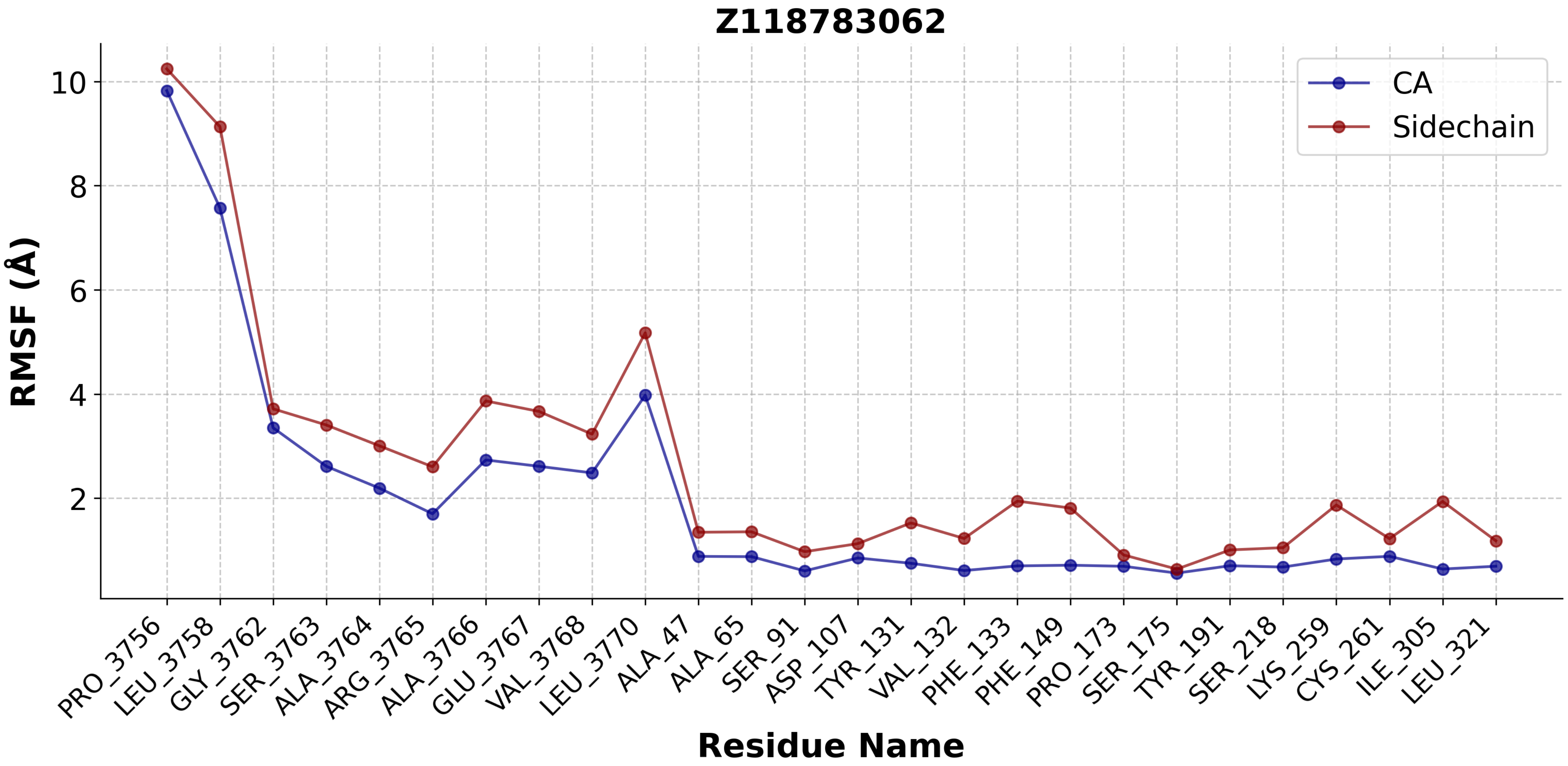

Figure S9. WDR5-MLL1 complex's conformational adjustments in its CA and sidechain positions while interacting with Z118783062 during 250 ns of simulations (n=3).

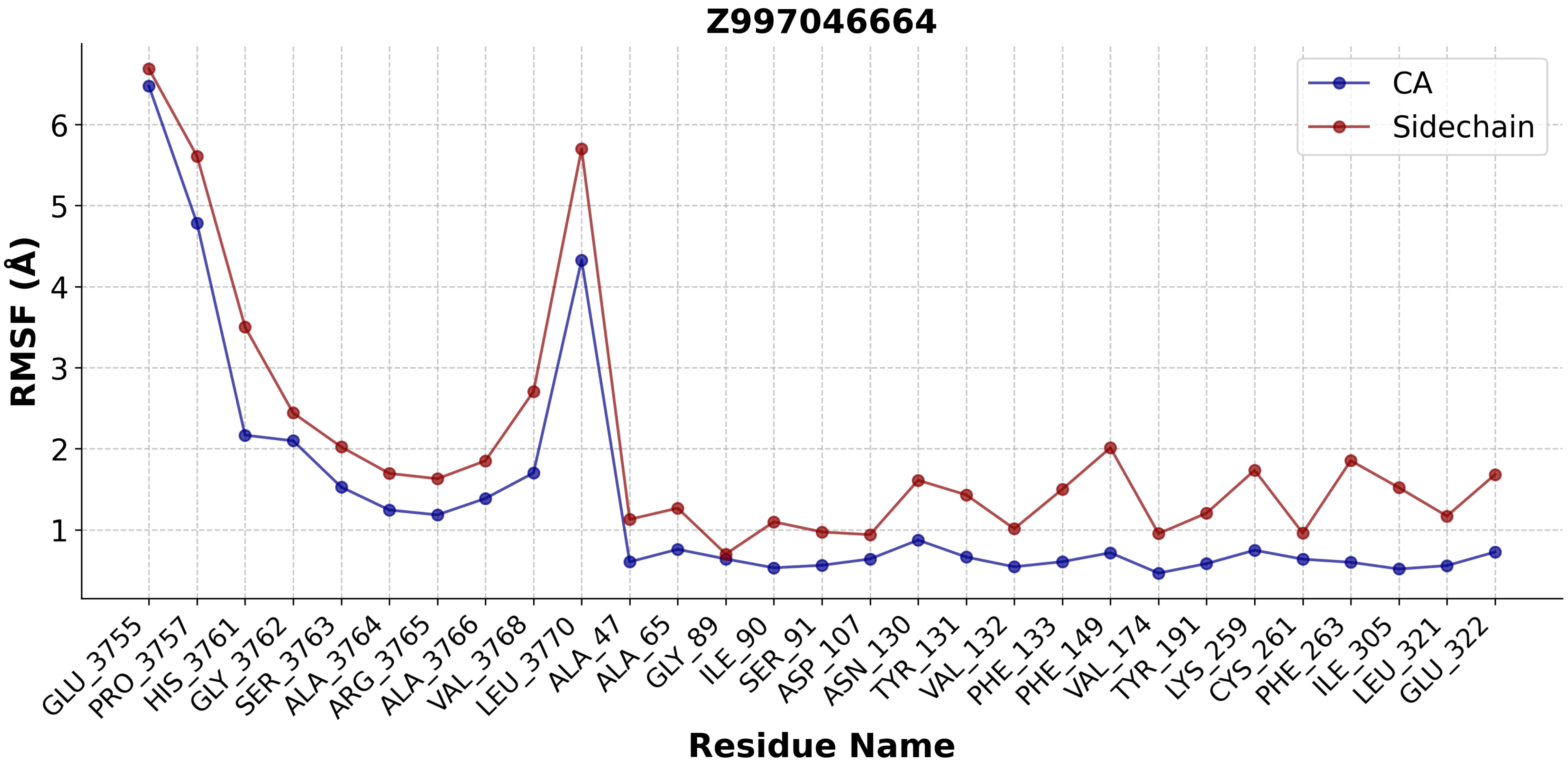

Figure S10. WDR5-MLL1 complex's conformational adjustments in its CA and sidechain positions while interacting with Z997046664 during 250 ns of simulations (n=3).

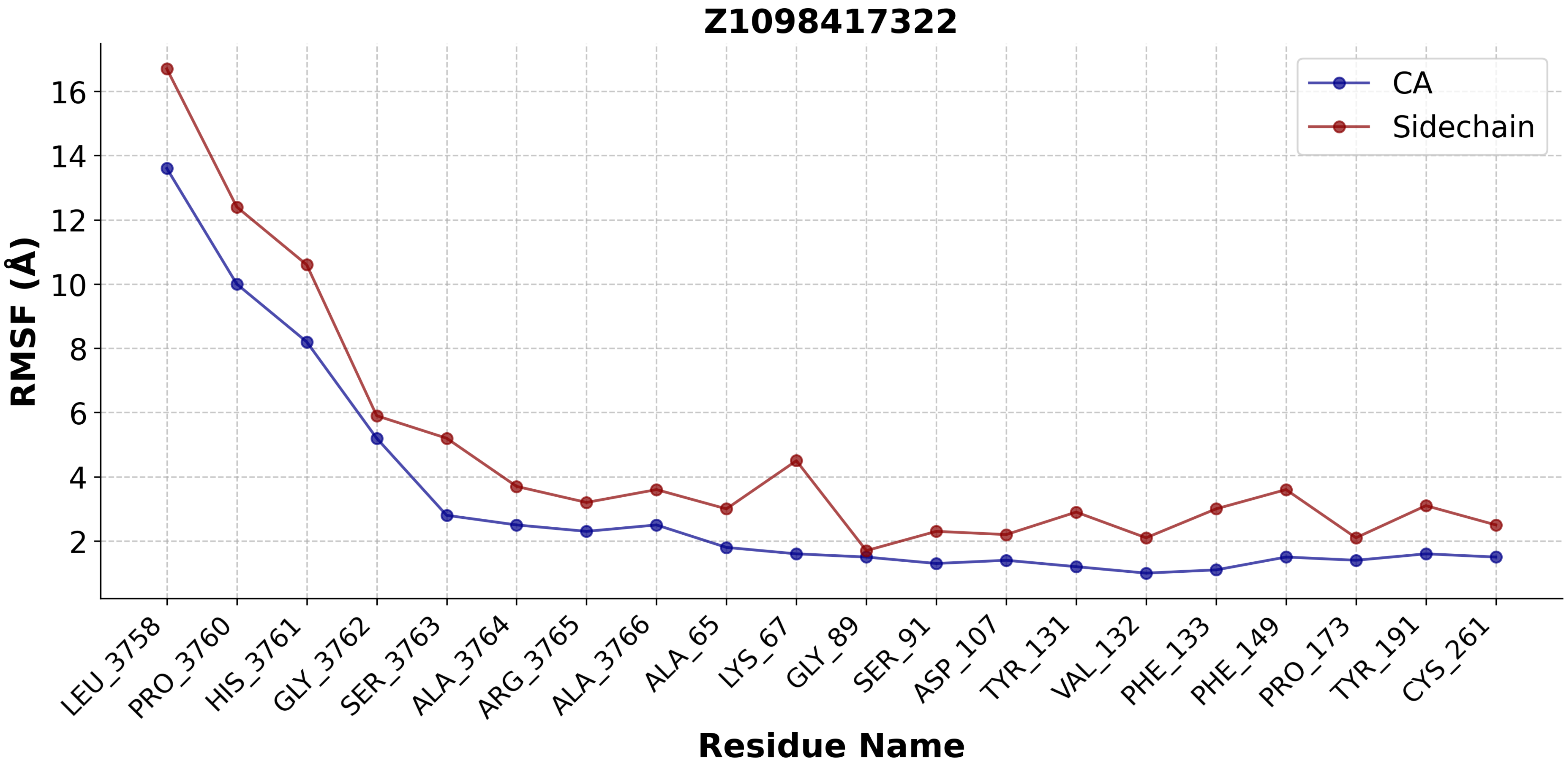

Figure S11. WDR5-MLL1 complex's conformational adjustments in its CA and sidechain positions while interacting with Z1098417322 during 250 ns of simulations (n=3).

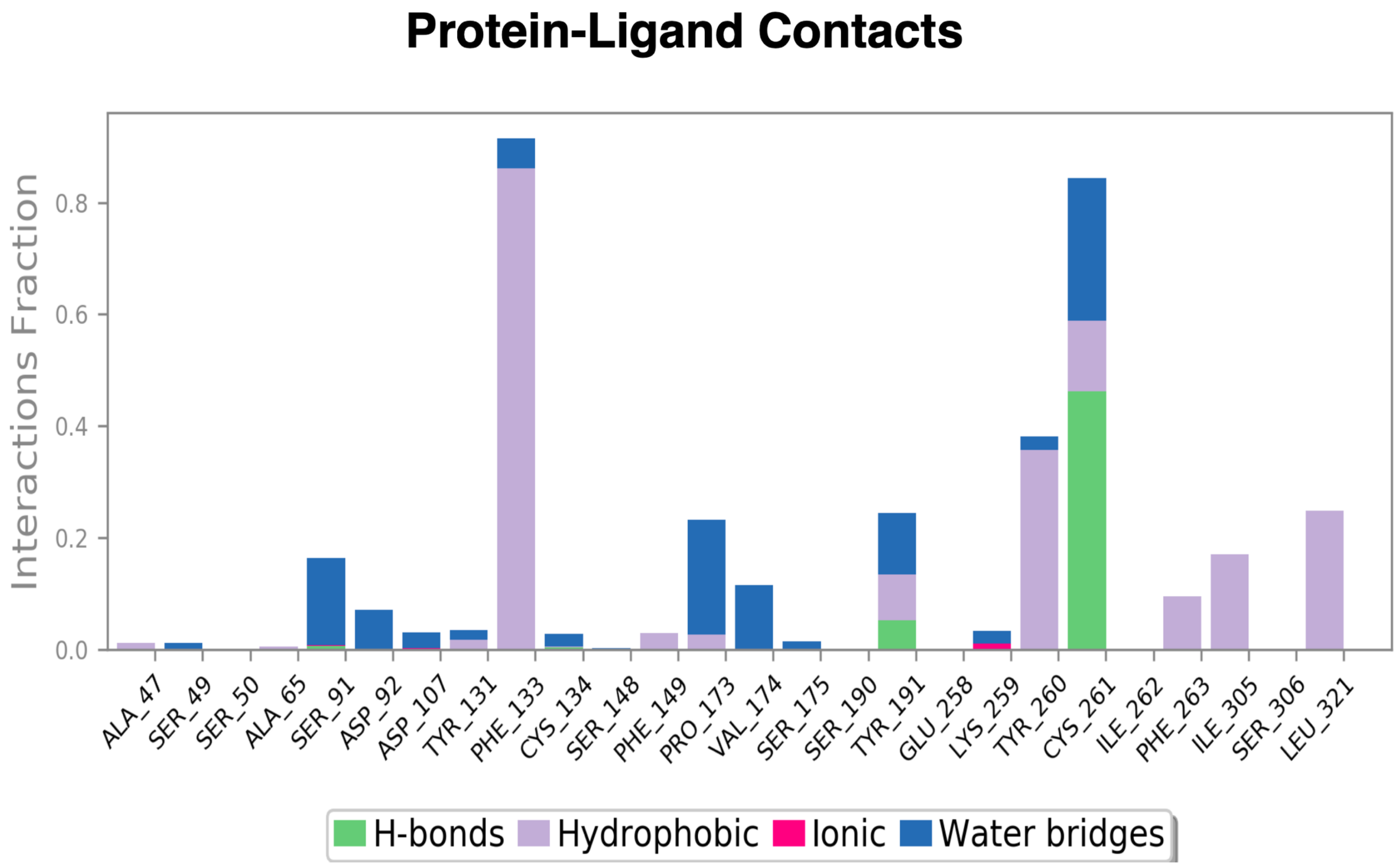

Figure S12. The bar chart illustrates the interactions formed between the drug IA9 and the WDR5 protein, highlighting hydrogen bonds, hydrophobic interactions, ionic bonds, and water bridges during 250 ns molecular simulations.

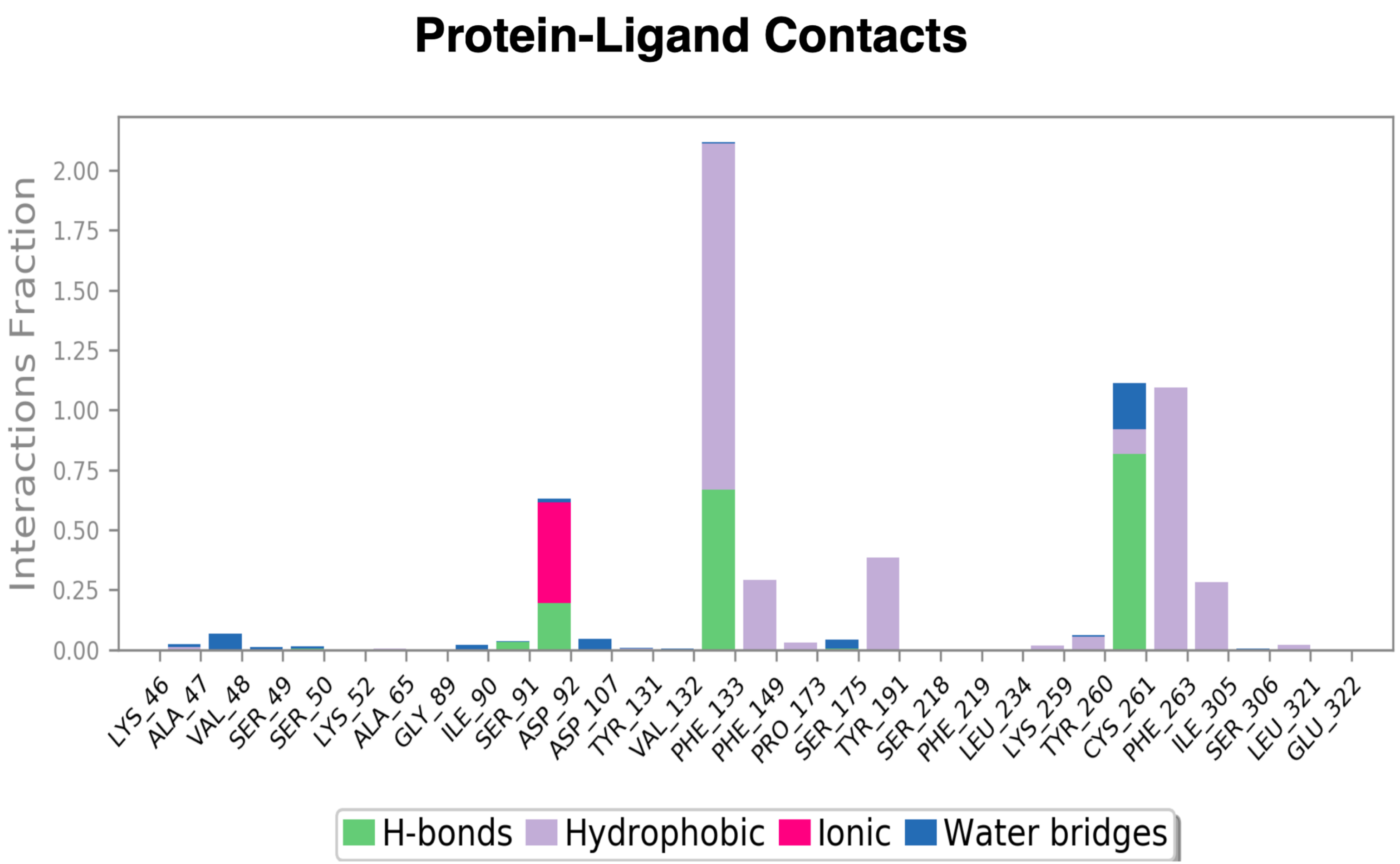

Figure S13. The bar chart illustrates the interactions formed between the drug Z3687067367 and the WDR5 protein, highlighting hydrogen bonds, hydrophobic interactions, ionic bonds, and water bridges during 250 ns molecular simulations.

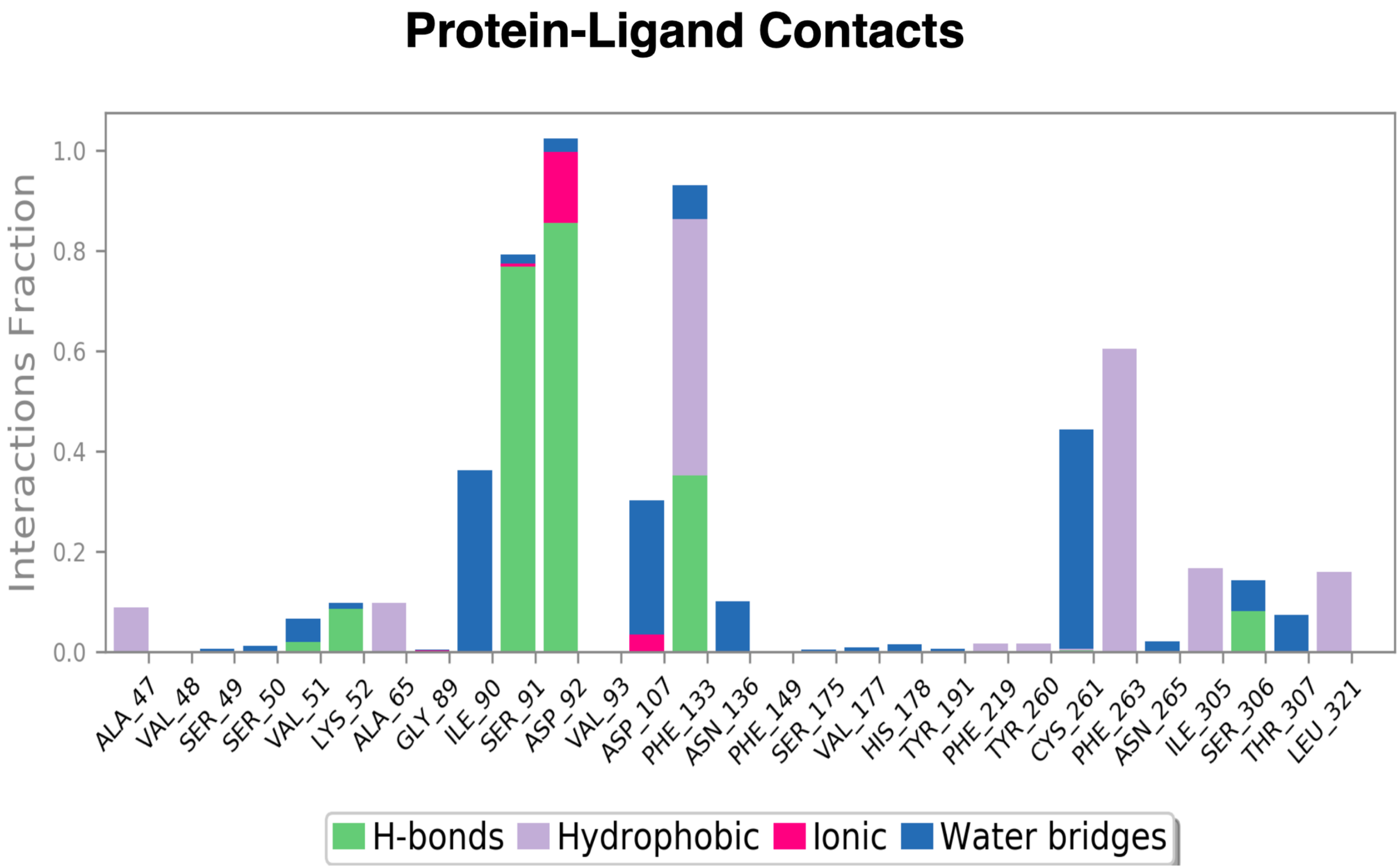

Figure S14. The bar chart illustrates the interactions formed between the drug Z1551692094 and the WDR5 protein, highlighting hydrogen bonds, hydrophobic interactions, ionic bonds, and water bridges during 250 ns molecular simulations.

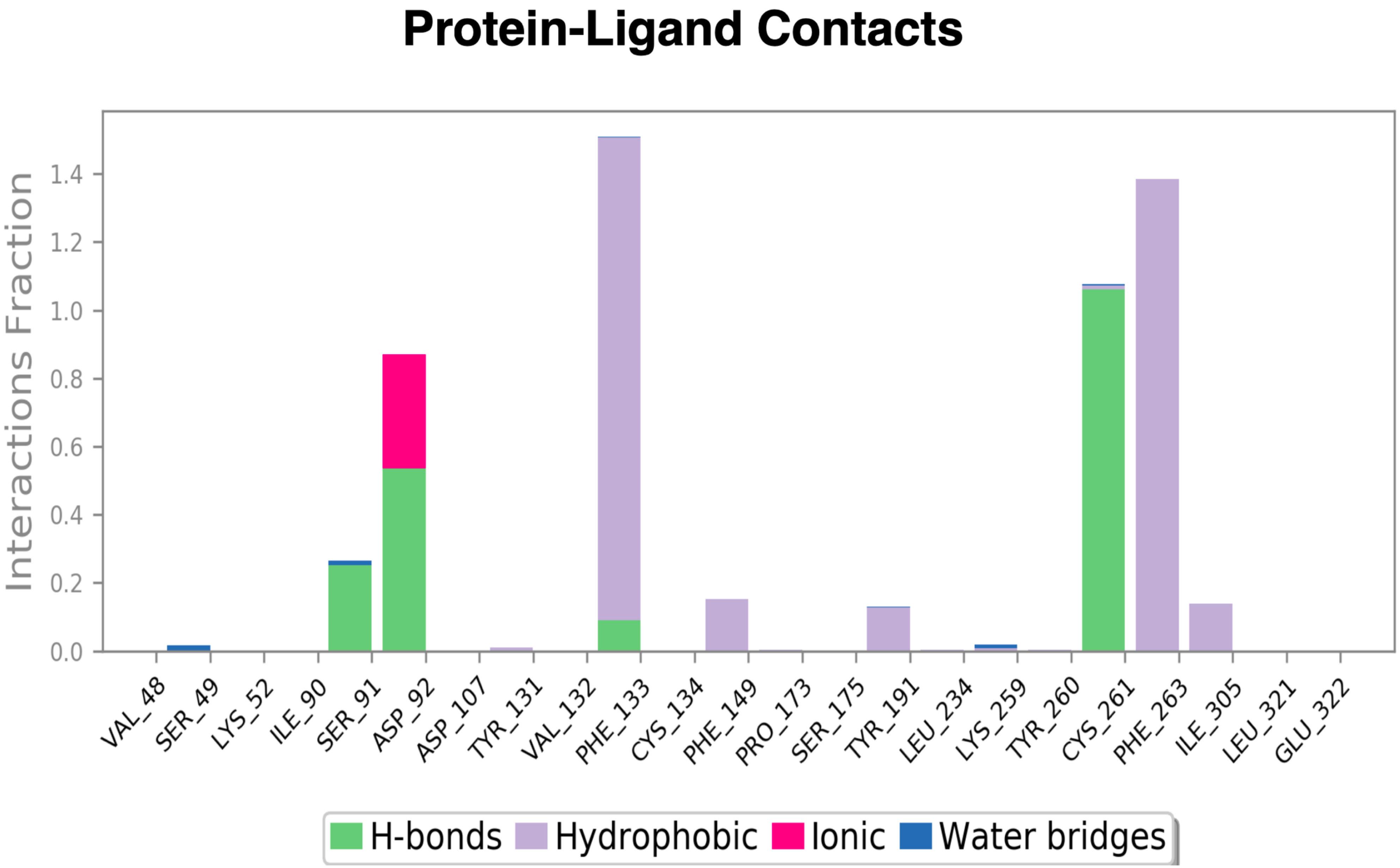

Figure S15. The bar chart illustrates the interactions formed between the drug Z1754517473 and the WDR5 protein, highlighting hydrogen bonds, hydrophobic interactions, ionic bonds, and water bridges during 250 ns molecular simulations.

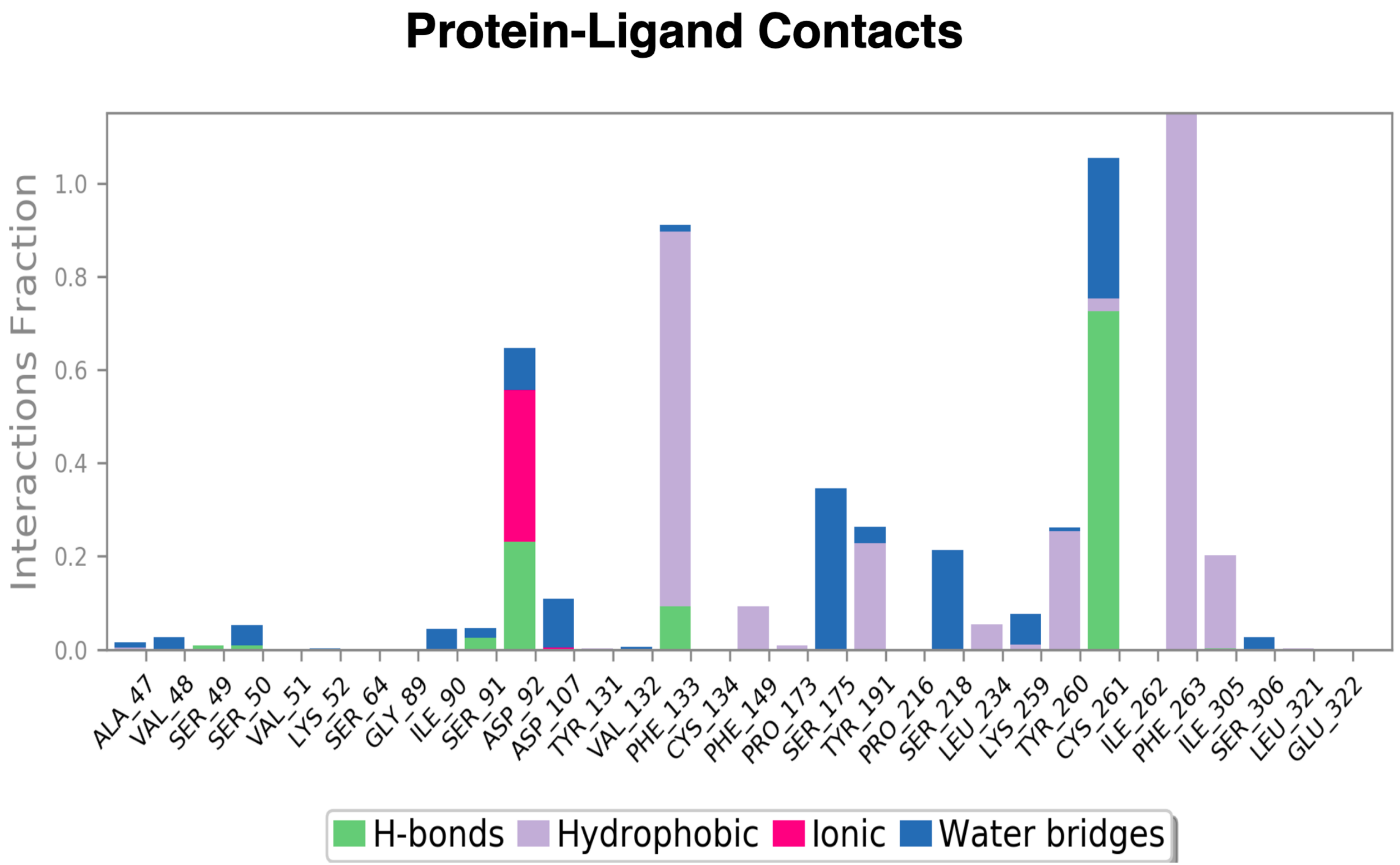

Figure S16. The bar chart illustrates the interactions formed between the drug Z3687055598 and the WDR5 protein, highlighting hydrogen bonds, hydrophobic interactions, ionic bonds, and water bridges during 250 ns molecular simulations.

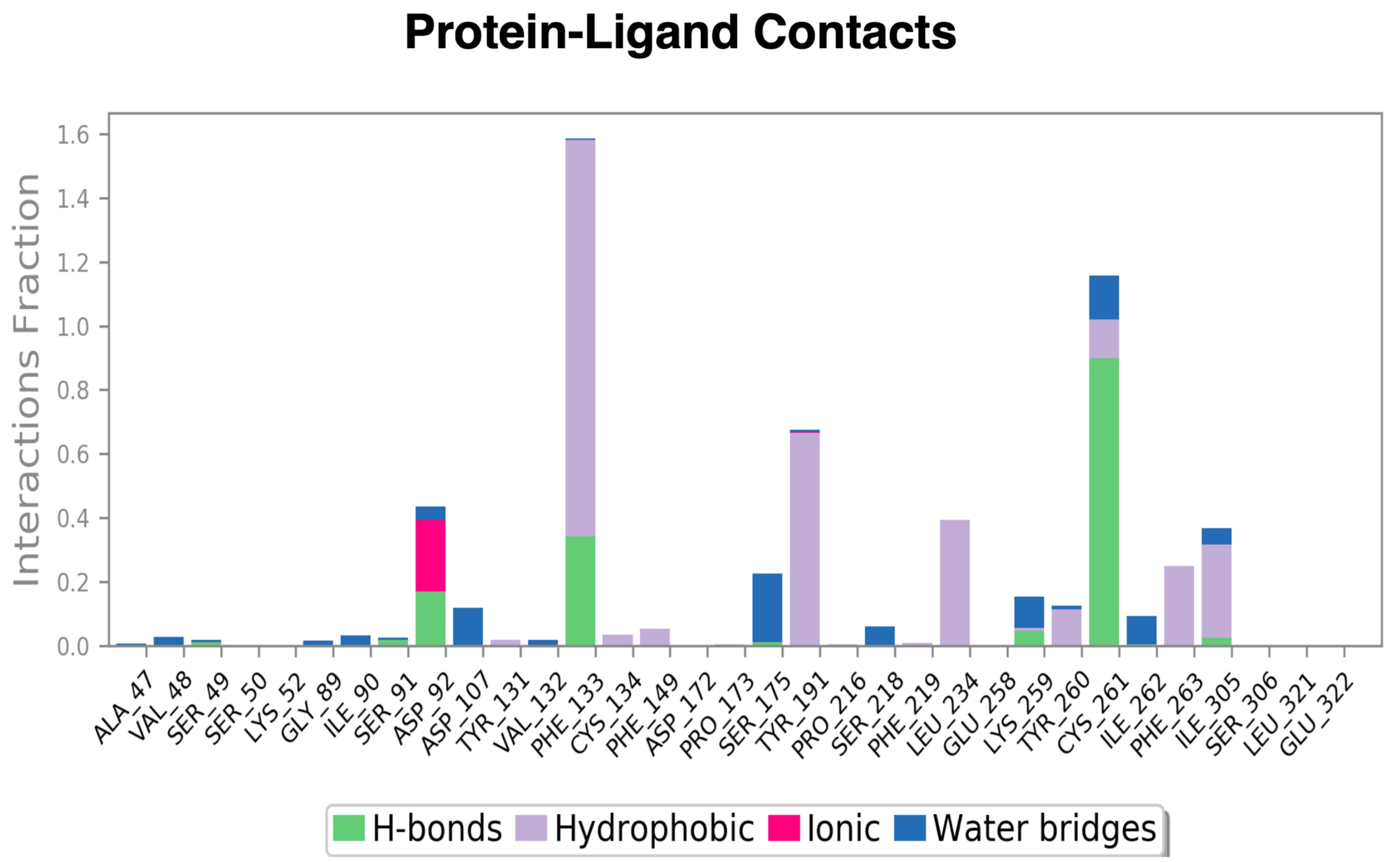

Figure S17. The bar chart illustrates the interactions formed between the drug Z3687064797 and the WDR5 protein, highlighting hydrogen bonds, hydrophobic interactions, ionic bonds, and water bridges during 250 ns molecular simulations.

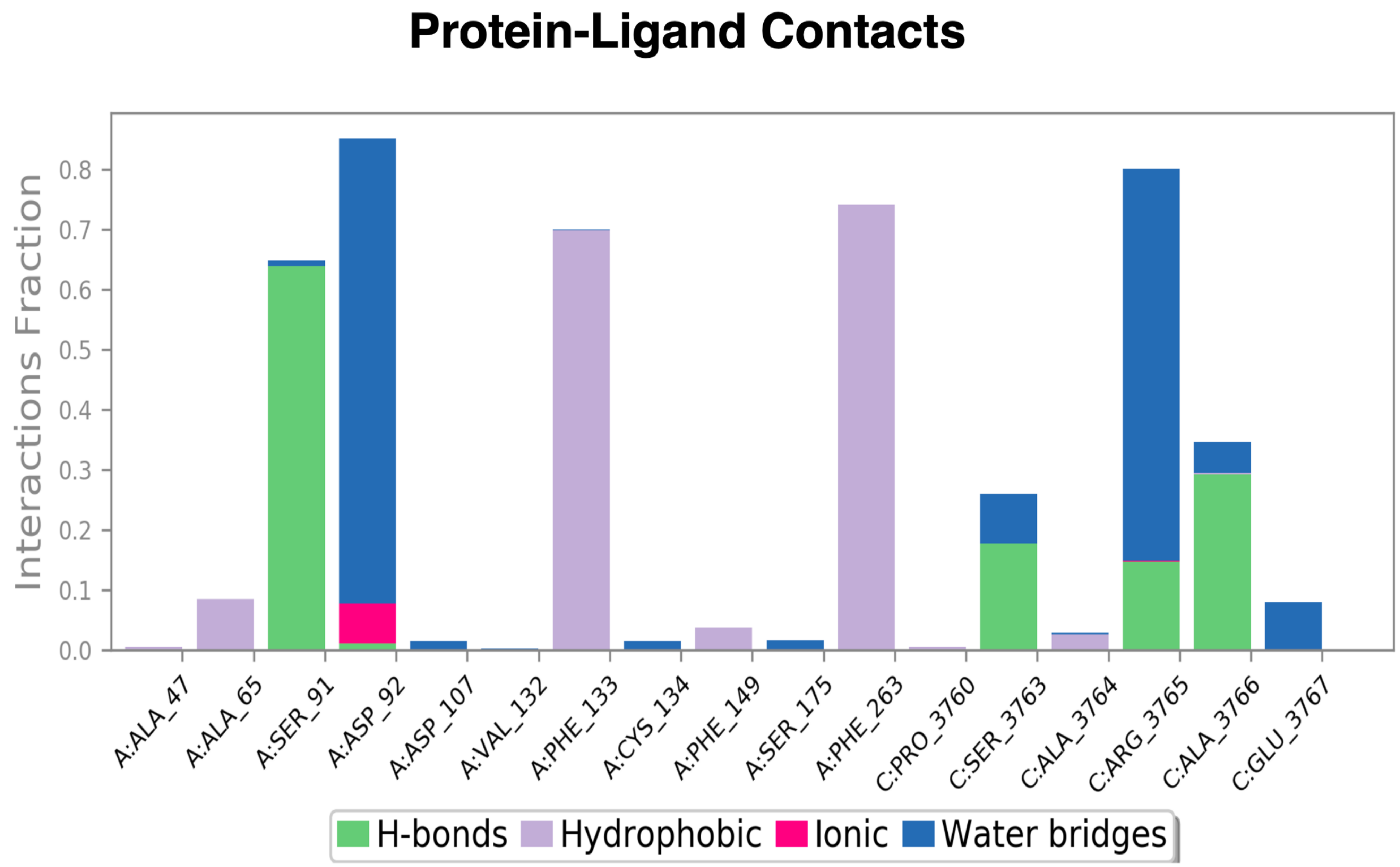

Figure S18. The bar chart illustrates the interactions formed between the drug IA9 and the WDR5-MLL1 complex, highlighting hydrogen bonds, hydrophobic interactions, ionic bonds, and water bridges during 250 ns molecular simulations.

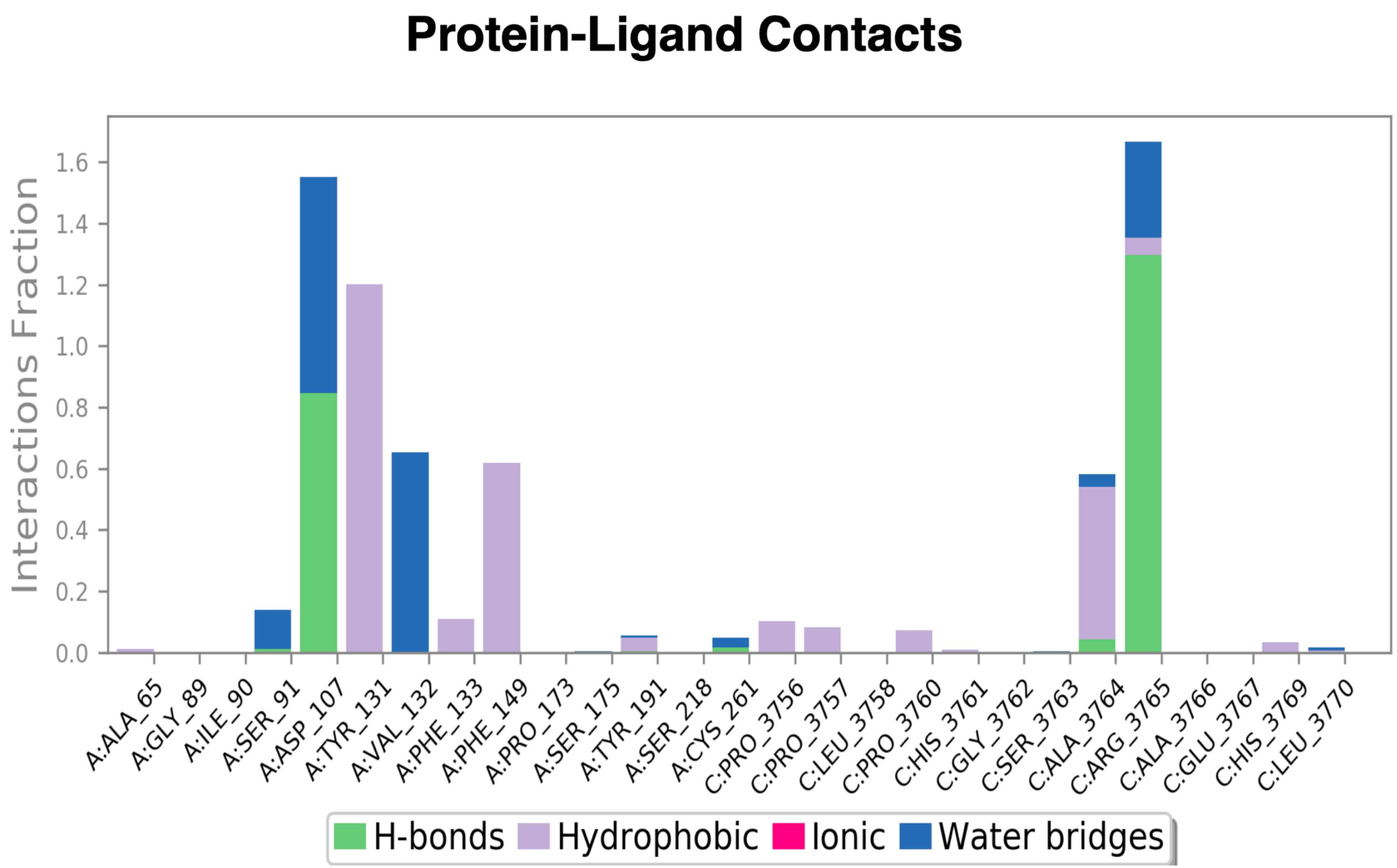

Figure S19. The bar chart illustrates the interactions formed between the drug Z88418521 and the WDR5-MLL1 complex, highlighting hydrogen bonds, hydrophobic interactions, ionic bonds, and water bridges during 250 ns molecular simulations.

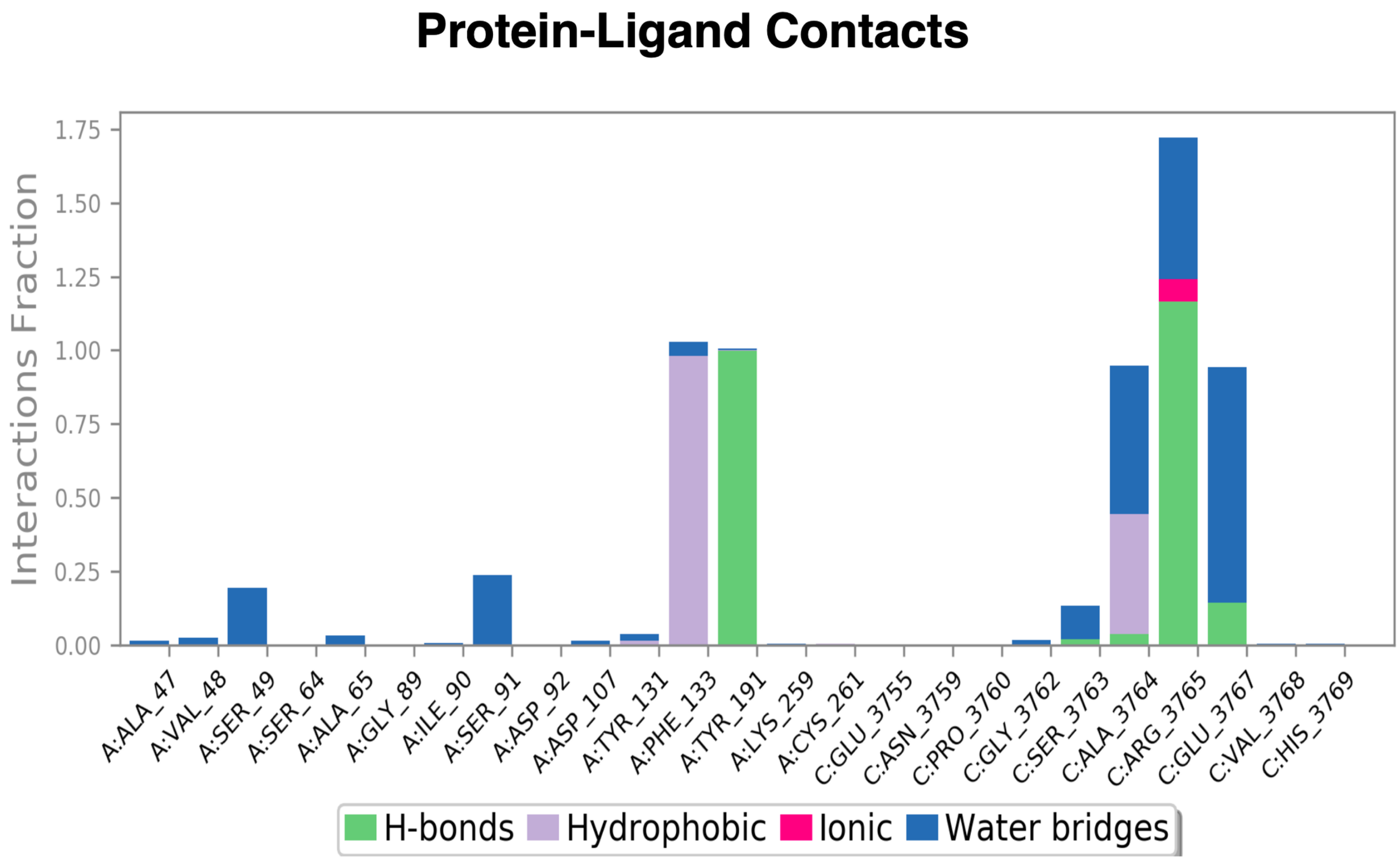

Figure S20. The bar chart illustrates the interactions formed between the drug Z116334910 and the WDR5-MLL1 complex, highlighting hydrogen bonds, hydrophobic interactions, ionic bonds, and water bridges during 250 ns molecular simulations.

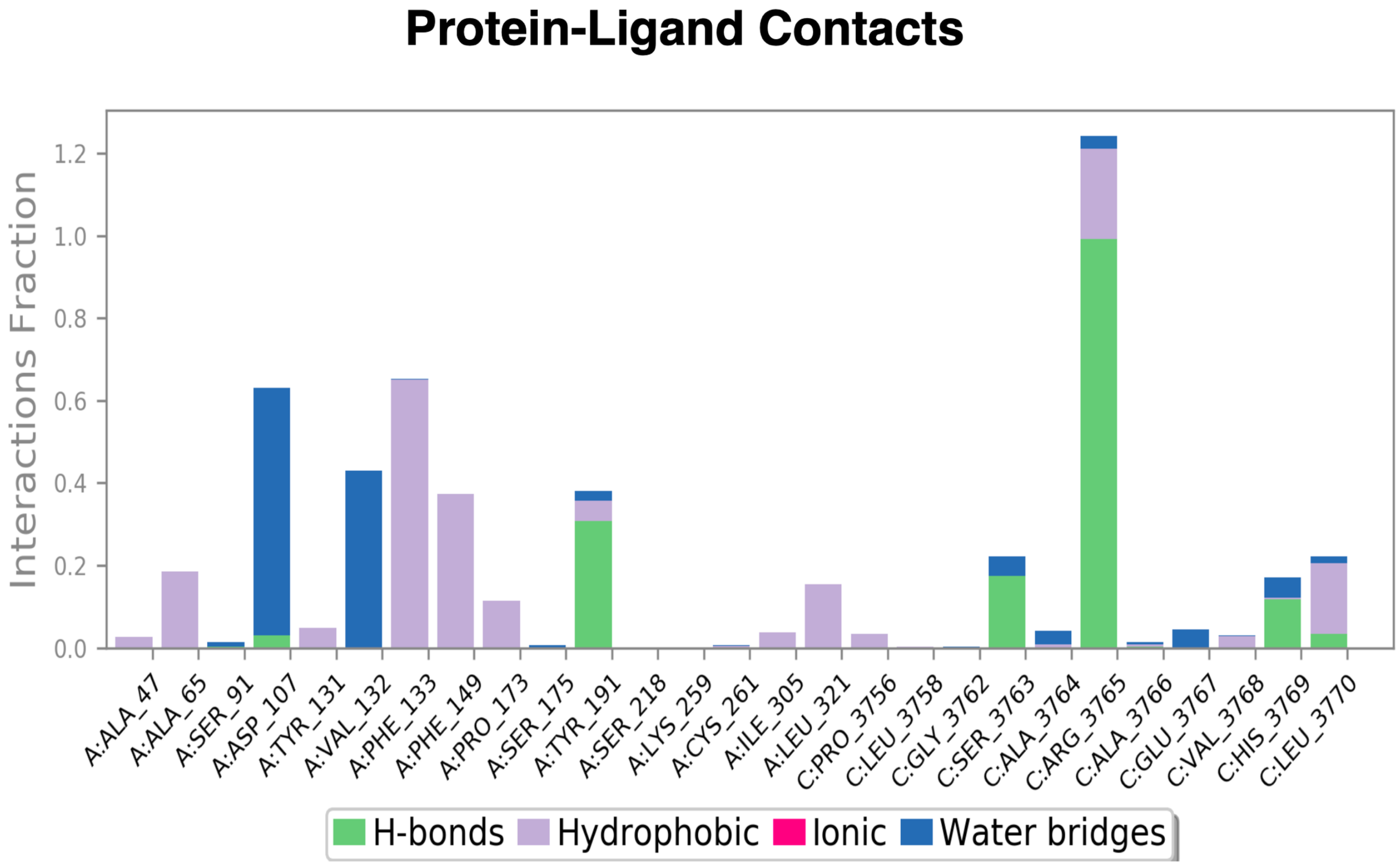

Figure S21. The bar chart illustrates the interactions formed between the drug Z118783062 and the WDR5-MLL1 complex, highlighting hydrogen bonds, hydrophobic interactions, ionic bonds, and water bridges during 250 ns molecular simulations.

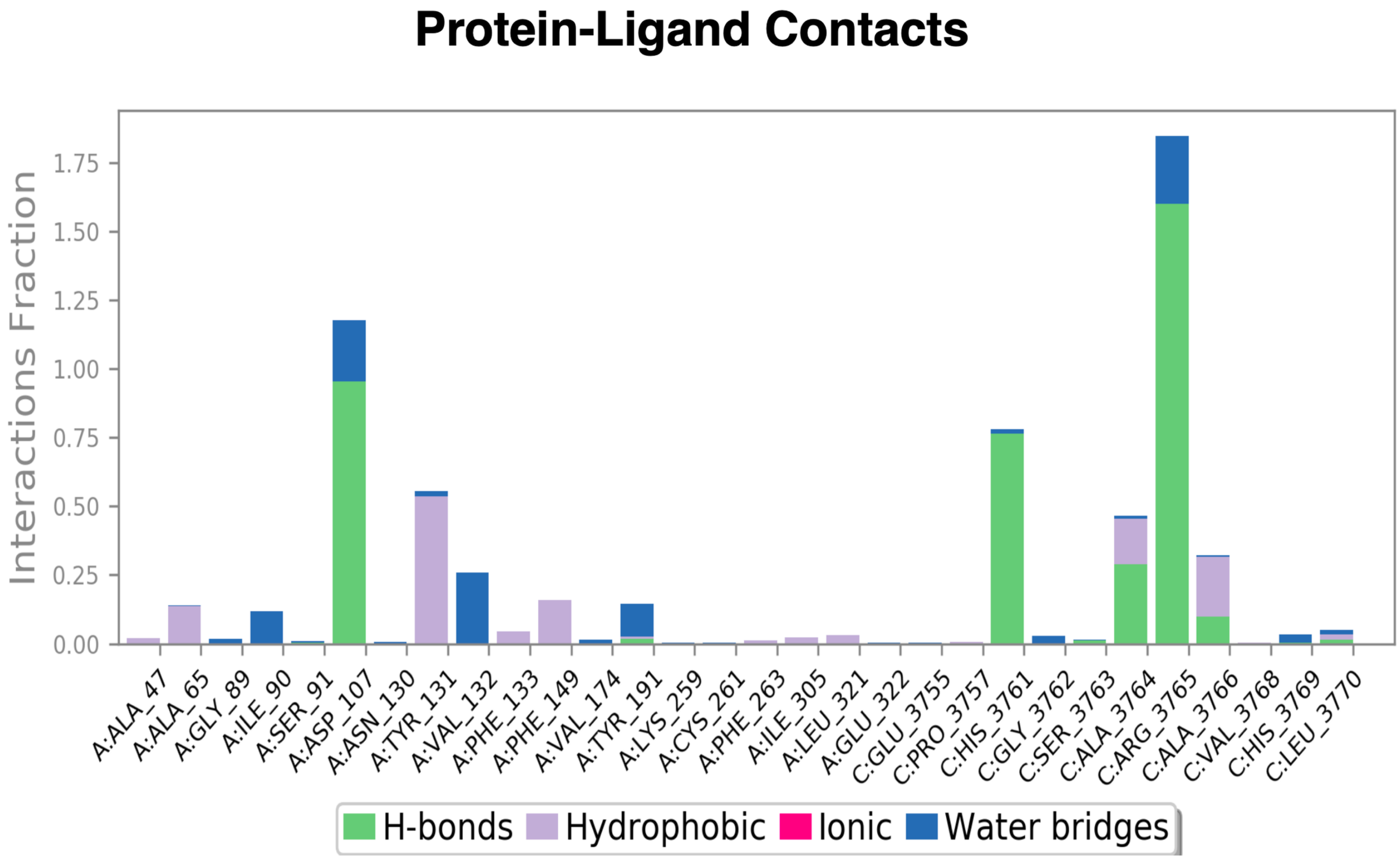

Figure S22. The bar chart illustrates the interactions formed between the drug Z997046664 and the WDR5-MLL1 complex, highlighting hydrogen bonds, hydrophobic interactions, ionic bonds, and water bridges during 250 ns molecular simulations.

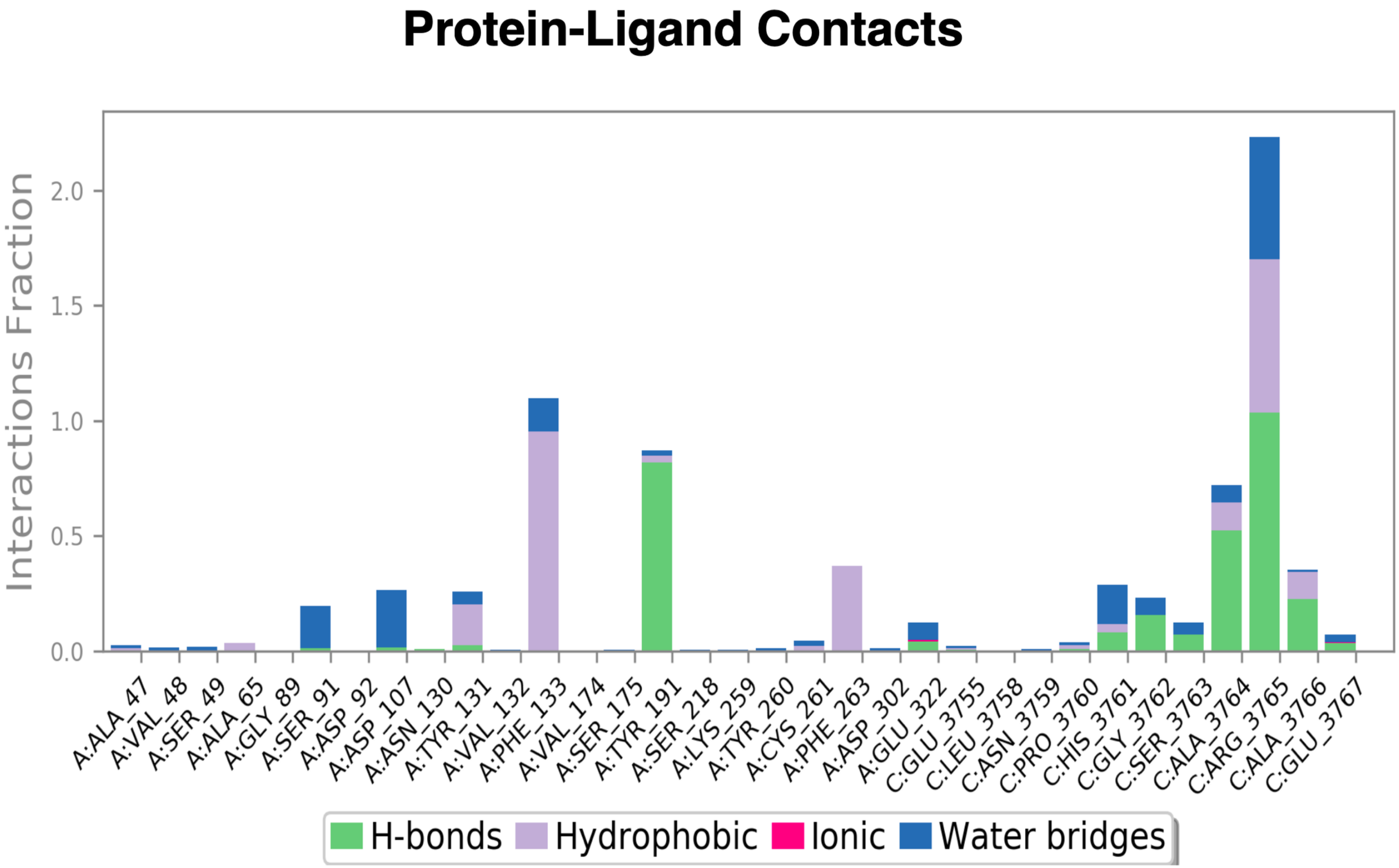

Figure S23. The bar chart illustrates the interactions formed between the drug Z1098417322 and the WDR5-MLL1 complex, highlighting hydrogen bonds, hydrophobic interactions, ionic bonds, and water bridges during 250 ns molecular simulations.
